# Chromatographically separable rotamers of an unhindered amide

**DOI:** 10.3762/bjoc.10.63

**Published:** 2014-03-21

**Authors:** Mario Geffe, Lars Andernach, Oliver Trapp, Till Opatz

**Affiliations:** 1 Institut für Organische Chemie, Johannes Gutenberg Universität Mainz, Duesbergweg 10–14, 55128 Mainz, Germany; 2 Organisch-Chemisches Institut, Ruprecht-Karls Universität Heidelberg, Im Neuenheimer Feld 270, 69120 Heidelberg, Germany

**Keywords:** amides, density functional calculations, dynamic HPLC, rotamers, thermodynamics

## Abstract

Surprisingly stable formamide rotamers were encountered in the tetrahydroisoquinoline and morphinan series of alkaloids. We investigated the hindered rotation around the amide bond by dynamic high-performance liquid chromatography (DHPLC) and kinetic measurements of the interconversion of the rotamers which can readily be separated by HPLC as well as TLC. The experimental results of the different methods were compared to each other as well as to results obtained by DFT calculations.

## Introduction

The hindered rotation about the amide bond belongs to the most classical concepts taught to every undergraduate chemistry student. However, amide rotamers are generally classified as conformers which interconvert at ambient temperature unless a significant steric hindrance has to be overcome. This is illustrated by another classical textbook example, the coalescence of the NMR signals of *N,N*-dimethylformamide, which can be brought about by gentle warming. Separable amide rotamers are usually regarded as laboratory curiosities and are not expected in the absence of sterical congestion.

During synthetic work in the morphinan series, we encountered a remarkable behavior of an unhindered formamide which produced double bands in TLC. We initially attributed this behavior to the undesired formation of diastereomers as even at 150 °C, no coalescence of the ^1^H NMR signals of the formyl protons could be observed. However, when the same effect was seen in compound **4** possessing only a single stereocenter, the occurrence of highly stable rotamers had to be taken into consideration. Indeed, no change in *R*_f_-value of the two discrete spots for compound **4** was observed in a two-dimensional TLC experiment in which the second development immediately followed the air-drying of the plate. If one hour at room temperature lay between the first and the second development, two weak off-diagonal spots indicated that interconversion of both species had taken place to a small extent. When the temperature was increased to 75 °C during a 15 min drying period, four spots with roughly equal intensity resulted from the second 2D-TLC run and indicated complete interconversion.

A look into the literature revealed that the occurrence of separable amide rotamers of 1-benzyl-*N*-formyl-1,2,3,4-tetrahydroisoquinolines had been reported independently by Rice, Brossi [1–2] and Szántay [3]. During their investigation of 6'-bromo-*N*-formylnorreticuline, they observed the expected doubling of signals in the ^1^H NMR spectra as well as a separation of the *E-* and *Z-*rotamers on TLC. Furthermore, they were able to isolate both rotamers in pure form by crystallization. While Rice and Brossi focused on the optical and crystallographic properties of these compounds, Szántay et al. gave a first estimate of the activation energy of the interconversion of these two rotamers based on dynamic NMR spectroscopy at variable temperature. They deduced a value of 94 kJ/mol from a coalescence temperature of 170 °C but did not provide crucial data such as the spectrometer frequency required for the calculation. Sulima et al. reported a rotational barrier of 92 kJ/mol for 1-bromo-*N*-formyl-4-hydroxy-3-methoxymorphinan-6-one based on dynamic NMR while the separation of the enthalpic and entropic contributions to this value was not possible with this method [4].

## Results and Discussion

### Synthesis

**Scheme 1:**
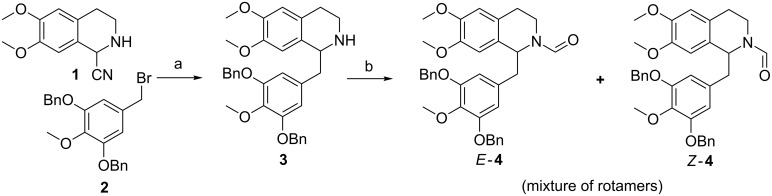
Synthesis of formamide **4**. Reagents and conditions: a) KHMDS, THF, −78 °C; then: NaBH_4_, MeOH, 63%. b) HCOOEt, reflux, quant.

Compound **4** was prepared by C-alkylation of the potassium salt of α-aminonitrile **1** with benzyl bromide **2** utilizing methodology established by our group for the syntheses of various isoquinoline alkaloids [5–7]. Spontaneous dehydrocyanation afforded the 1-benzylated 3,4-dihydroisoquinoline which was subsequently reduced in situ to tetrahydroisoquinoline **3** in a one-pot procedure with sodium borohydride in 63% yield. *N*-Acylation was effected quantitatively by refluxing **3** in ethyl formate (Scheme 1).

Dynamic NMR (DNMR) measurements (400 MHz) on a sample of **4** in DMSO-*d*_6_ at temperatures ranging from 20 °C to 150 °C, the upper limit for technical reasons, showed no signs of beginning coalescence of the formyl proton resonances even at the highest temperature. This very high rotational barrier prompted us to look for alternative methods which allow the accurate determination of the thermodynamic parameters of the bond rotation. An estimate of the interconversion rate at ambient temperature was obtained from an experiment in which both rotamers of **4** were separated by HPLC and their reequilibration was followed with the same technique.

### Kinetic studies

**Figure 1:**
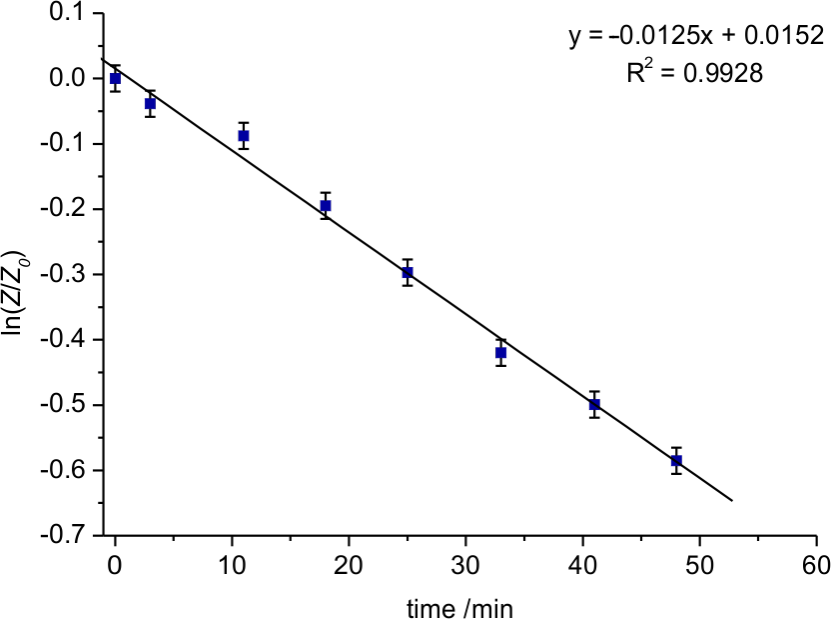
Equilibration of the *Z*-rotamer of **4** at 293 K.

**Figure 2:**
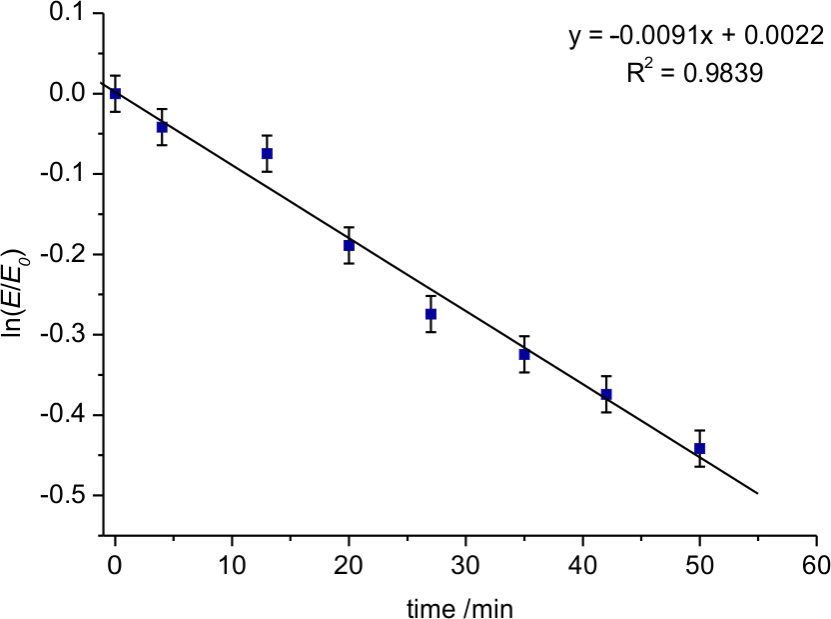
Equilibration of the *E*-rotamer of **4** at 293 K.

The kinetic studies were performed at 20 °C on a Knauer normal-phase HPLC (see Supporting Information File 1 for details) using hexane/2-propanol 80:20 as mobile phase. Each rotamer was collected separately and reinjected at defined intervals (7–8 minutes) to follow the interconversion. Equilibrium was reached after 2 h and the data of the first 50 min were used to determine the initial rate. The integrals were used to quantify the amounts of each rotamer and were plotted as ln(A/A_0_) vs time. The slope of the linear regression equates the rate constant *k*_1_ for a first order interconversion and by using the Eyring equation


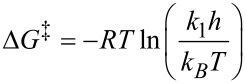


(with *R* = gas constant, *T* = temperature, *h* = Planck constant, *k_B_* = Boltzmann constant) the rotational barrier was calculated to Δ*G*^‡^ (293 K) = 92.4 ± 0.1 kJ/mol (*Z* to *E*) and Δ*G*^‡^ (293 K) = 93.2 ± 0.1 kJ/mol (*E* to *Z*) respectively (Figure 1, Figure 2).

### Dynamic HPLC studies

**Figure 3:**
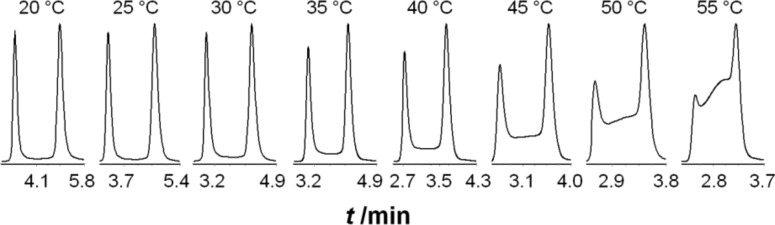
Elution profiles obtained by temperature-dependent DHPLC measurements of **4**.

Like the DNMR data, the simple kinetic analysis only permits the determination of the free activation energy while the separation of entropic and enthalpic contributions requires a variation of the temperature. Therefore, dynamic high-performance liquid chromatography (DHPLC) [8–10] was performed at temperatures between 20 °C and 55 °C. The obtained elution profiles were characterized by distinct plateau formation between the well separated peaks of the *Z*- and *E*-rotamers (first and later eluted isomer, respectively), indicating the interconversion during the partitioning process (Figure 3).

Reaction rate constants *k*_1_ were determined using the unified equation, which has been described in detail in the literature [11–15]. Calculations were performed with the software DCXplorer [16], which has implemented the unified equation.

**Figure 4:**
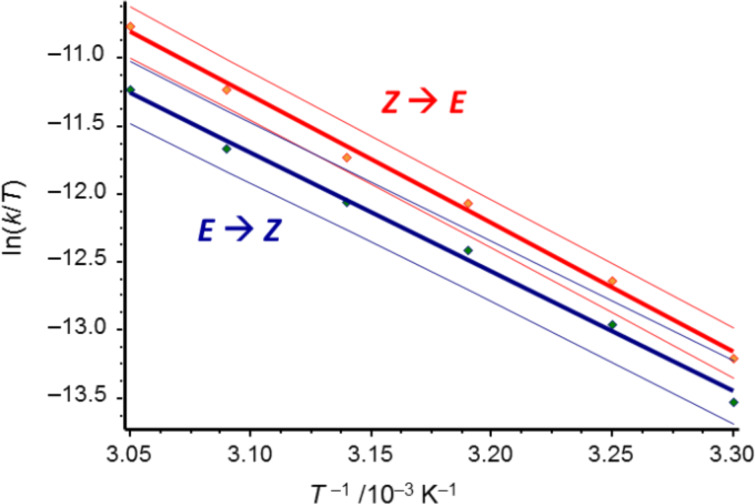
Eyring plot obtained by temperature dependent DHPLC measurements of **4**.

For the evaluation of activation parameters of the interconversion process experiments between 30 °C and 55 °C were considered, because at lower temperatures the plateau height was too low to be determined with high precision. The Gibbs free activation energy Δ*G*^‡^ (*T*) was calculated according to the Eyring equation (vide supra). The activation enthalpy Δ*H*^‡^ of the interconversion process was obtained from the slope and the activation entropy Δ*S*^‡^ from the intercept of the Eyring plot (Figure 4). Deviations of the activation parameters Δ*H*^‡^ and Δ*S*^‡^ have been calculated by error band analysis of the linear regression with a level of confidence of 95%.

The barriers and activation parameters for the interconversion of the *Z*-rotamer to the *E*-rotamer were determined to be Δ*G*^‡^ (293 K) = 92.5 kJ/mol, Δ*H*^‡^ = 78.3 ± 1.8 kJ/mol and Δ*S*^‡^ = −49 ± 2 J/(K mol) (R^2^ = 0.9958, residual deviation σ_y_ = 0.0560), and for the interconversion of the *E*-rotamer to the *Z*-rotamer Δ*G*^‡^ (293 K) = 93.1 kJ/mol, Δ*H*^‡^ = 73.2 ± 2.3 kJ/mol and Δ*S*^‡^ = −68 ± 4 J/(K mol) (R^2^ = 0.9950, residual deviation σ_y_ = 0.0575). These data are in very good agreement with the data obtained by conventional reaction progress analysis.

### Computational studies

**Figure 5:**
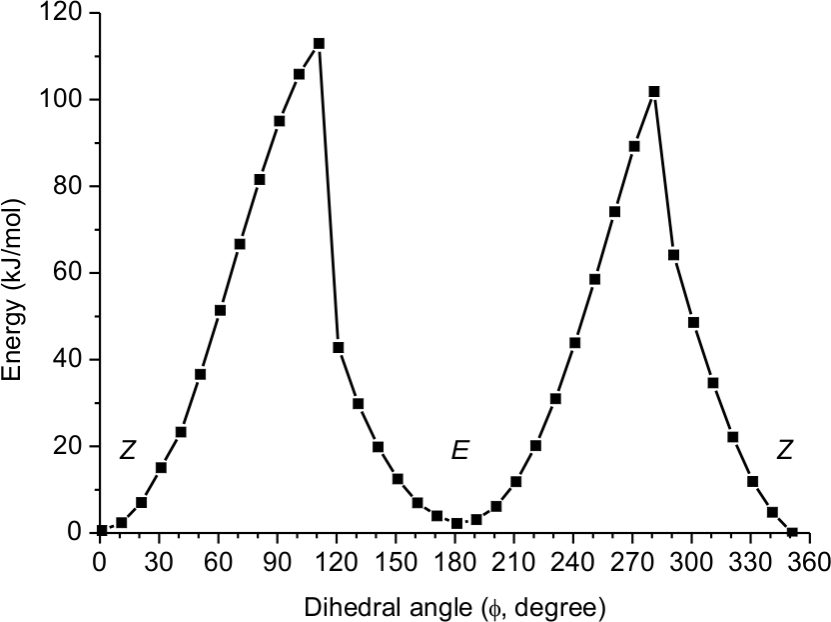
Potential energy surface scan graph for the dihedral angle O–C–N–C1 (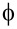) in **4** (BP-D3/def2-SVP).

The rotational barrier was also studied in silico. Therefore, a conformational analysis of **4** was performed using the systematic algorithm to search conformers as implemented in Spartan’10 with the semi-empirical PM6 level of theory [17–18]. All 2111 resulting conformers were subjected to a DFT geometry optimization at the BP-D3/def2-SVP [19–22] level of theory with ORCA [23–24]. The BP functional was chosen because it was found to reproduce the energy difference between the ground states better than B3LYP and PBE for this molecule. With the lowest energy conformer, a potential energy surface (PES) scan for the rotation around the C–N bond in steps of 10° was done. This scan provided two local minima as well as two maxima (Figure 5). The asymmetric peak shape is caused by the inversion of the pyramidal nitrogen between 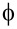 = 110° and 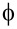 = 120° as well as 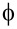 = 280° and 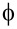 = 290°. The geometries of both minima were used as starting geometries for a geometry optimization followed by the calculation of the thermochemical data at 298 K. The coordinates of the maxima were used as starting points for the search for the transition states. After locating the transition state geometries, their thermochemical data at 298 K were calculated. The PES scan and all subsequent calculations were also performed using COSMO solvation for hexane.

The final values for the relative Gibbs free energies for *E*-**4**, *Z*-**4**, TS1, and TS2 are given in Table 1. The difference between the *E*- and *Z*-isomer of **4** is predicted to be 3.2 kJ/mol in hexane and 5.8 kJ/mol in vacuo, respectively. The transition states are located at 108.8 kJ/mol (TS1) and 105.5 kJ/mol (TS2) above the *Z*-isomer of **4** for hexane solvation as well as at 107.3 k/mol (TS1) and 102.1 kJ/mol (TS2) in the gas phase, respectively.

**Table 1:** Relative Gibbs free energy (kJ/mol) for *E*-**4**, *Z*-**4**, TS1, TS2, GS_DMF_, TS1_DMF_ and TS2_DMF_ in the gas phase and with COSMO solvation for hexane, ethanol and DMSO at 298 K (BP-D3/def2-SVP).

	gas phase	hexane	ethanol	DMSO

*Z*-**4**	0	0	0	0
TS1	+107.3	+108.8	+109.0	+110.0
TS2	+102.1	+105.5	+109.8	+110.4
*E*-**4**	+5.8	+3.2	+2.7	+3.1
GS_DMF_	0	0	0	0
TS1_DMF_	+99.7	+100.8	+103.7	+103.9
TS2_DMF_	+102.0	+104.4	+110.2	+110.7

**Figure 6:**
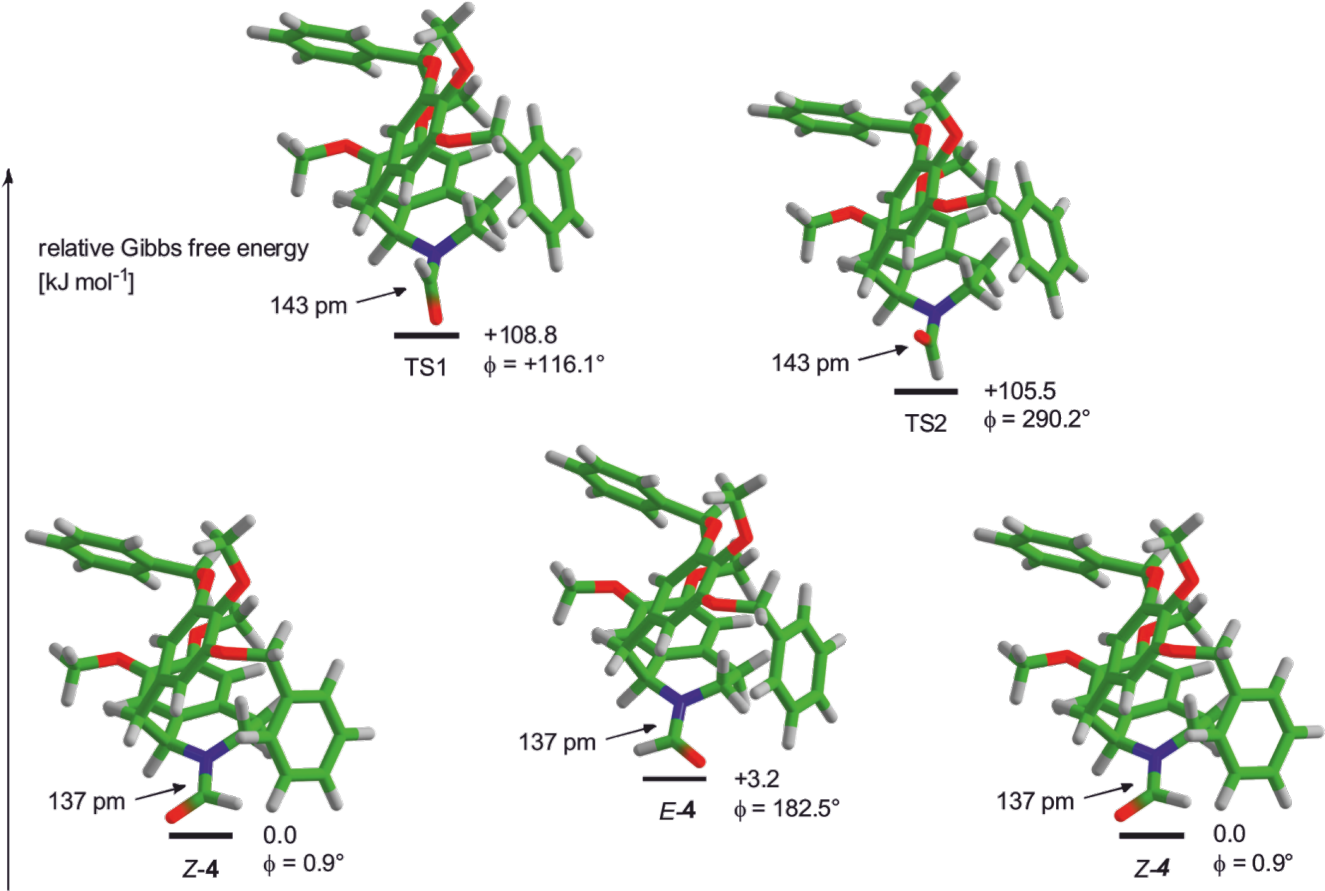
Energy differences and geometries for *E*- and *Z*-**4** and both transition states (TS1 and TS2) in hexane. The dihedral angle of O–C–N–C1 (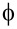) and the C–N bond length are given for each state (BP-D3/def2-SVP).

The geometries of the *E-* and *Z*-ground states of **4** and both transition states (TS1 and TS2) are shown in Figure 6 together with the dihedral angle O–C–N–C1 (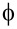) and the C–N bond length. Both transition states show a single imaginary frequency (−280.8 cm^−1^ (TS1) and −364.5 cm^−1^ (TS2) in hexane and at −284.0 cm^−1^ (TS1) and −362.2 cm^−1^ (TS2) in the gas phase). This imaginary frequency belongs to the rotational vibration of the formyl hydrogen and the formyl oxygen along the reaction pathway for the *E*/*Z* isomerization of **4**. In both transition states geometries, the C–N bond (143 pm) is significantly elongated compared to the ground states (137 pm). Furthermore, the nitrogen atom shows a pyramidal instead of a trigonal planar geometry. This reflects the expected decrease of the C–N bond order caused by lacking overlap of the π*-orbital of the carbonyl group and the lone pair at nitrogen.

Unfortunately, the free energy of activation for the rotation of **4** is overestimated by at least 10 kJ/mol. A possible explanation for this difference could be the high basicity of the pyramidal nitrogen in both transition states which may favor the formation of hydrogen bonds lowering the total energy. Other reasons for this overestimation may be an inappropriate representation of the global solvation by the COSMO model or the limited double-zeta basis set, the substitution of which by a larger triple-zeta basis set is too expensive in terms of computational time. Increasing the solvent polarity in COSMO up to pure ethanol or DMSO did however not improve the results. Sulima et al. reported a similar deviation for the calculated rotational barrier in their studies of a morphinan-derived formamide [4] using comparable parameters for their calculations (B3LYP/6-31G*) and obtained a highly similar energy profile.

While in their case only a small energy difference between the *E*- and the *Z*-ground states was predicted in silico and confirmed experimentally, our DFT results deviate more significantly from the HPLC and NMR data which show the *E*-form and not the *Z*-form to be lower in energy by 0.6–0.8 kJ/mol. The larger size of the molecule and its considerable conformational freedom may contribute to this deviation. Calculations on the model compound 1-benzyl-*N*-formyl-1,2,3,4-tetrahydroisoquinoline at the same level of theory showed that either the *E*- or the *Z*-ground state can be lower in energy depending on the relative arrangement of the benzyl substituent. For comparison, the rotational barrier for DMF was calculated using the same functional and basis set (Table 1) and was found to be overestimated by 18–22 kJ/mol based on 82.6 kJ/mol as the value for the experimental barrier in cyclohexane solution [25]. With the B3LYP functional this deviation is reduced to 15–18 kJ/mol. However, we used the BP functional since the energy difference between the *E*- and the *Z*-ground states of **4** is better reflected.

## Conclusion

The energetic barrier for the rotation about the C–N-bond of a 1-benzyl-*N*-formyl-1,2,3,4-tetrahydroisoquinoline giving two separable rotamers at 20 °C was measured using HPLC kinetics as well as dynamic HPLC at variable temperature with good agreement. The latter technique allowed the determination of the enthalpic and entropic contributions for both directions of the interconversion. DFT-calculations overestimated the rotational barrier while dynamic NMR did not prove useful as no signs of coalescence could be detected up to 150 °C. Therefore, dynamic HPLC is a valuable alternative to dynamic NMR and provides full thermodynamic data for reversible interconversions.

## Supporting Information

File 1 Experimental procedures, HPLC chromatograms, copies of 1D and 2D NMR spectra of compounds **3** and **4**, atom coordinates and DFT energies for ground- and transition states.
