# Supplementary material for: Chromatographically separable rotamers of an unhindered amide
Source: Beilstein J Org Chem. 2014 Mar 21;10:701–6. doi: 10.3762/bjoc.10.63 (PMC3999880; doi:10.3762/bjoc.10.63)
Supplement: File 1 — Experimental procedures, HPLC chromatograms, copies of 1D and 2D NMR spectra of compounds 3 and 4, atom coordinates and DFT energies for ground- and transition states. [file Beilstein_J_Org_Chem-10-701-s001.pdf]

Supporting Information

for

Chromatographically separable rotamers of an unhindered amide

Mario Geffe[1], Lars Andernach[1], Oliver Trapp[2] and Till Opatz[*1]

Address: [1]Institut für Organische Chemie, Johannes Gutenberg Universität Mainz, Duesbergweg 10–14, 55128 Mainz, Germany and [2]Organisch-Chemisches Institut, Ruprecht-Karls Universität Heidelberg, Im Neuenheimer Feld 270, 69120 Heidelberg, Germany

Email: Till Opatz - opatz@uni-mainz.de

* Corresponding author

Experimental procedures, HPLC chromatograms, copies of 1D and 2D NMR spectra of compounds 3 and 4, atom coordinates and DFT energies for ground- and transition states

1. General information

All reagents were reagent grade and used without further purification unless otherwise noted. All reactions involving air or moisture sensitive reagents or intermediates were performed under an inert atmosphere of argon in glassware that was oven dried. Tetrahydrofuran was distilled from potassium and benzophenone. Reaction temperatures referred to the temperature of the particular cooling/heating bath. Chromatography was performed using flash chromatography of the indicated solvent system on 35–70 μm silica gel (Acros Organics) unless otherwise indicated. ^{1}H NMR and ^{13}C NMR spectra were recorded on a Bruker Avance-II 400 MHz or Bruker Avance-III 600 MHz spectrometer. Chemical shifts were referenced to the deuterated solvent (e.g., for $CDCl_3$, δ = 7.26 ppm and 77.16 ppm for ^{1}H and ^{13}C NMR, respectively) and reported in parts per million (ppm, δ) relative to tetramethylsilane (TMS, δ = 0.00 ppm). Coupling constants (*J*) were reported in Hz and the splitting abbreviations used were: s, singlet; d, doublet; t, triplet; m, multiplet; br, broad. Reactions were monitored by thin-layer chromatography (TLC) carried out on 0.25 mm E. Merck silica gel plates ($60F_{254}$) using UV light as visualizing agent. High-resolution masses were recorded on a Waters QTof-Ultima 3-Instrument with Lockspray-Interface and a suitable external calibrant. Infrared spectra were recorded as FTIR spectra using a diamond ATR unit and are reported in terms of frequency of absorption (ν, cm^{-1}). Chromatographic separation and DHPLC-measurements were performed using a Knauer system with a K-1001 pump, a column oven and a K-2800 diode array UV-detector.

2. Syntheses

Synthesis of 1-(3,5-bis(benzyloxy)-4-methoxybenzyl)-6,7-dimethoxy-1,2,3,4-tetrahydroisoquinoline (**3**)

A solution of KHMDS (1.995 g, 10.0 mmol) in dry THF (22 mL) was added at −78 °C under argon to a solution of **1** (1.091 g, 5.0 mmol) in dry THF (33 mL). After 5 min, a solution of **2** (2.273 g, 5.5 mmol) in dry THF (33 mL) was added and stirring was continued for 2 h at −78 °C and 2 h at room temperature. The reaction mixture was diluted with 40 mL of methanol followed by the addition of sodium borohydride (472.9 mg, 12.5 mmol). After stirring overnight the solvents were removed in vacuum, the residue was taken on in sat. $NaHCO_3$ solution (30 mL) and extracted with DCM (3 × 20 mL). The organic phase was dried (Na_2SO_4), concentrated under reduced pressure and purified by flash column chromatography (silica gel, CH/EE/Et_2NH = 6:3:1) to yield **3** (1.651 g, 63%) as colorless oil.

^{1}H NMR HSQC, HMBC (400 MHz, $CDCl_3$) δ 7.45 – 7.41 (m, 4 H, 2 x 2-H, 6-H Bn), 7.40 – 7.34 (m, 4 H, 2 x 3-H, 5-H Bn), 7.33 – 7.28 (m, 2 H, 2 x 4-H Bn), 6.58 (s, 1 H, 8-H), 6.58 (s, 1 H, 5-H), 6.48 (s, 2 H, 2´-H, 6´-H), 5.11 (s, 4 H, 2 x CH_2 Bn), 4.05 (dd, *J* = 9.0, 4.3 Hz, 1 H, 1-H), 3.90 (s, 3 H, 4´-OCH_3), 3.85 (s, 3 H, 7-OCH_3), 3.81 (s, 3 H, 8-OCH_3), 3.14 – 3.01 (m, 2 H, 3-H, CH_2), 2.85 – 2.73 (m, 2 H, 3-H, CH_2), 2.73 – 2.62 (m, 2 H, 4-H), 1.90 (br s, 1 H, N*H*).

^{13}C NMR HSQC, HMBC (101 MHz, $CDCl_3$) δ 152.6 (C-3´,C-5´, 2 C), 147.6 (7-O*C*H_3), 147.2 (6-O*C*H_3), 138.3 (C-4´), 137.3 (C-1 Bn, 2 C), 134.3 (C-1´), 130.1 (C-8a), 128.7 (C-3 Bn, C-5 Bn, 4 C), 128.0 (C-4 Bn, 2 C), 127.5 (C-4a), 127.4 (C-2 Bn, C-6 Bn, 4 C), 111.9 (C-5), 109.4 (C-8), 109.2 (C-2´, C-6´, 2 C), 71.2 (*C*H_2 Bn, 2 C), 61.1 (4´-O*C*H_3), 56.8 (C-1), 56.1 (C-6), 56.0 (C-7), 42.8 (*C*H_2), 41.0 (C-3), 29.5 (C-4).

IR(ATR) $\nu =$ 2932, 2830, 1586, 1506, 1433, 1373, 1326, 1258, 1222, 1108, 1028, 1008, 906, 857, 781, 736, 697, 592, 548, 467.

ESI-HRMS calcd. for $[C_{33}H_{35}NO_5 + H]^+$ 526.2593, found 526.2611.

Synthesis of *N*-formyl-1-(3,5-bis(benzyloxy)-4-methoxybenzyl)-6,7-dimethoxy-3,4-dihydroisoquinoline (**4**)

3 (445.5 mg, 0.85 mmol) was dissolved in ethyl formate (30 mL) and held under reflux for 16 h (argon atmosphere). Evaporation of excess ethyl formate under reduced pressure gave **4** (473.1 mg, quant.) as a slightly yellow foam.

Z-Rotamer:

^{1}H NMR COSY, NOESY, HSQC, HMBC (600 MHz, DMSO-d$_6$) δ 7.88 (s, 1 H, C*H*O), 7.49 – 7.44 (m, 4 H, 2 x 2-H, 6-H Bn), 7.45 – 7.37 (m, 4 H, 2 x 3-H, 5-H Bn), 7.37 – 7.30 (m, 2 H, 2 x 4-H Bn), 6.74 (s, 1 H, 8-H), 6.68 (s, 1 H, 5-H), 6.52 (s, 2 H, 2´-H, 6´-H), 5.39 (dd, *J* = 8.1, 5.6 Hz, 1 H, 1-H), 5.01 (d, *J* = 5.8 Hz, 4 H, 2 x CH_2 Bn), 3.71 (s, 3 H, 6-OCH_3), 3.68 (s, 3 H, 4´-OCH_3), 3.66 (s, 3 H, 7-OCH_3), 3.51 (ddd, *J* = 13.4, 6.5, 1.9 Hz, 1 H, 3-H), 3.20 (ddd, *J* = 13.2, 11.7, 4.5 Hz, 1 H, 3-H), 3.01 (dd, *J* = 13.8, 5.5 Hz, 1 H, CH_2), 2.90 (dd, *J* = 13.8, 8.2 Hz, 1 H, CH_2), 2.72 – 2.67 (m, 1 H, 4-H), 2.58 (ddd, *J* = 16.1, 4.5, 1.8 Hz, 1 H, 4-H).

^{13}C NMR HSQC, HMBC (151 MHz, DMSO-d$_6$) δ 161.3 (*C*HO), 151.5 (C-3´, C5´, 2 C), 147.6, 147.1, 137.3 (C-1 Bn, 2 C), 136.9 (C-4´), 133.4 (C-1´), 128.5 (C-3, C-5 Bn, 4 C), 127.8 (C-8a), 127.7 (C-4 Bn, 2 C), 127.4 (C-2, C-6 Bn, 4 C), 125.5 (C-4a), 111.8 (C-5), 110.7 (C-8), 108.7 (C-2´, C-6´, 2 C), 70.0 (*C*H_2 Bn, 2 C), 60.1 (4´-O*C*H_3), 55.5 (O*C*H_3), 55.4 (O*C*H_3), 50.7 (C-1), 41.1 (*C*H_2), 39.1 (C-3), 28.6 (C-4).

E-Rotamer:

^{1}H NMR COSY, NOESY, HSQC, HMBC (600 MHz, DMSO-d$_6$) δ 7.57 (s, 1 H, C*H*O), 7.49 – 7.44 (m, 4 H, 2 x 2-H, 6-H Bn), 7.45 – 7.37 (m, 4 H, 2 x 3-H, 5-H Bn), 7.37 – 7.30 (m, 2 H, 2 x 4-H Bn), 6.88 (s, 1 H, 8-H), 6.73 (s, 1 H, 5-H), 6.73 (s, 2 H, 2´-H, 6´-H), 5.08 (d, *J* = 3.9 Hz, 4 H, 2 x CH_2 Bn), 4.78 (dd, *J* = 10.5, 4.4 Hz, 1 H, 1-H), 4.18 (ddd, *J* = 13.2, 5.7, 3.1 Hz, 1 H, 3-H), 3.74 (s, 3 H, 7-

OCH_3), 3.73 (s, 3 H, 6-OCH_3), 3.68 (s, 3 H, 4´-OCH_3), 3.11 (ddd, *J* = 13.1, 10.3, 6.2 Hz, 1 H, 3-H), 3.07 (dd, *J* = 13.8, 4.4 Hz, 1 H, CH_2), 2.85 (dd, *J* = 13.7, 10.6 Hz, 1 H, CH_2), 2.70 – 2.67 (m, 2 H, 4-H).

^{13}C NMR HSQC, HMBC (151 MHz, DMSO) δ 160.8 (*C*HO), 151.9 (C-3´, C5´, 2 C), 147.7, 147.2, 137.2 (C-1 Bn, 2 C), 137.0 (C-4´), 133.7 (C-1´), 128.5 (C-3, C-5 Bn, 4 C), 128.1 (C-8a), 127.7 (C-4 Bn, 2 C), 127.4 (C-2, C-6 Bn, 4 C), 125.6 (C-4a), 112.0 (C-5), 110.5 (C-8), 108.7 (C-2´, C-6´, 2 C), 70.2 (CH_2 Bn, 2 C), 60.2 (4´-OCH_3), 57.2 (C-1), 55.7 (OCH_3), 55.5 (OCH_3), 42.2 (CH_2), 33.0 (C-3), 27.1 (C-4).

E/*Z*-Mixture:

IR(ATR) $\nu =$ 2934, 2832, 1662, 1588, 1506, 1433, 1356, 1326, 1255, 1230, 1190, 1154, 1109, 1007, 980, 904, 845, 785, 737, 697, 674, 648, 592, 546, 466.

ESI-HRMS calcd. for $[C_{34}H_{35}NO_6 + H]^+$ 576.2362, found 576.2360.

3. Kinetic studies

The rotamers were separated using a Macherey-Nagel EC 125/4.6 Nucleodur 100-3 column with a flow of 1.0 mL/min at 20 °C. The eluent was hexane/2-propanol 80:20. Each rotamer was collected separately and reinjected every 7–8 minutes. The integrals were used for the calculation.

**Time /min**	**Area *Z* /a.u.**	**ln (Z/Z_0)**	**Time /min**	**Area *E* /a.u.**	**ln (E/E_0)**
0	100	0	0	100	0
3	96.2	-0.03874083	4	95.9	-0.0418642
11	91.6	-0.08773891	13	92.8	-0.07472355
18	82.3	-0.19479908	20	82.8	-0.18874212
25	74.3	-0.29705923	27	76.0	-0.27443685
33	65.7	-0.42007126	35	72.3	-0.32434606
41	60.7	-0.49922649	42	68.8	-0.37396644
48	55.7	-0.58519004	50	64.3	-0.44161055
55	53.7	-0.62175718	57	64.9	-0.43232256
62	48.9	-0.71539279	64	62.6	-0.46840491
69	48.7	-0.71949116	71	60.8	-0.4975804
76	47.0	-0.75502258	79	60.1	-0.50916034

83	45.8	-0.78088609	86	61.9	-0.47965001
91	46.8	-0.75928698	94	58.9	-0.5293291
99	45.8	-0.78088609	101	58.6	-0.53443549
107	45.5	-0.78745786			
115	43.2	-0.83932969			
123	42.5	-0.85566611			

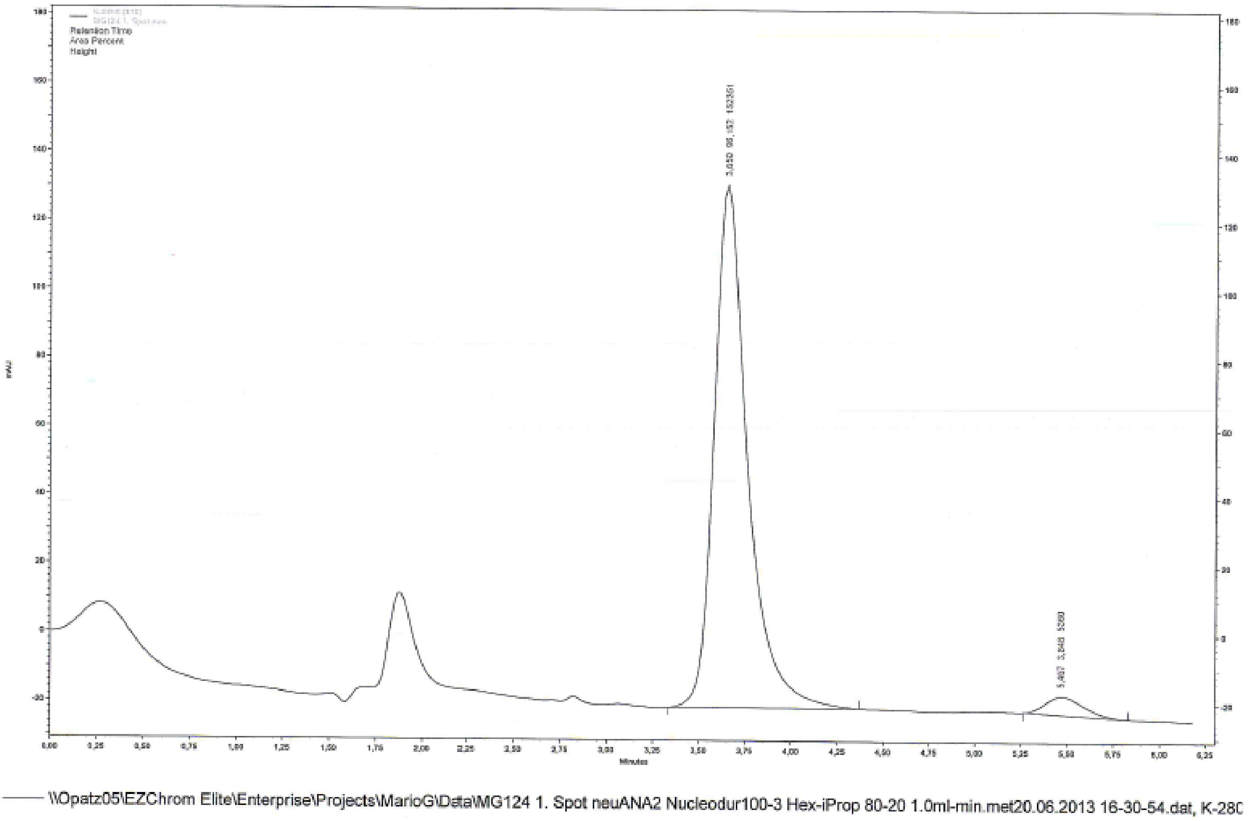

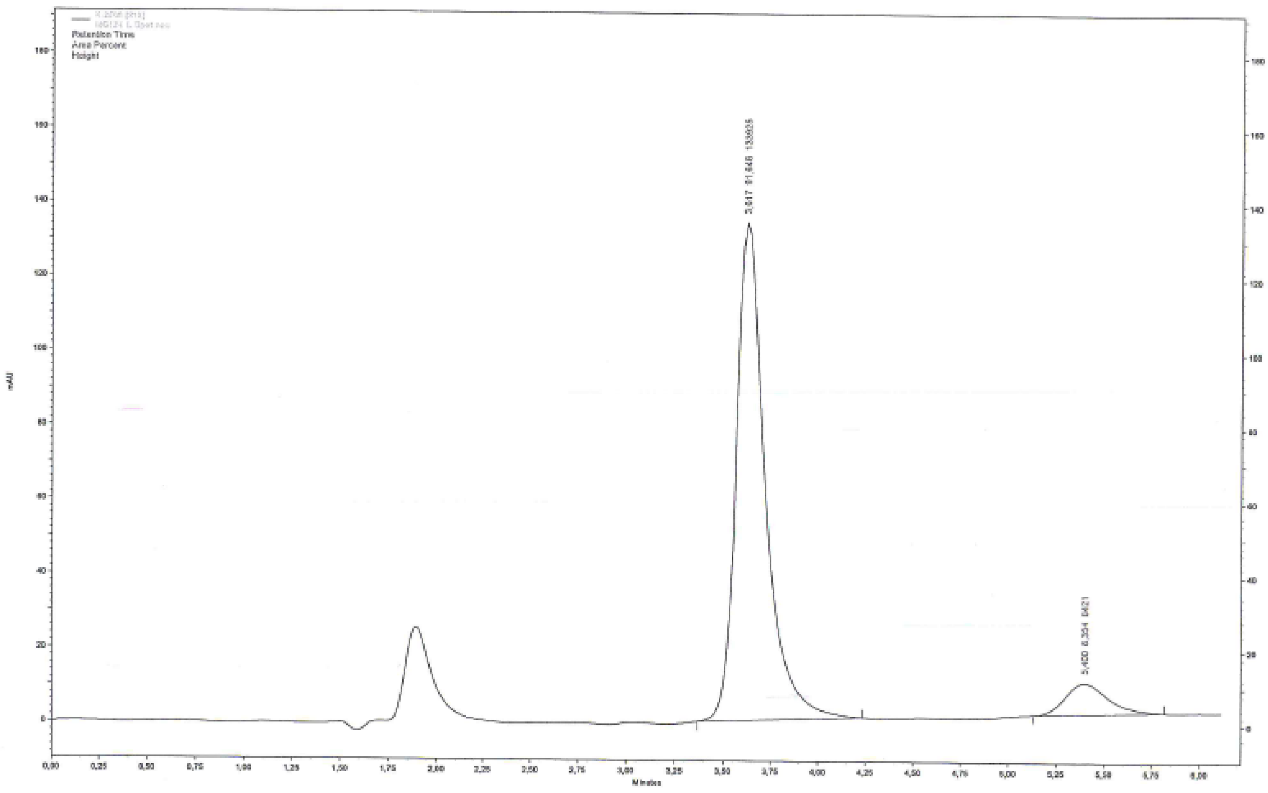

—— \\Opatz05\EZChrom Elite\Enterprise\Projects\MarioG\Data\MG124 1. Spot neuANA2 Nucleodur100-3 Hex-iProp 80-20 1.0ml-min.met20.06.2013 16-38-45.dat, K-280

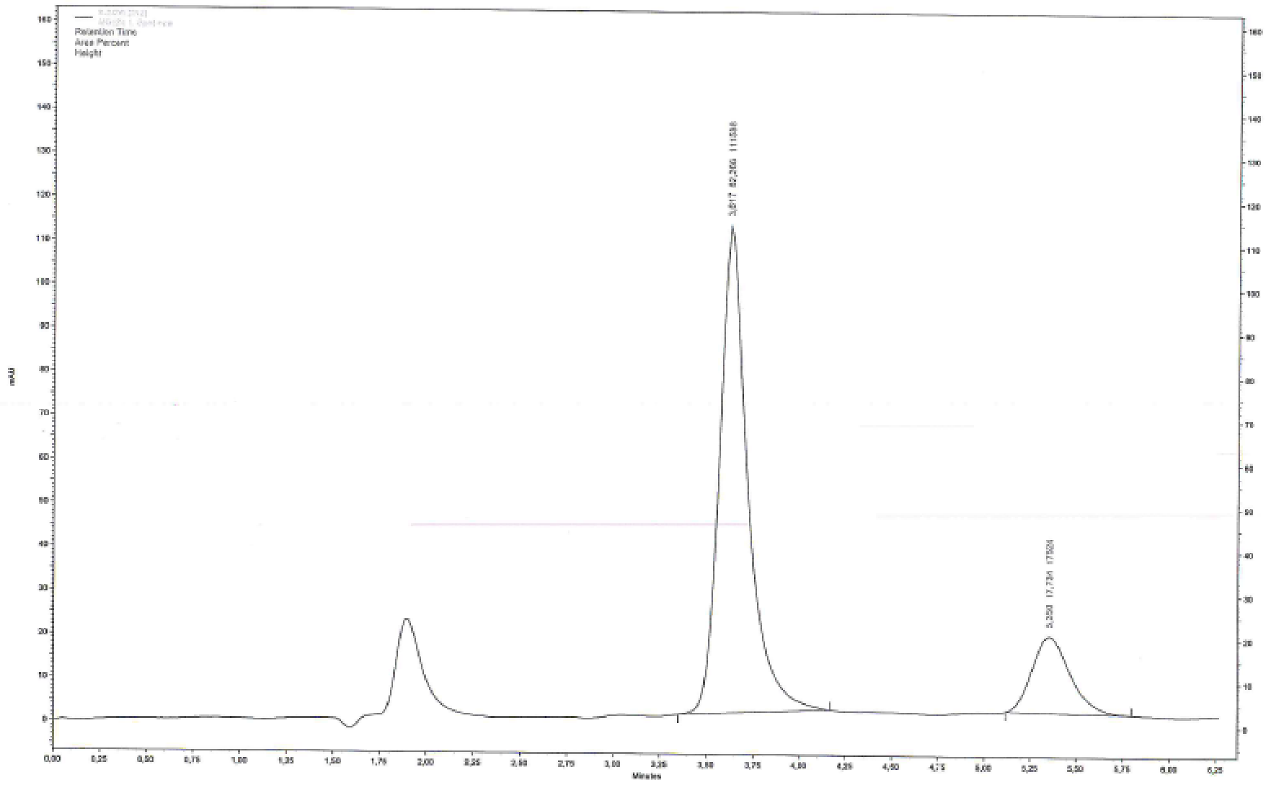

—— \\Opatz05\EZChrom Elite\Enterprise\Projects\MarioG\Data\MG124 1. Spot neuANA2 Nucleodur100-3 Hex-iProp 80-20 1.0ml-min.met20.06.2013 16-46-07.dat, K-280

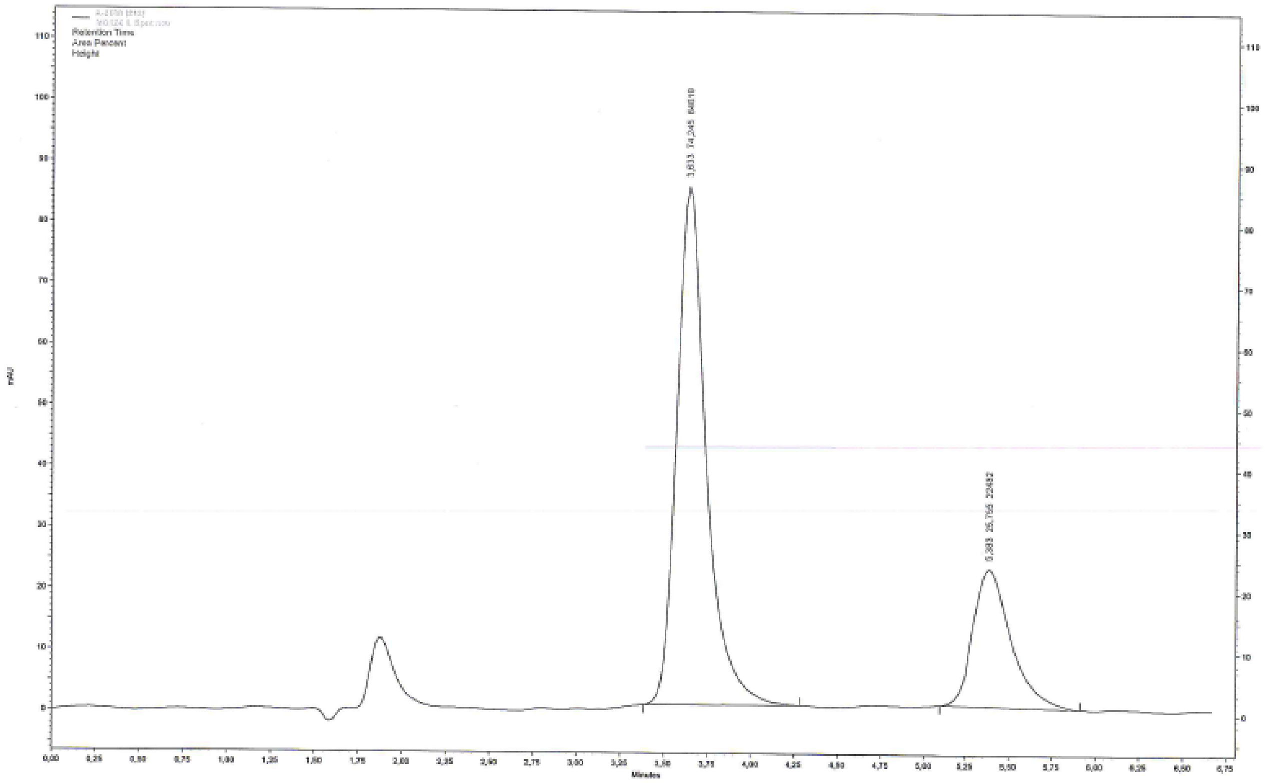

\\Opatz05\EZChrom Elite\Enterprise\Projects\MarioG\Data\MG124 1. Spot neuANA2 Nucleodur100-3 Hex-iProp 80-20 1.0ml-min.met20.06.2013 16-53-29.dat, K-28[illegible]

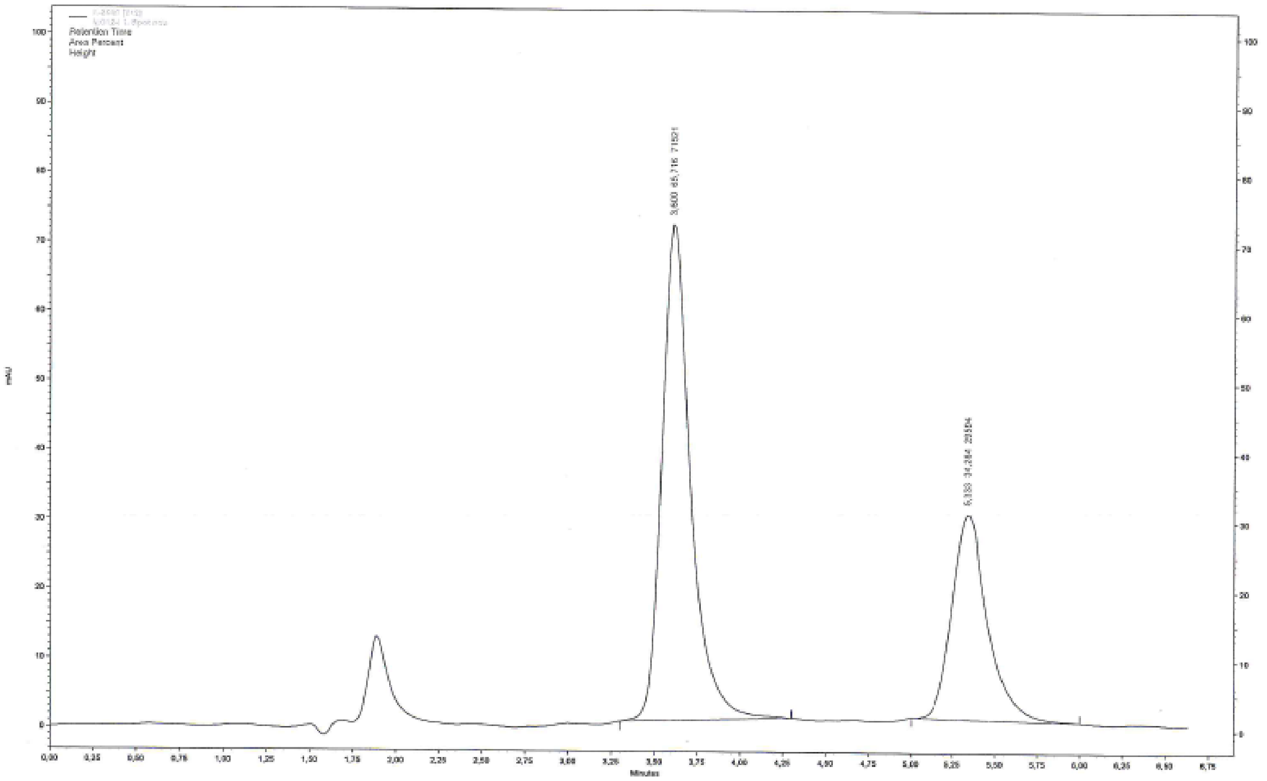

\\Opatz05\EZChrom Elite\Enterprise\Projects\MarioG\Data\MG124 1. Spot neuANA2 Nucleodur100-3 Hex-iProp 80-20 1.0ml-min.met20.06.2013 17-01-13.dat, K-28[illegible]

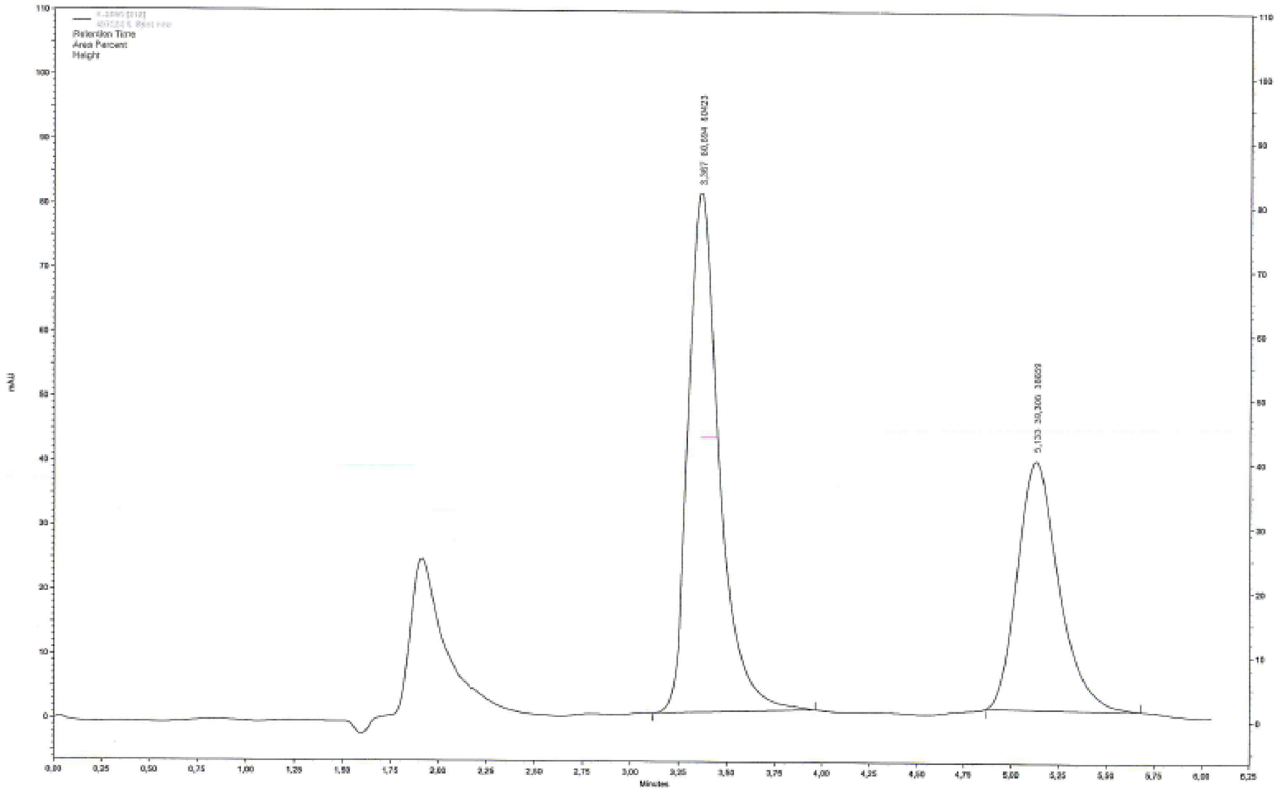

—— \\Opatz05\EZChrom Elite\Enterprise\Projects\MarioG\Data\MG124 1. Spot neuANA2 Nucleodur100-3 Hex-iProp 80-20 1.0ml-min.met20.06.2013 17-09-08.dat, K-280

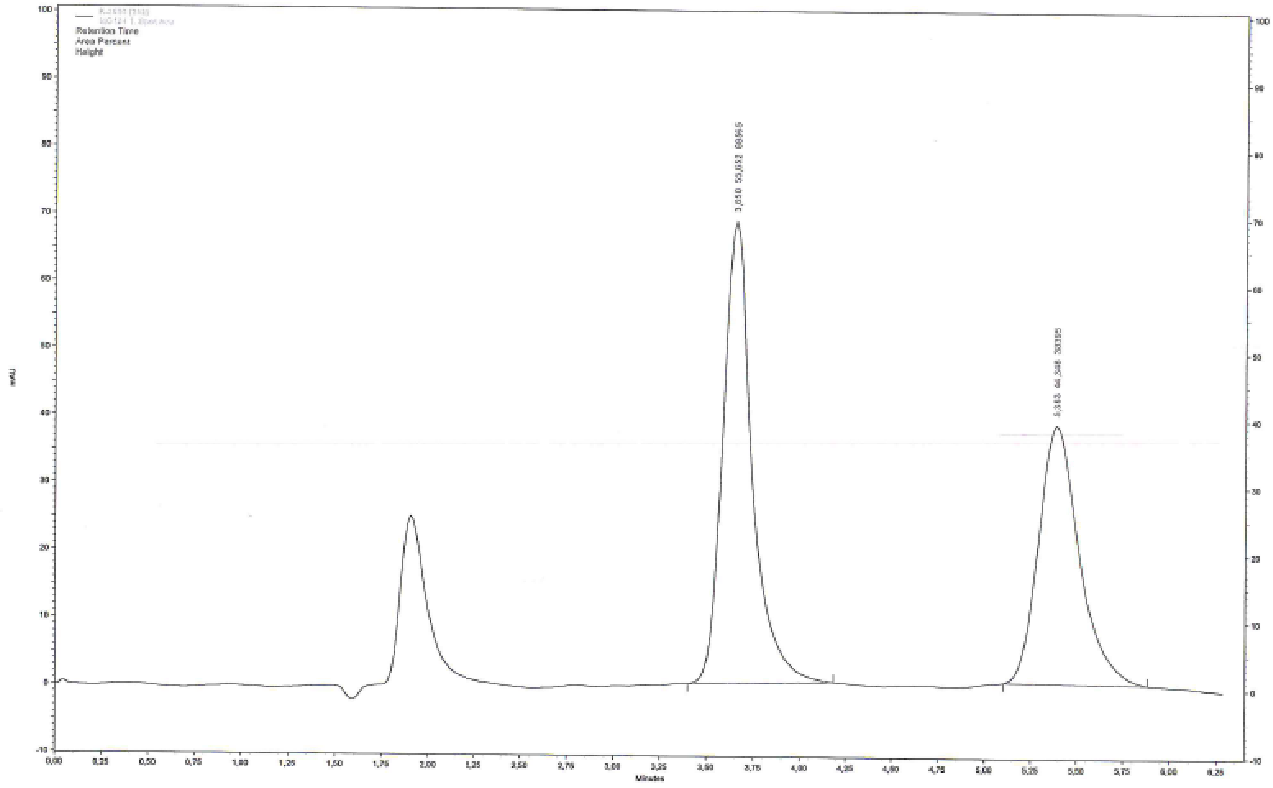

—— \\Opatz05\EZChrom Elite\Enterprise\Projects\MarioG\Data\MG124 1. Spot neuANA2 Nucleodur100-3 Hex-iProp 80-20 1.0ml-min.met20.06.2013 17-16-18.dat, K-280

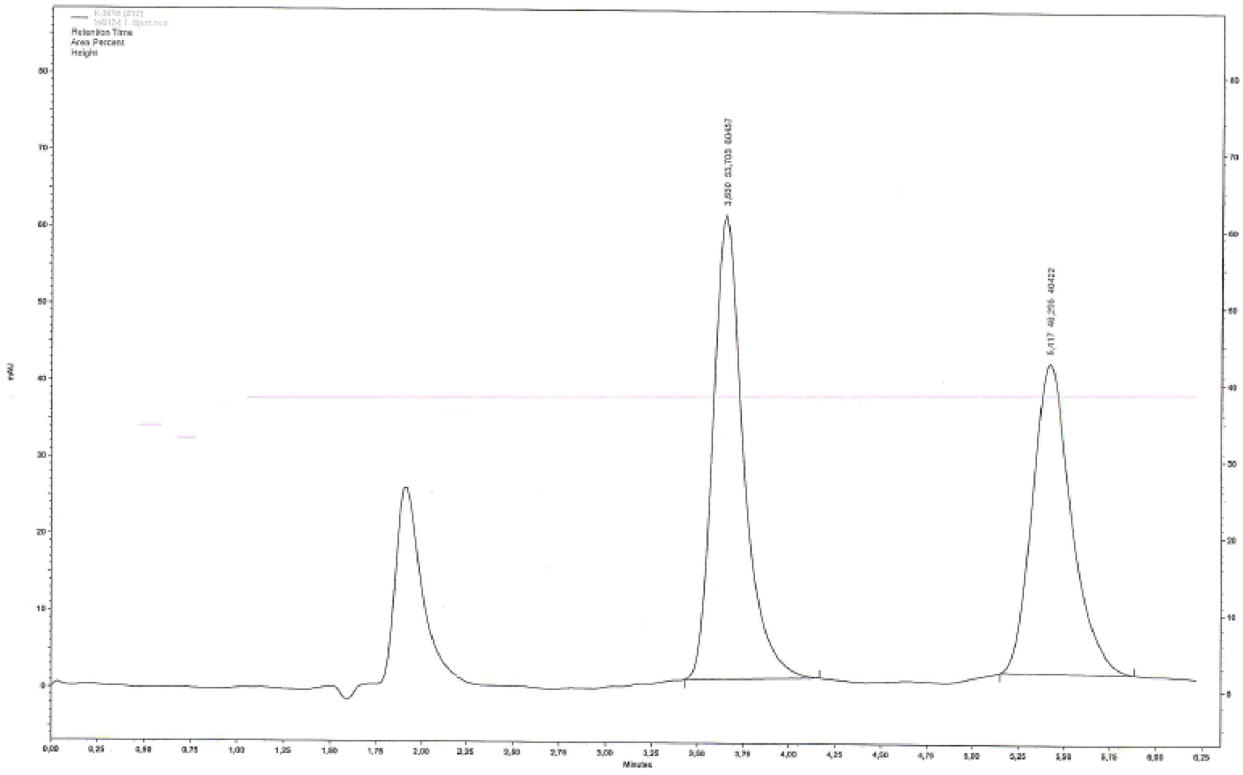

\\Opatz05\EZChrom Elite\Enterprise\Projects\MarioG\Data\MG124 1. Spot neuANA2 Nucleodur100-3 Hex-iProp 80-20 1.0ml-min.met20.06.2013 17-23-37.dat, K-28(

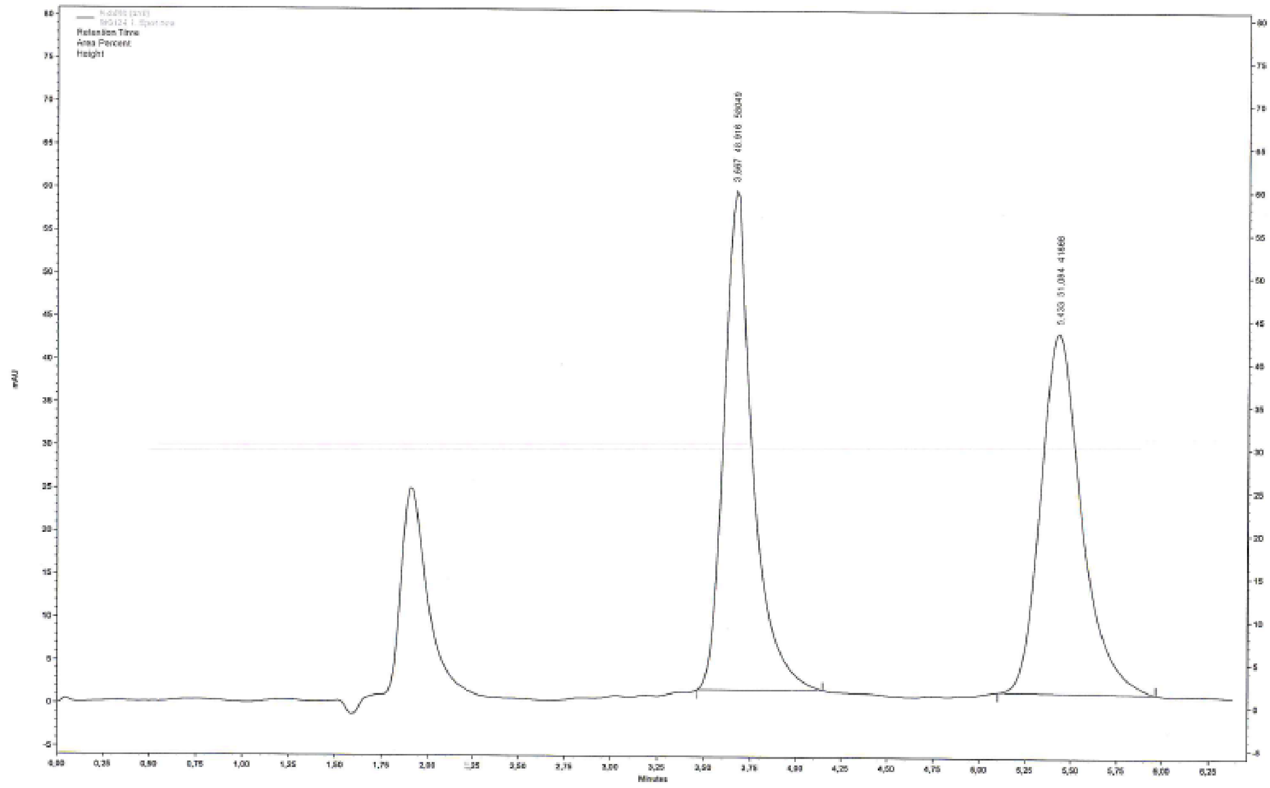

\\Opatz05\EZChrom Elite\Enterprise\Projects\MarioG\Data\MG124 1. Spot neuANA2 Nucleodur100-3 Hex-iProp 80-20 1.0ml-min.met20.06.2013 17-30-59.dat, K-28(

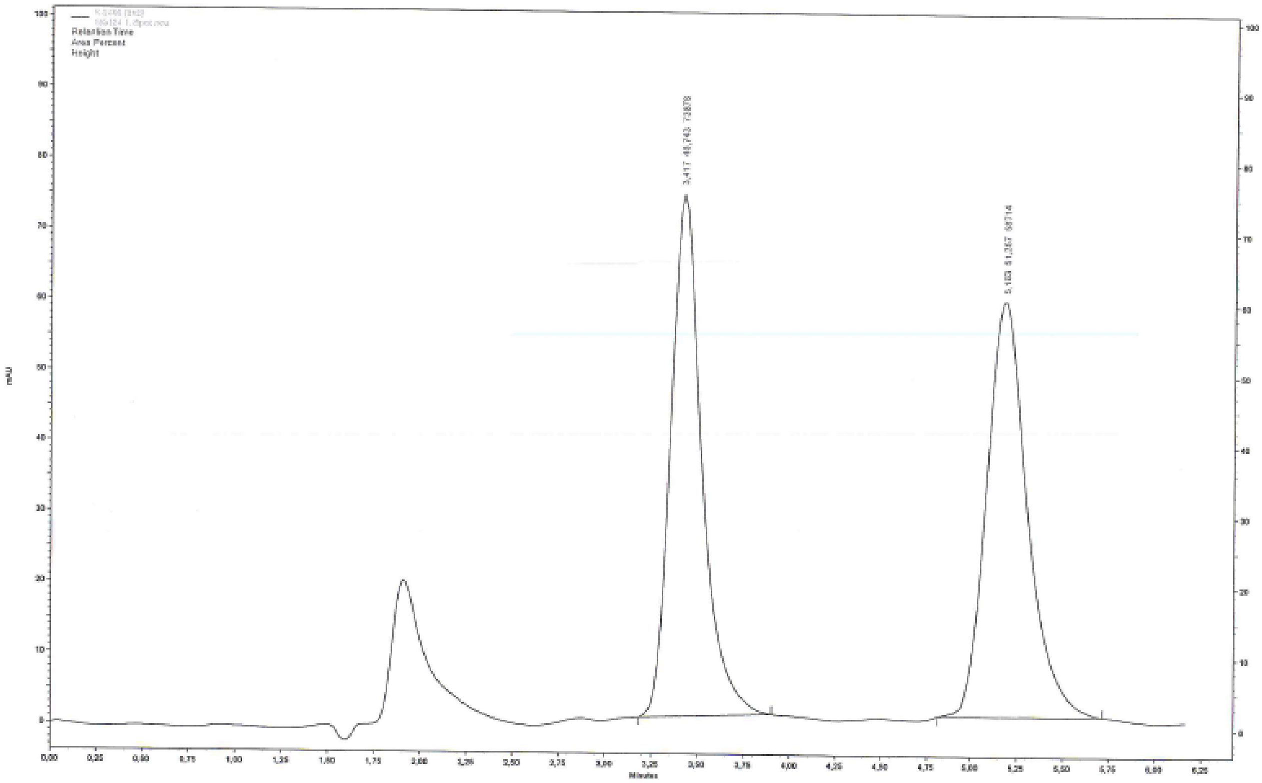

\\Opatz05\EZChrom Elite\Enterprise\Projects\MarioG\Data\MG124 1. Spot neuANA2 Nucleodur100-3 Hex-iProp 80-20 1.0ml-min.met20.06.2013 17-38-20.dat, K-280

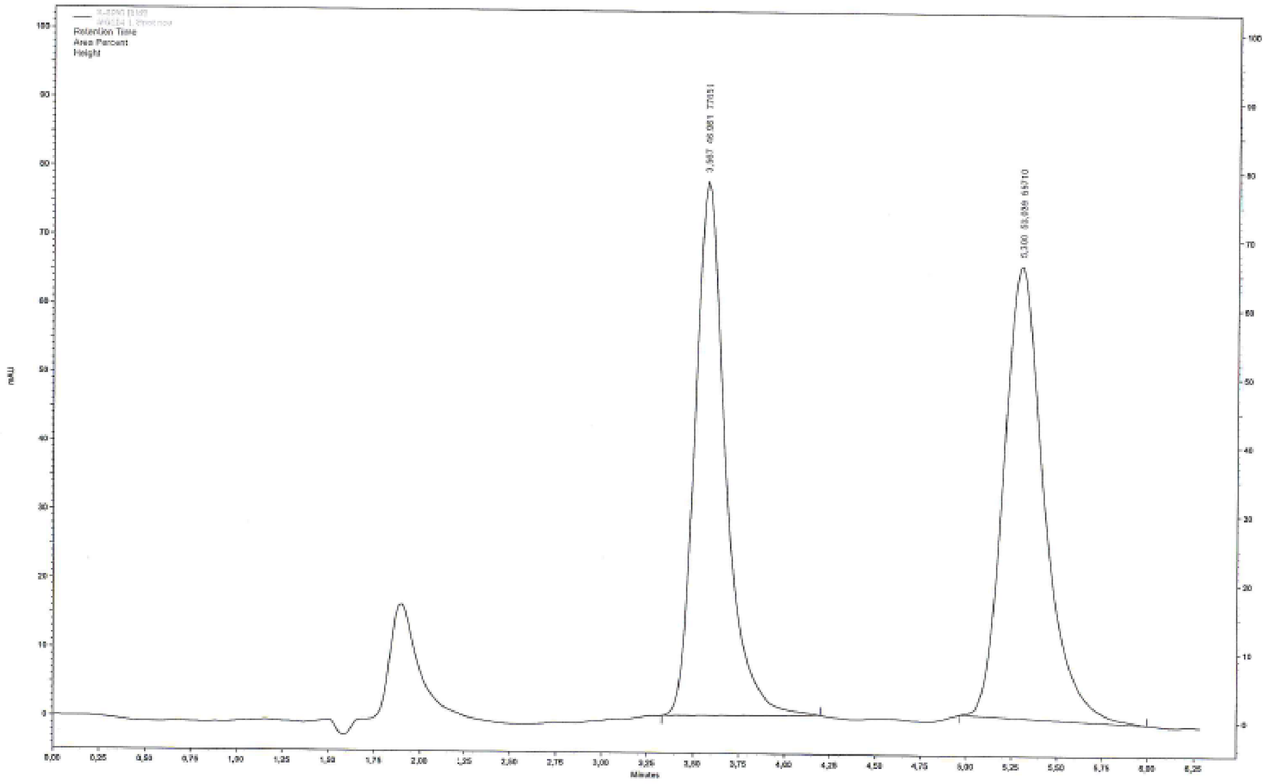

\\Opatz05\EZChrom Elite\Enterprise\Projects\MarioG\Data\MG124 1. Spot neuANA2 Nucleodur100-3 Hex-iProp 80-20 1.0ml-min.met20.06.2013 17-45-44.dat, K-280

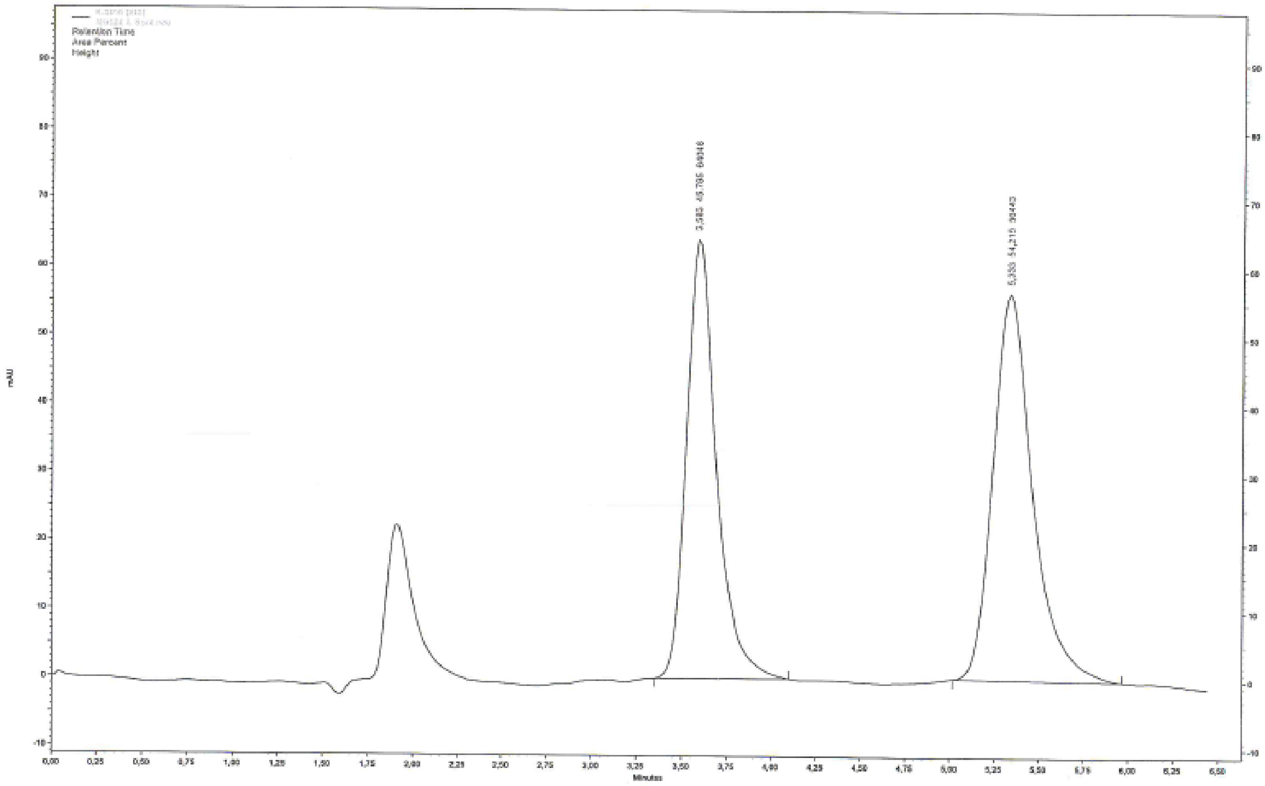

\\Opatz05\EZChrom Elite\Enterprise\Projects\MarioG\Data\MG124 1. Spot neuANA2 Nucleodur100-3 Hex-iProp 80-20 1.0ml-min.met20.06.2013 17-53-11.dat, K-28(

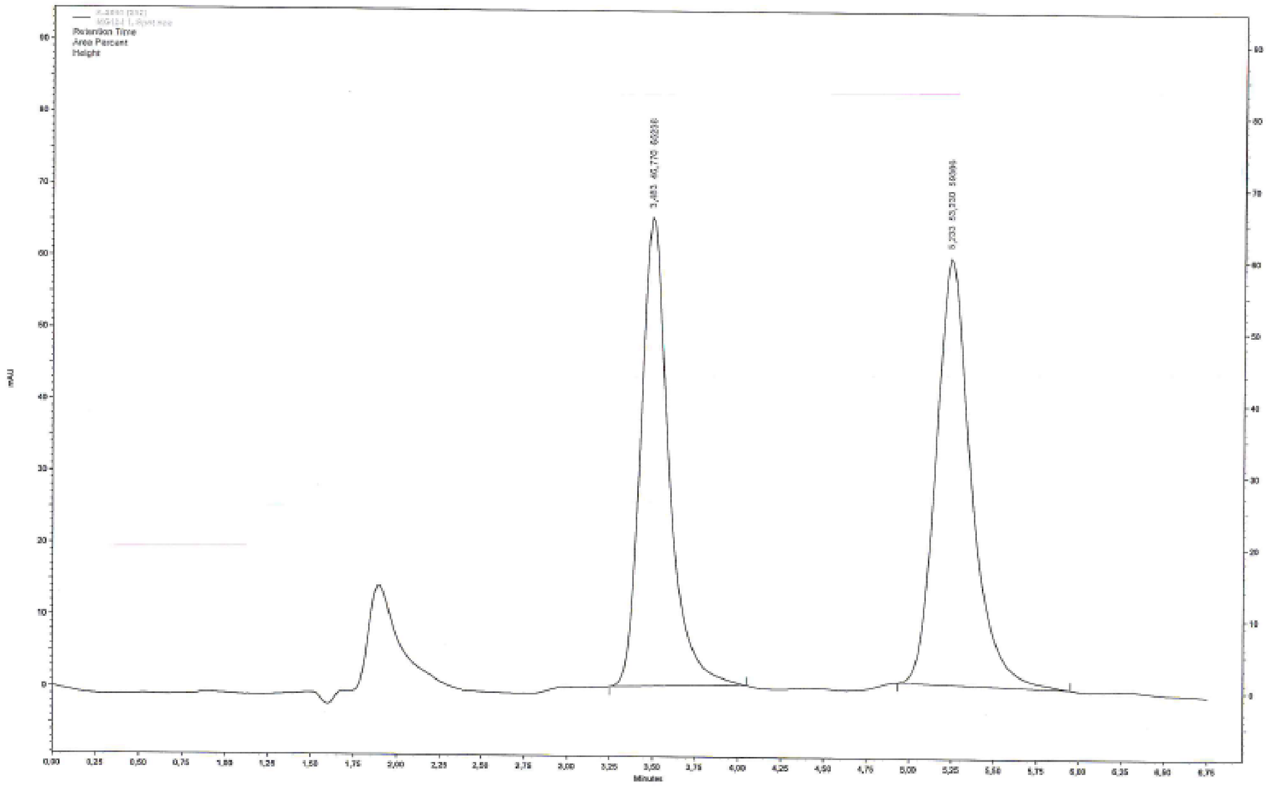

\\Opatz05\EZChrom Elite\Enterprise\Projects\MarioG\Data\MG124 1. Spot neuANA2 Nucleodur100-3 Hex-iProp 80-20 1.0ml-min.met20.06.2013 18-00-50.dat, K-28(

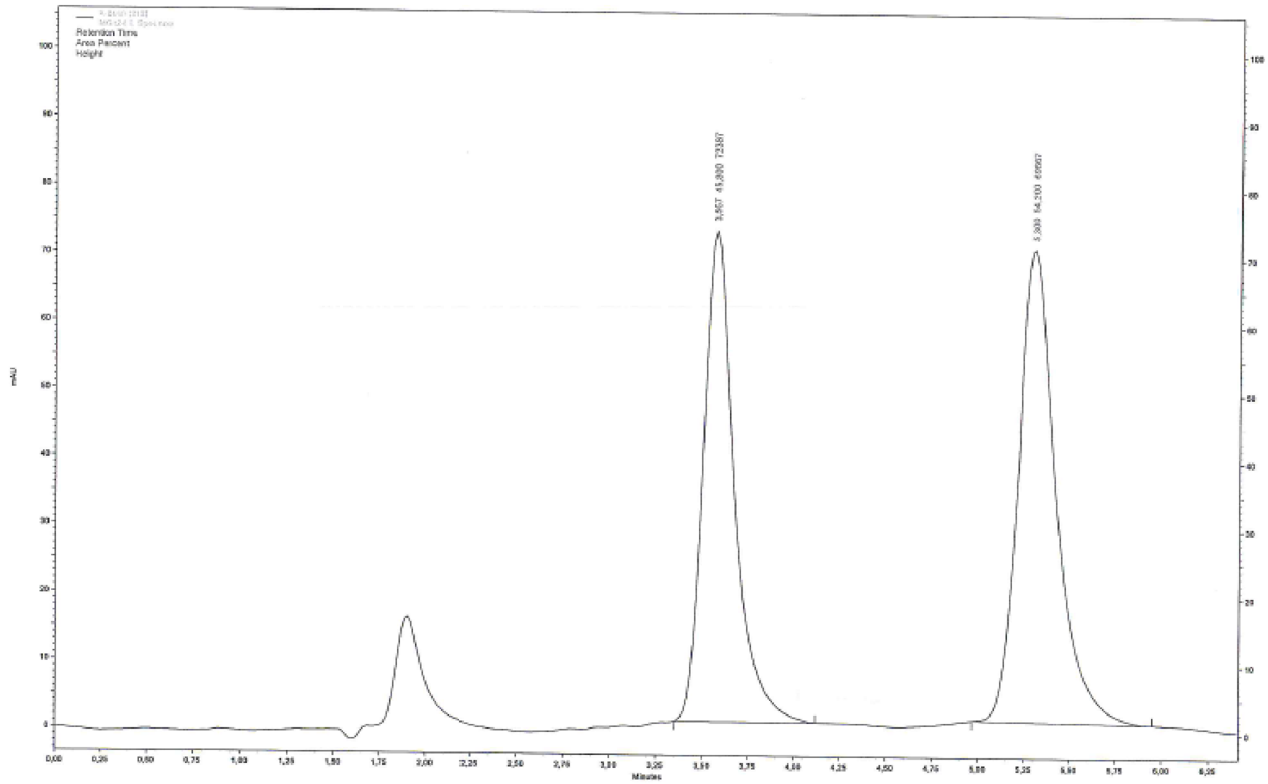

\\Opatz05\EZChrom Elite\Enterprise\Projects\MarioG\Data\MG124 1. Spot neuANA2 Nucleodur100-3 Hex-iProp 80-20 1.0ml-min.met20.06.2013 18-08-42.dat, K-280

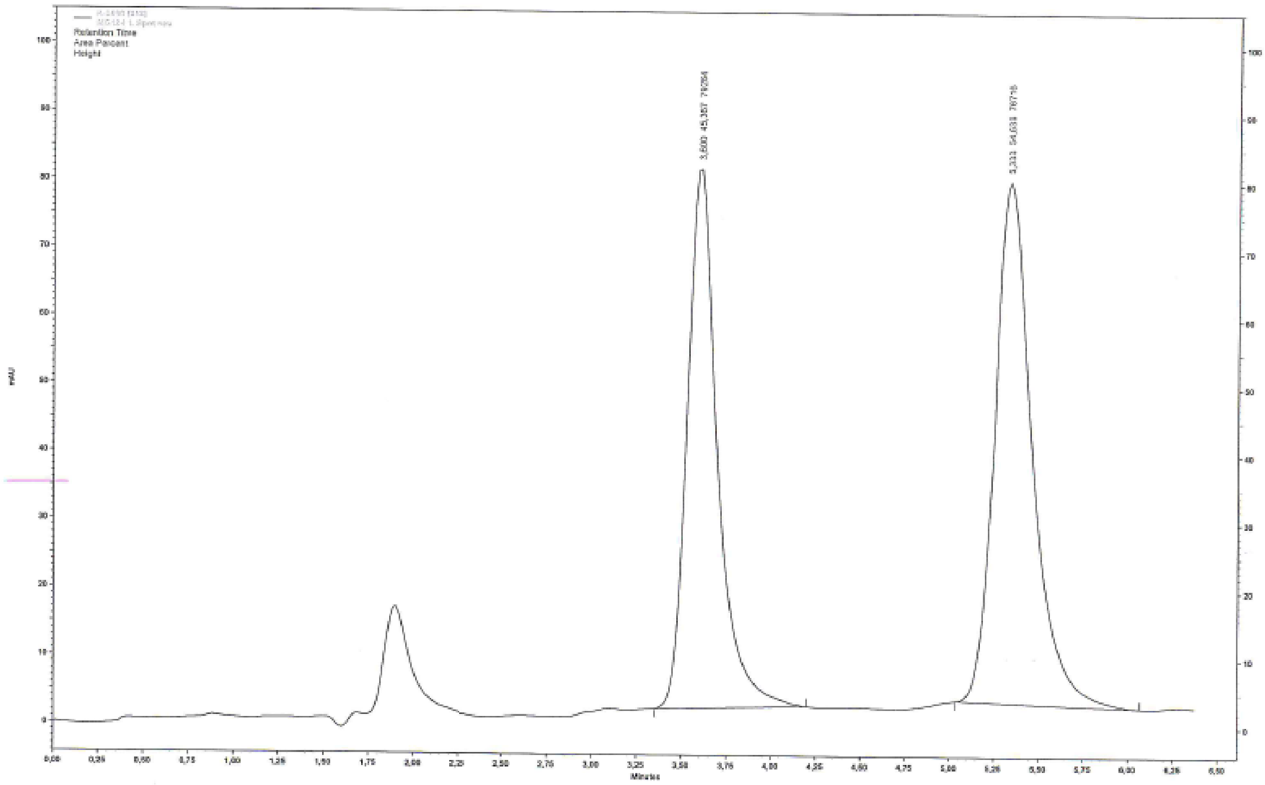

\\Opatz05\EZChrom Elite\Enterprise\Projects\MarioG\Data\MG124 1. Spot neuANA2 Nucleodur100-3 Hex-iProp 80-20 1.0ml-min.met20.06.2013 18-17-35.dat, K-280

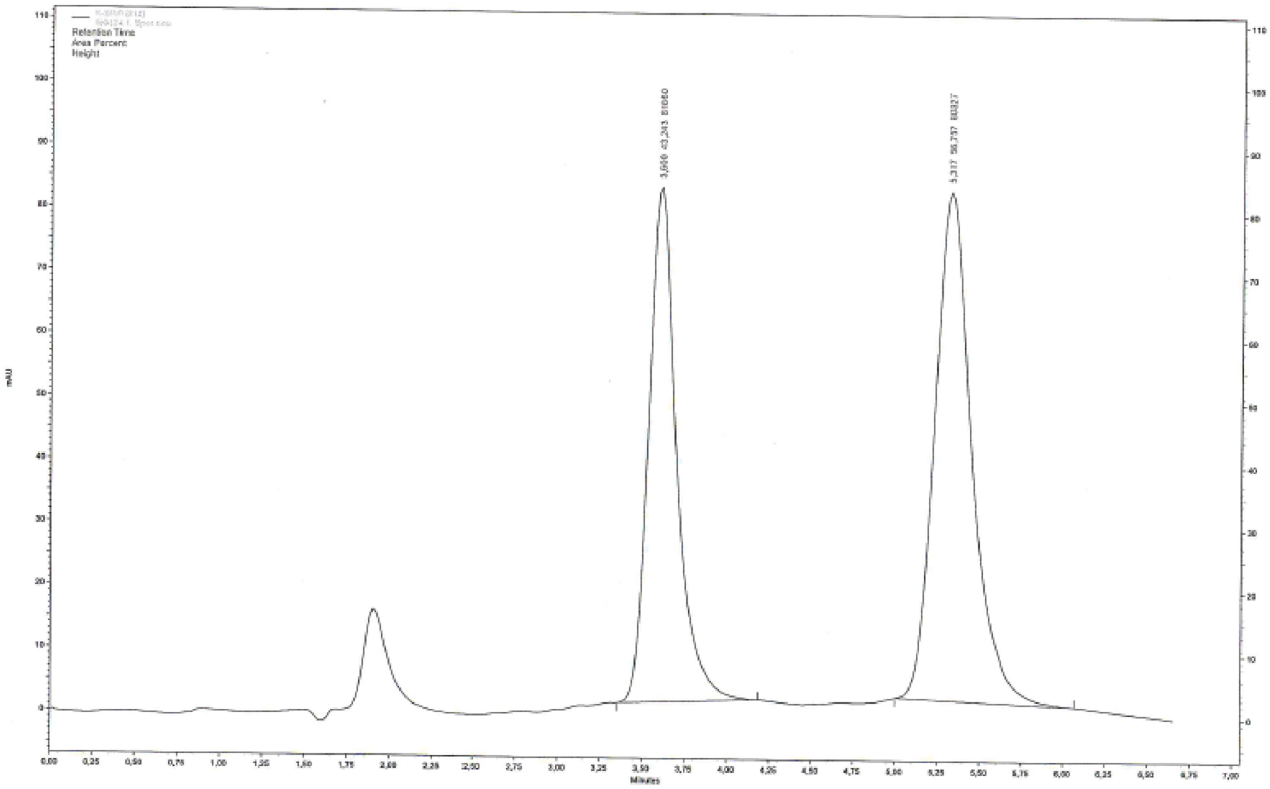

\\Opatz05\EZChrom Elite\Enterprise\Projects\MarioG\Data\MG124 1. Spot neuANA2 Nucleodur100-3 Hex-iProp 80-20 1.0ml-min.met20.06.2013 18-25-39.dat, K-28(

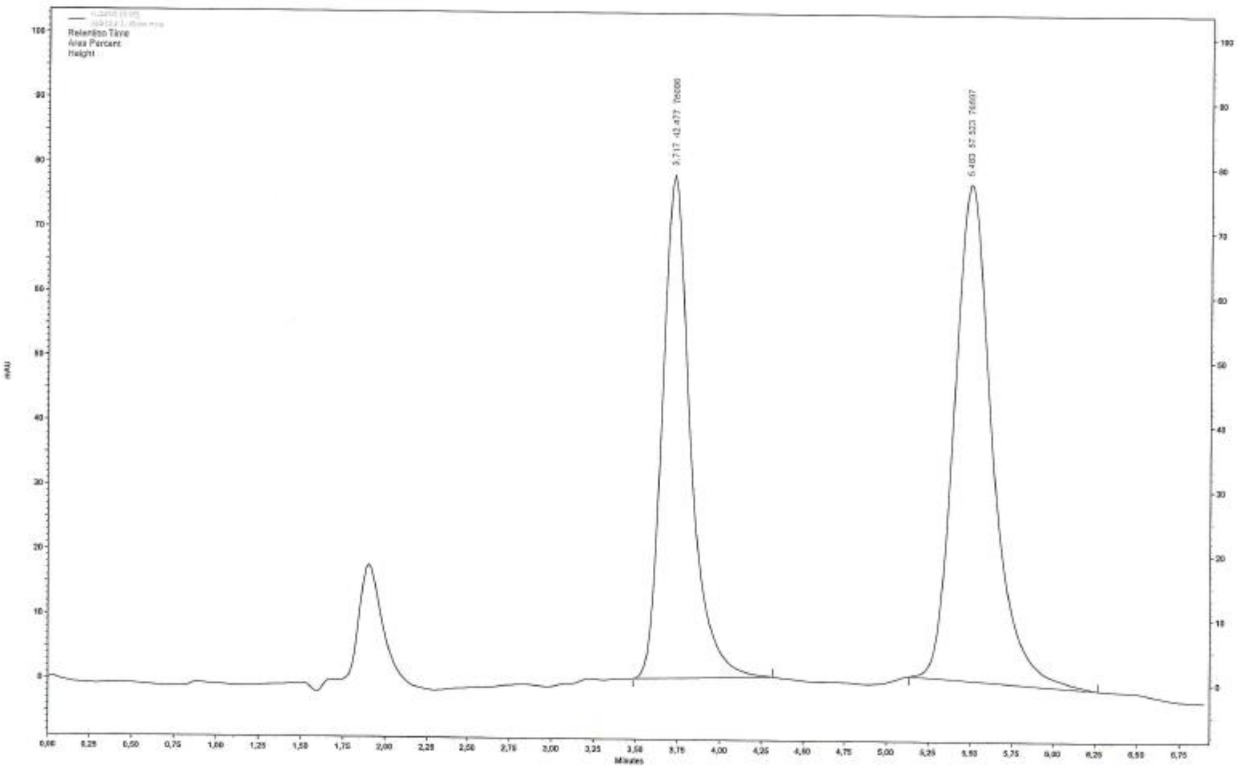

\\Opatz05\EZChrom Elite\Enterprise\Projects\MarioG\Data\MG124 1. Spot neuANA2 Nucleodur100-3 Hex-iProp 80-20 1.0ml-min.met20.06.2013 18-34-26.dat, K-28(

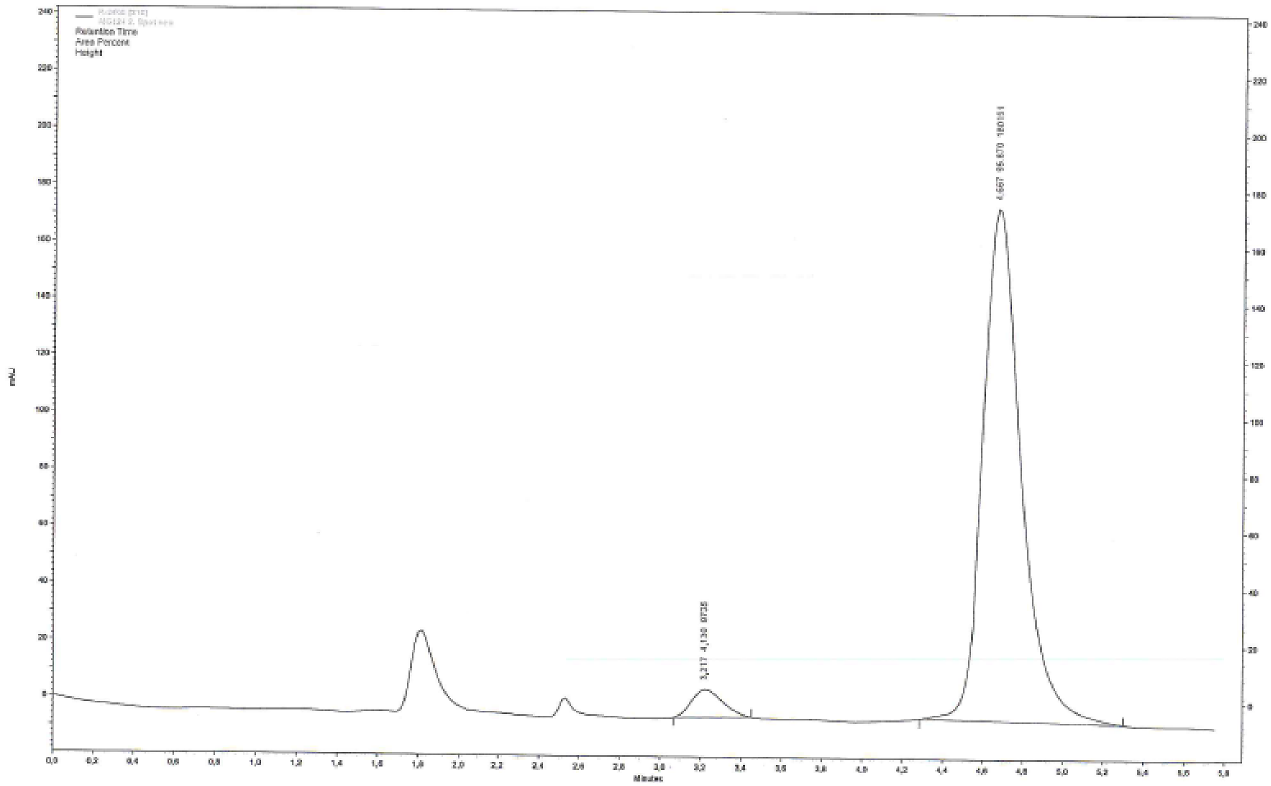

—— \\Opatz05\EZChrom Elite\Enterprise\Projects\MarioG\Data\MG124 2. Spot neuANA2 Nucleodur100-3 Hex-iProp 80-20 1.0ml-min.met19.06.2013 15-41-14.dat, K-280

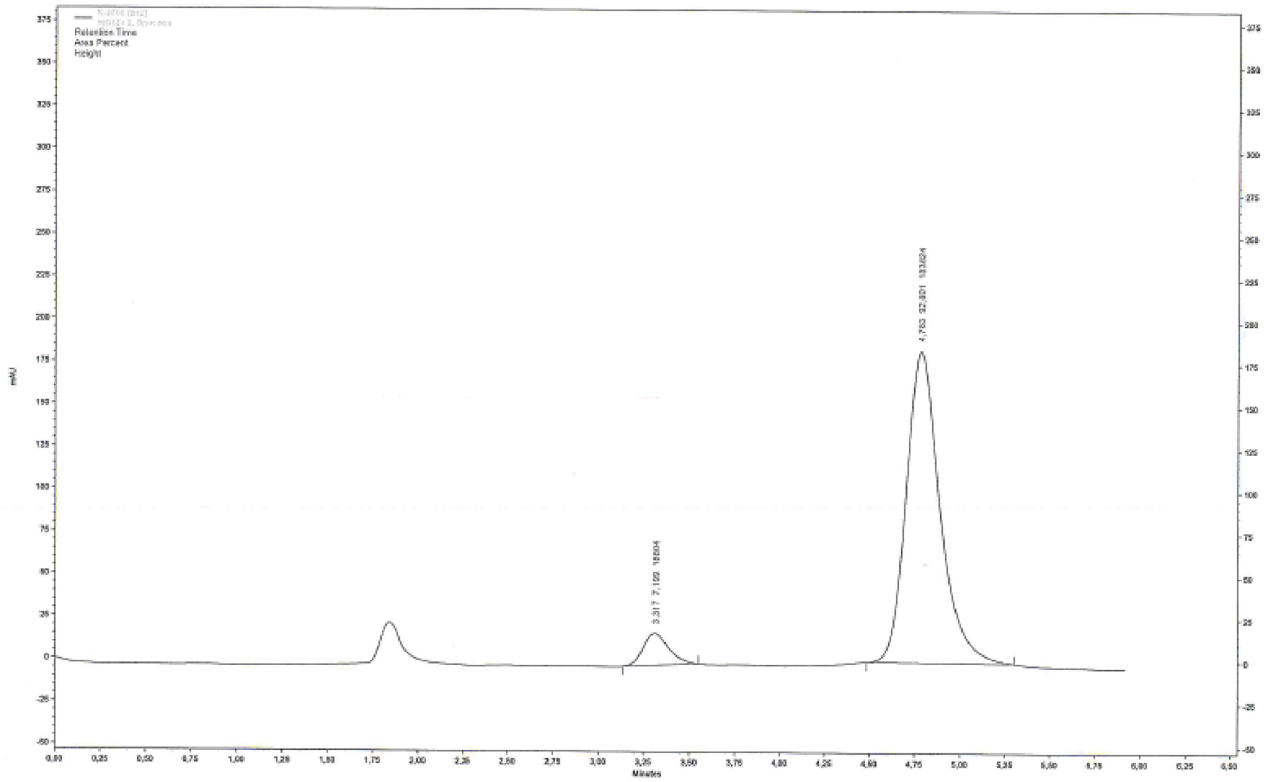

—— \\Opatz05\EZChrom Elite\Enterprise\Projects\MarioG\Data\MG124 2. Spot neuANA2 Nucleodur100-3 Hex-iProp 80-20 1.0ml-min.met19.06.2013 15-49-52.dat, K-280

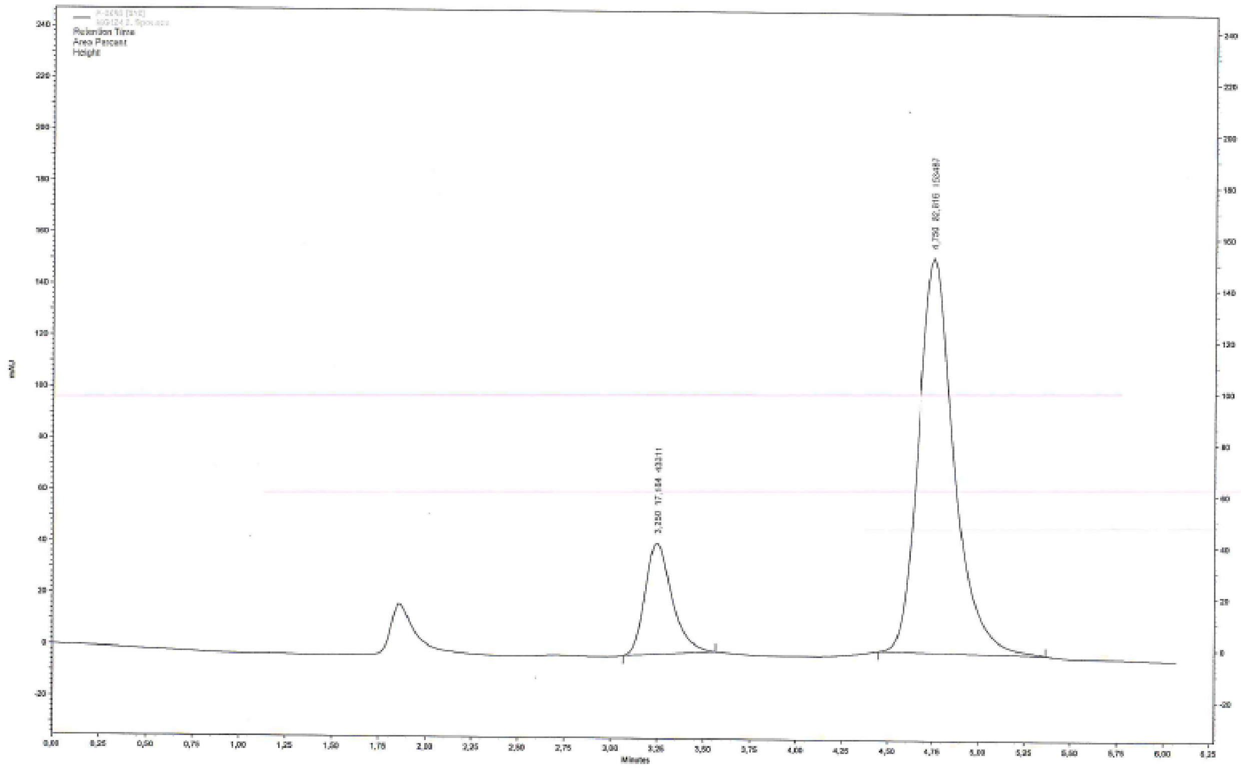

—— \\Opatz05\EZChrom Elite\Enterprise\Projects\MarioG\Data\MG124 2. Spot neuANA2 Nucleodur100-3 Hex-iProp 80-20 1.0ml-min.met19.06.2013 15-56-56.dat, K-280

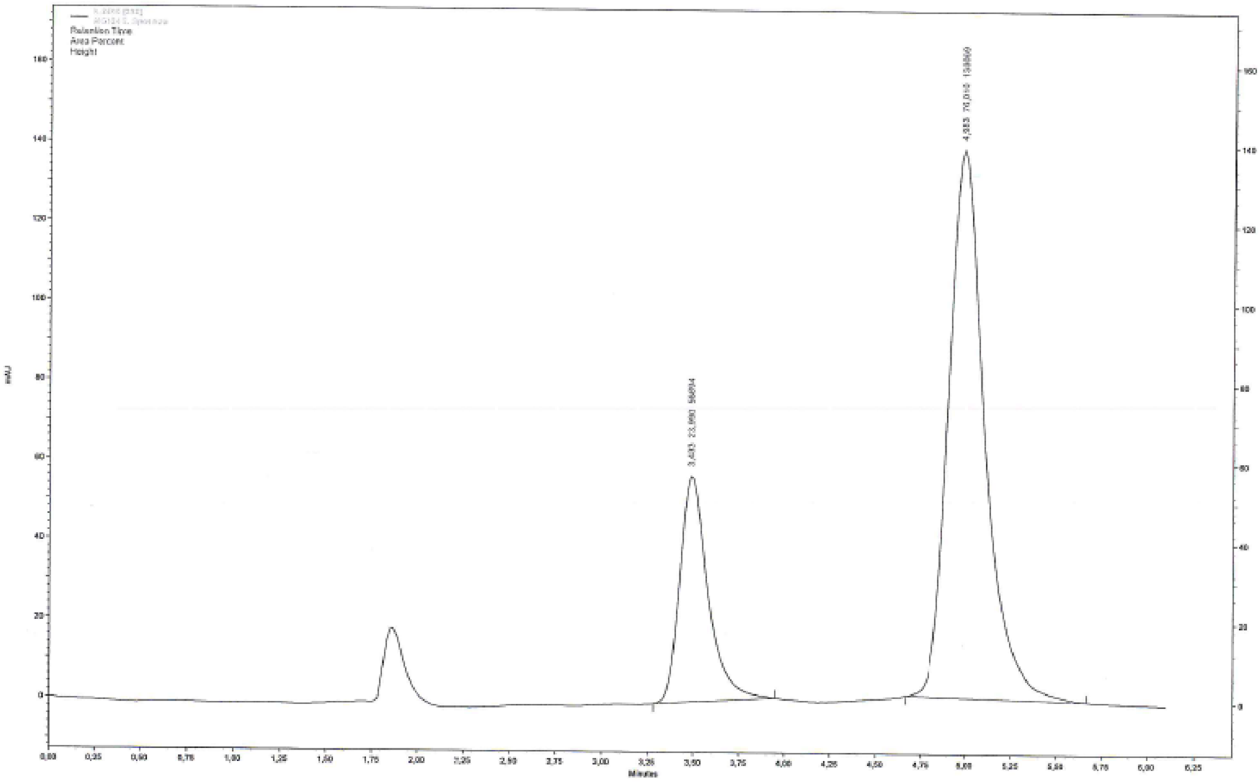

—— \\Opatz05\EZChrom Elite\Enterprise\Projects\MarioG\Data\MG124 2. Spot neuANA2 Nucleodur100-3 Hex-iProp 80-20 1.0ml-min.met19.06.2013 16-04-24.dat, K-28(

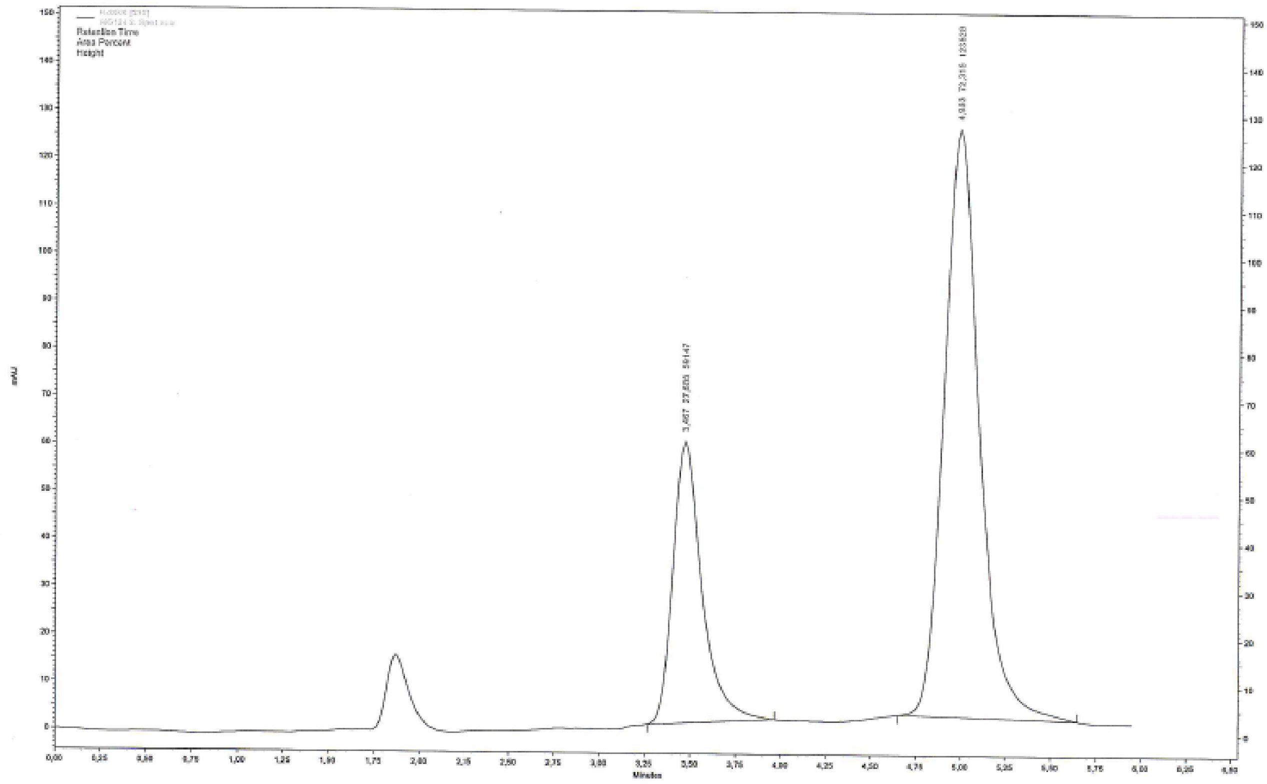

\\Opatz05\EZChrom Elite\Enterprise\Projects\MarioG\Data\MG124 2. Spot neuANA2 Nucleodur100-3 Hex-iProp 80-20 1.0ml-min.met19.06.2013 16-11-57.dat. K-280

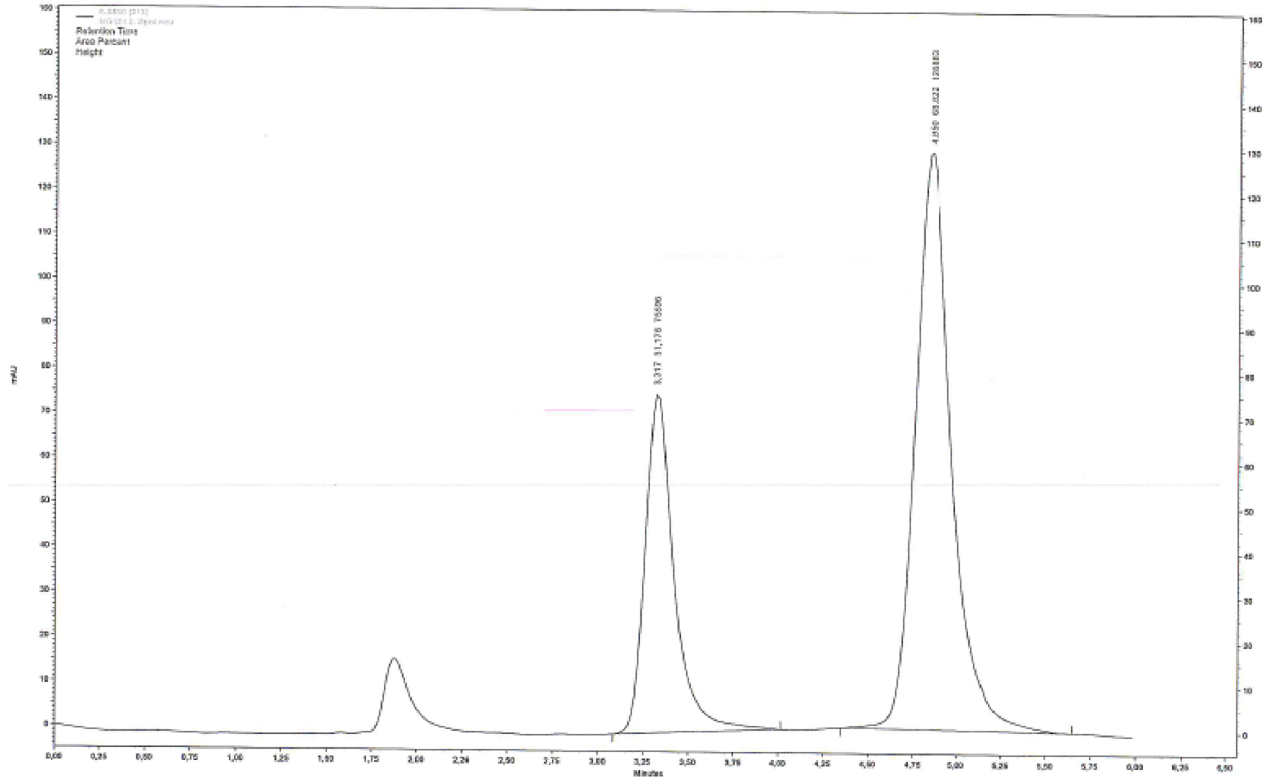

\\Opatz05\EZChrom Elite\Enterprise\Projects\MarioG\Data\MG124 2. Spot neuANA2 Nucleodur100-3 Hex-iProp 80-20 1.0ml-min.met19.06.2013 16-19-26.dat, K-280

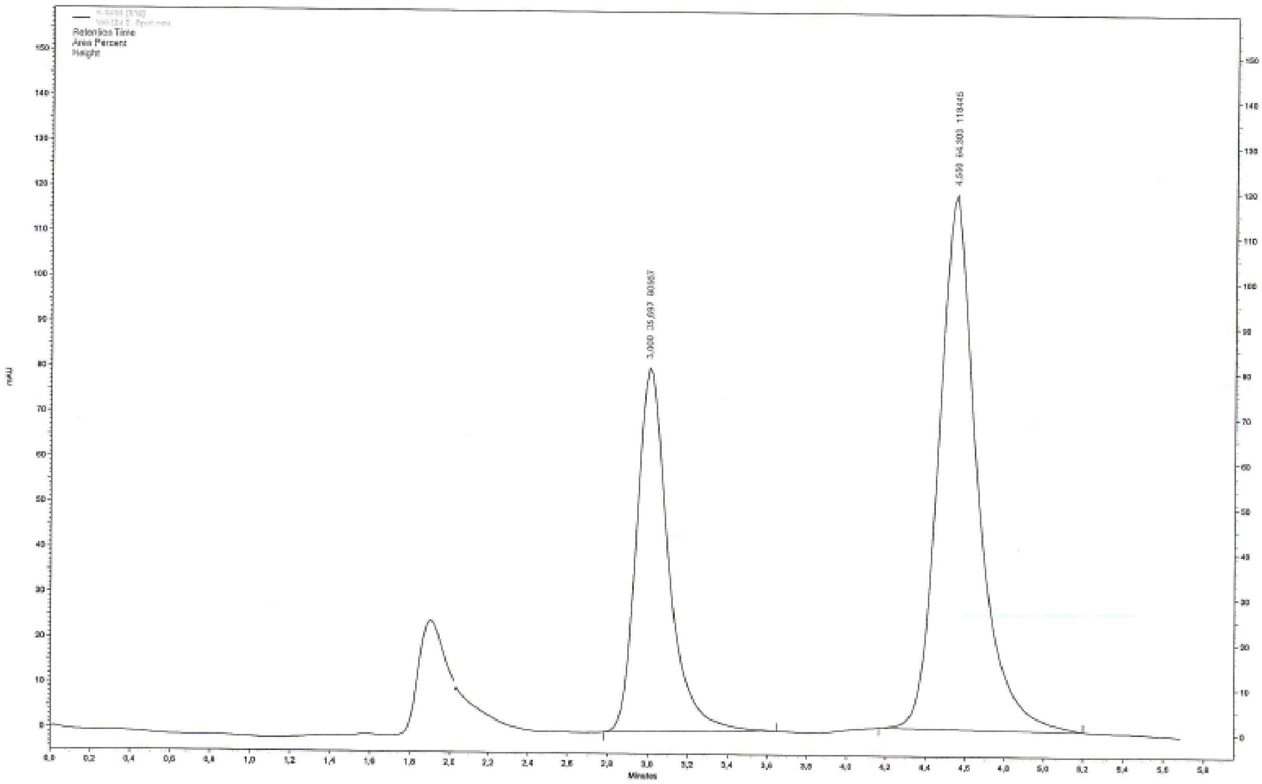

—— \\Opatz05\EZChrom Elite\Enterprise\Projects\MarioG\Data\MG124 2. Spot neuANA2 Nucleodur100-3 Hex-iProp 80-20 1.0ml-min.met19.06.2013 16-26-51.dat, K-280

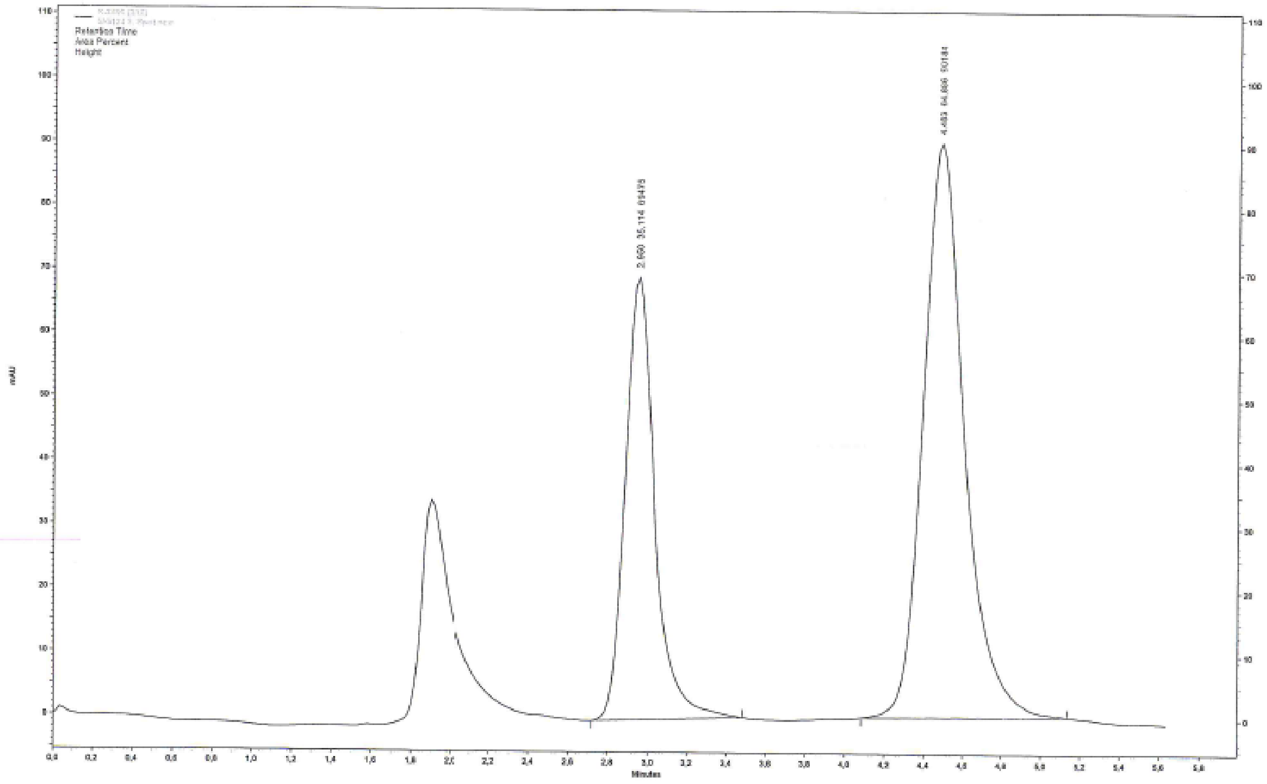

—— \\Opatz05\EZChrom Elite\Enterprise\Projects\MarioG\Data\MG124 2. Spot neuANA2 Nucleodur100-3 Hex-iProp 80-20 1.0ml-min.met19.06.2013 16-33-44.dat, K-280

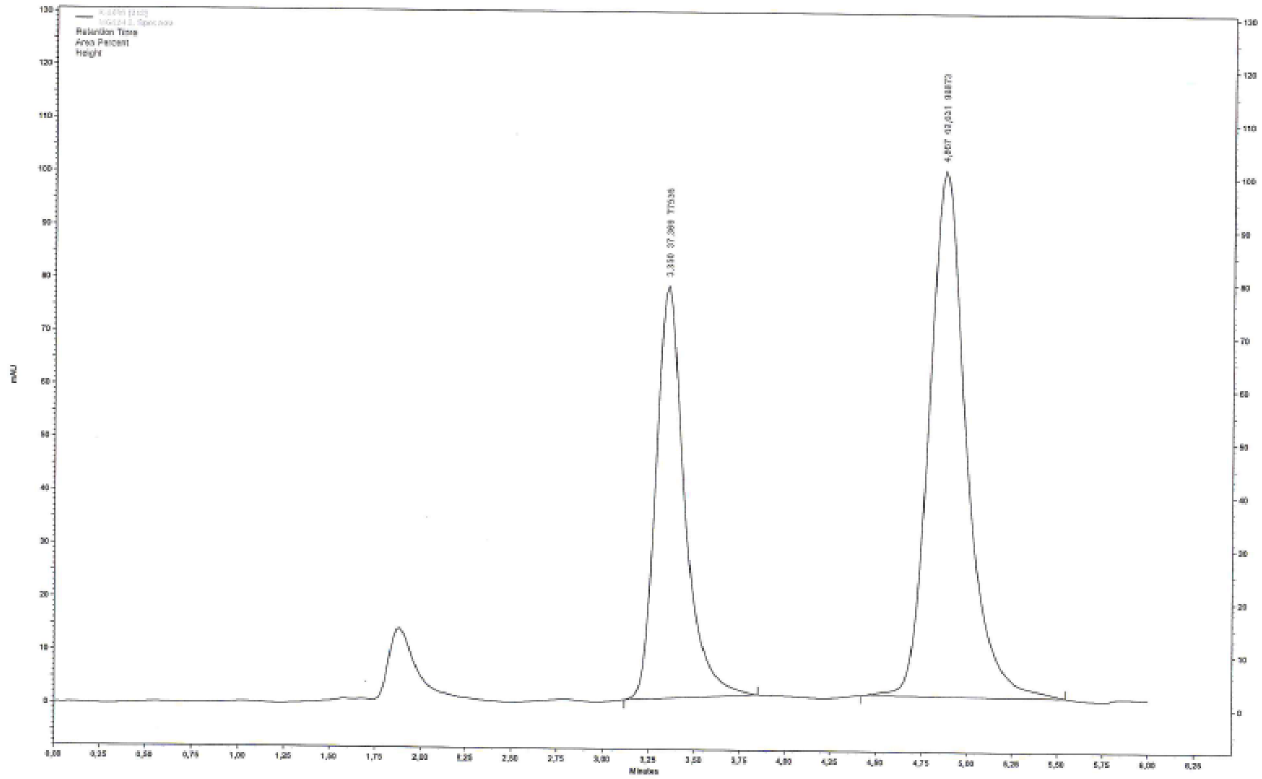

\\Opatz05\EZChrom Elite\Enterprise\Projects\MarioG\Data\MG124 2. Spot neuANA2 Nucleodur100-3 Hex-iProp 80-20 1.0ml-min.met19.06.2013 16-40-38.dat, K-280

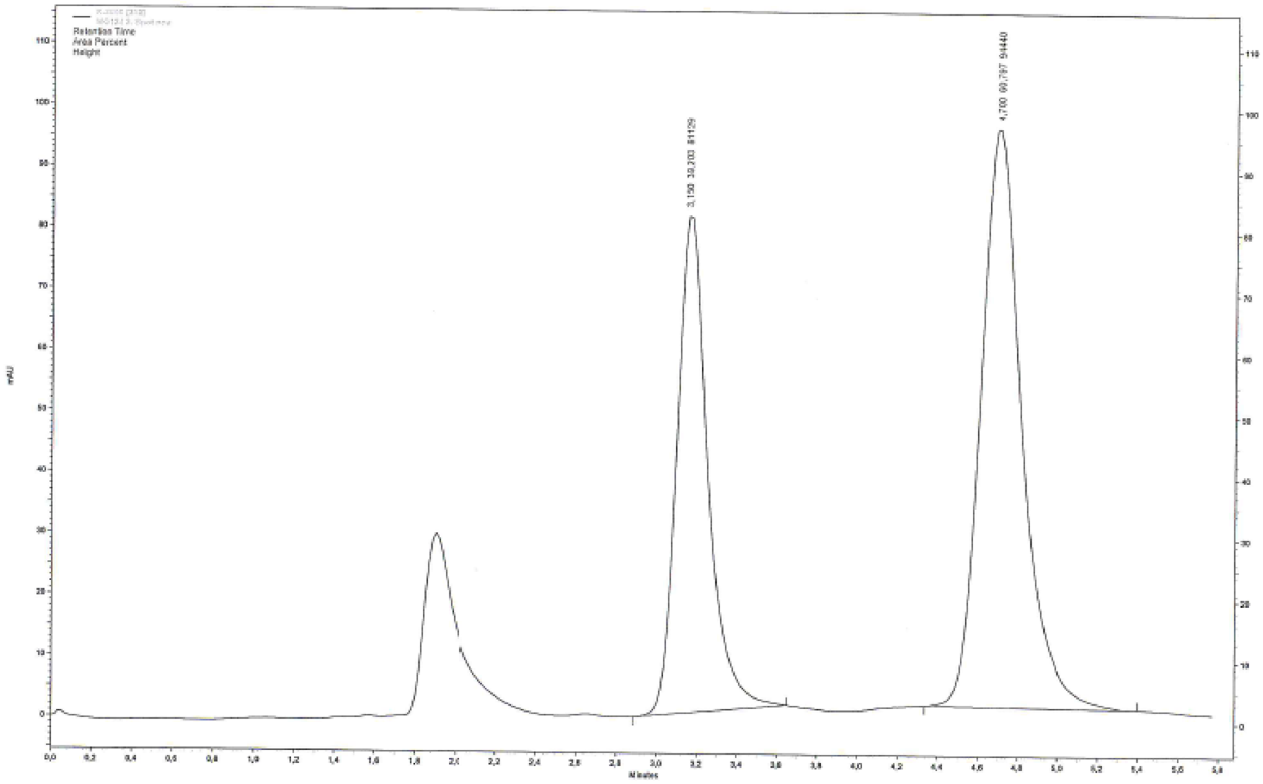

\\Opatz05\EZChrom Elite\Enterprise\Projects\MarioG\Data\MG124 2. Spot neuANA2 Nucleodur100-3 Hex-iProp 80-20 1.0ml-min.met19.06.2013 16-47-52.dat, K-280

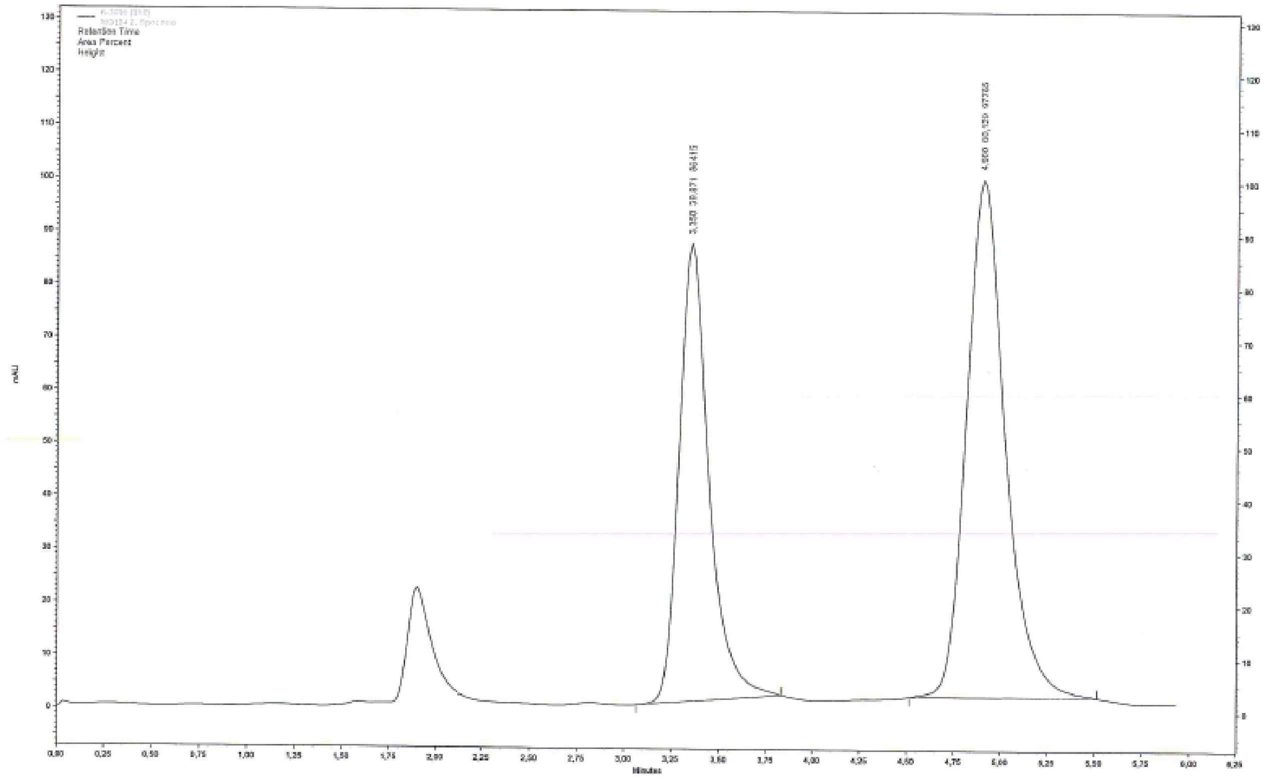

\\Opatz05\EZChrom Elite\Enterprise\Projects\MarioG\Data\MG124 2. Spot neuANA2 Nucleodur100-3 Hex-iProp 80-20 1.0ml-min.met19.06.2013 16-54-55.dat, K-280

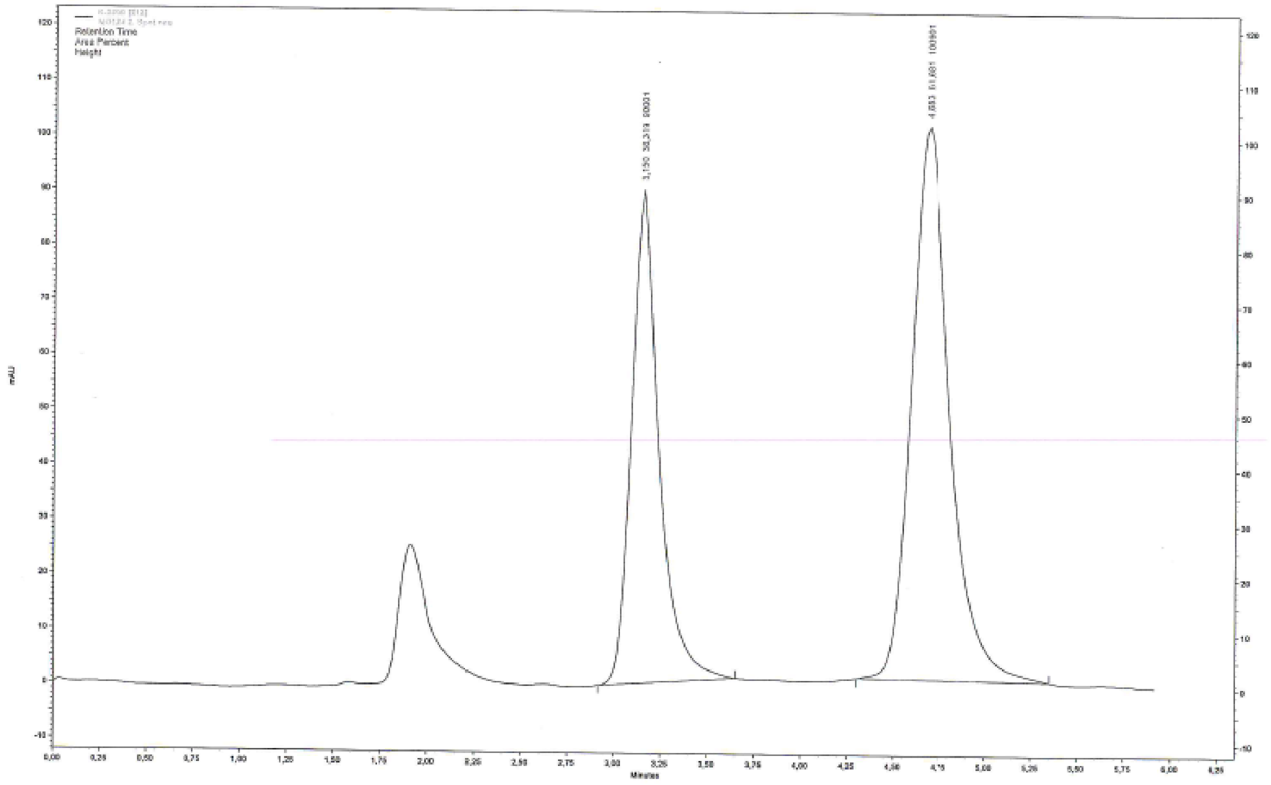

\\Opatz05\EZChrom Elite\Enterprise\Projects\MarioG\Data\MG124 2. Spot neuANA2 Nucleodur100-3 Hex-iProp 80-20 1.0ml-min.met19.06.2013 17-02-04.dat, K-280

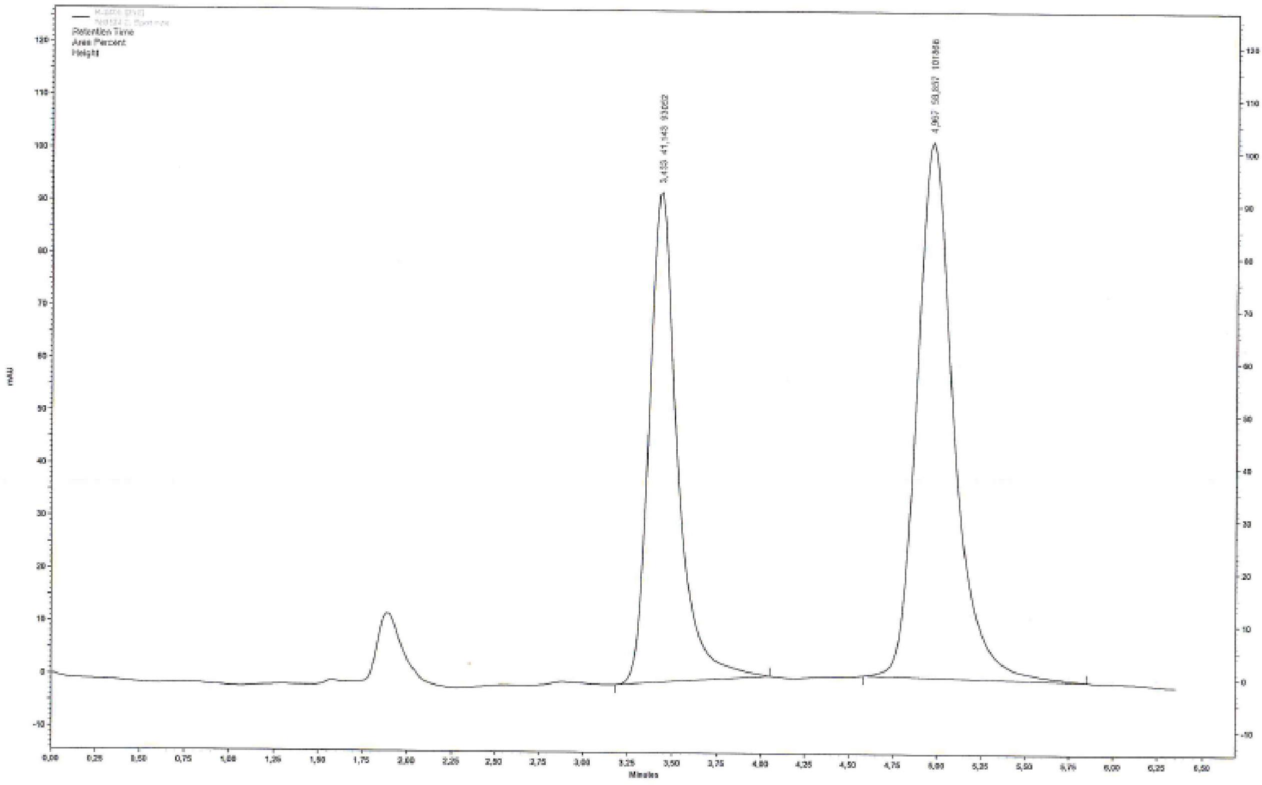

—— \\Opatz05\EZChrom Elite\Enterprise\Projects\MarioG\Data\MG124 2. Spot neuANA2 Nucleodur100-3 Hex-iProp 80-20 1.0ml-min.met19.06.2013 17-09-41.dat, K-280

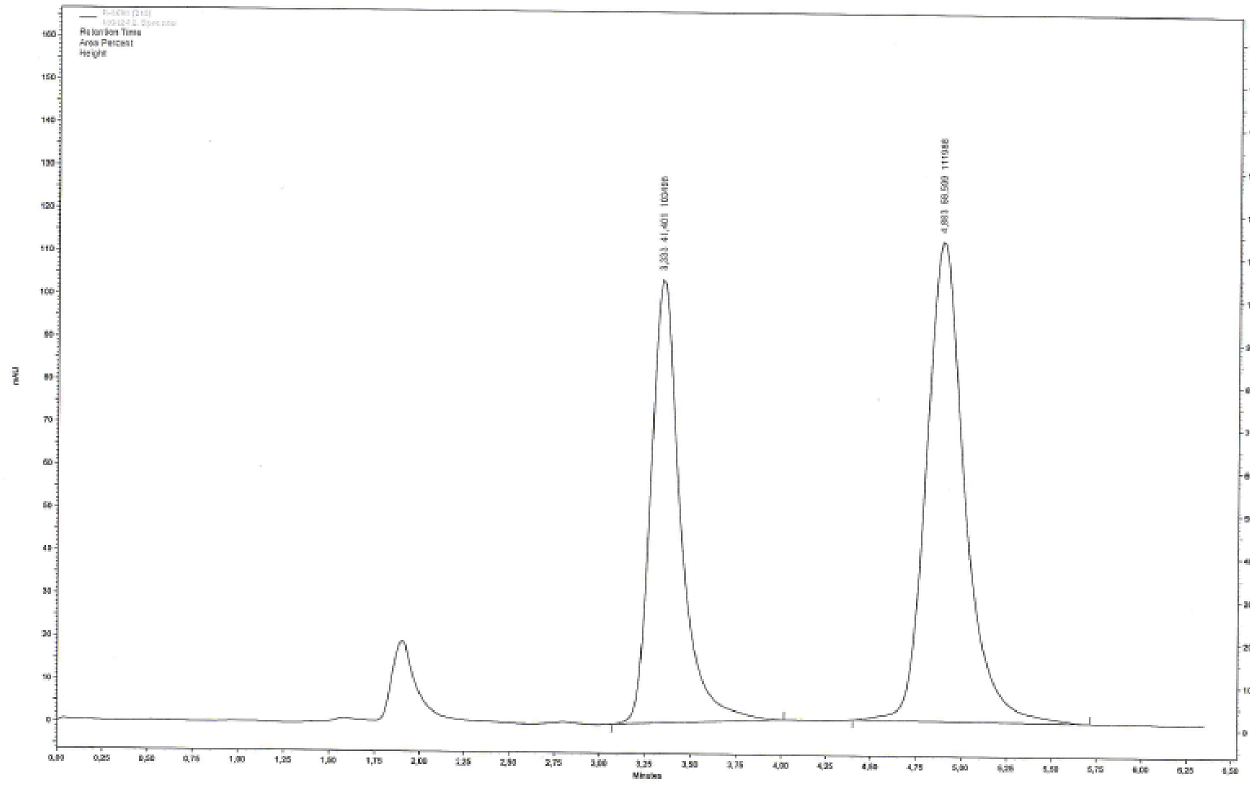

—— \\Opatz05\EZChrom Elite\Enterprise\Projects\MarioG\Data\MG124 2. Spot neuANA2 Nucleodur100-3 Hex-iProp 80-20 1.0ml-min.met19.06.2013 17-17-24.dat, K-280

4. DHPLC Measurements

The DHPLC measurements were performed using the same conditions described for the kinetic studies (vide supra).

#	T [°C]	t_R^1 [min]	t_R^2 [min]	w_h^1 [s]	w_h^2 [s]	h_p	Area A_1	A_2	α	*Z* → *E* k_1 [10^{-4} 1/s]	ΔG‡ [kJ/mol]	*E* → Z k_1 [10^{-4} 1/s]	ΔG‡ [kJ/mol]
**1**	20.0	3.42	4.92	8	14	1.6%	42.38	57.62	1.62				
**2**	20.0	3.30	4.82	8	14	1.2%	46.13	53.87	1.66				
**3**	20.0	3.40	4.95	10	14	1.8%	40.58	59.42	1.65				
**4**	25.0	2.90	4.67	12	12	2.4%	40.73	59.27	1.93				
**5**	25.0	3.23	4.62	10	12	1.6%	41.83	58.17	1.62				
**6**	25.0	3.33	4.73	8	14	1.8%	40.72	59.28	1.60				
**7**	30.0	2.93	4.17	8	12	3.0%	41.89	58.11	1.64	5.85	93.05	4.22	93.88
**8**	30.0	3.03	4.25	8	12	3.2%	42.3	57.7	1.60	5.93	93.02	4.35	93.80
**9**	30.0	3.08	4.32	8	12	2.7%	41.95	58.05	1.59	5.06	93.42	3.65	94.24
**10**	35.0	2.88	3.97	8	10	5.3%	44.11	55.89	1.58	10.6	93.10	8.38	93.71
**11**	35.0	3.05	4.12	8	10	5.4%	41.67	58.33	1.52	10.4	93.16	7.40	94.03
**12**	35.0	2.92	3.97	10	12	5.3%	40.71	59.29	1.55	9.05	93.51	6.21	94.47
**13**	40.0	2.82	3.75	8	8	9.4%	42.77	57.23	1.51	19.8	93.04	14.8	93.80
**14**	40.0	2.90	3.83	8	10	9.9%	41.11	58.89	1.49	17.1	93.41	12.0	94.35
**15**	40.0	2.72	3.65	8	10	9.2%	40.52	59.48	1.54	17.1	93.41	11.7	94.41
**16**	45.0	2.65	3.47	8	10	15.5%	41.4	58.6	1.50	25.1	93.94	17.7	94.86

**17**	45.0	2.73	3.57	8	10	17.6%	41.83	58.17	1.48	27.6	93.68	19.8	94.55
**18**	45.0	2.57	3.35	8	10	15.5%	41.6	58.4	1.50	24.8	93.97	17.6	94.87
**19**	50.0	2.70	3.45	8	10	28.9%	39.04	60.96	1.44	41.4	94.10	26.5	95.30
**20**	50.0	2.73	3.48	8	10	30.6%	40.26	59.74	1.43	42.3	94.05	28.5	95.11
**21**	50.0	2.68	3.45	6	10	30.4%	38.63	61.37	1.46	44.8	93.89	28.2	95.14
**22**	55.0	2.63	3.30	10	10	52.1%	37.94	62.06	1.41	67.4	94.27	41.2	95.62
**23**	55.0	2.55	3.20	8	10	54.7%	38.77	61.23	1.42	70.4	94.16	44.6	95.40
**24**	55.0	2.53	3.17	10	10	55.3%	38.36	61.64	1.41	71.4	94.12	44.4	95.41

5. NMR-Spectra

1H 432/1
AK opatz / Name Geffe / Code MG120 / Probennr. 852
h1.bbfo CDCl3 {C:\Bruker\av2-400\automation\400JNC4} tag 32

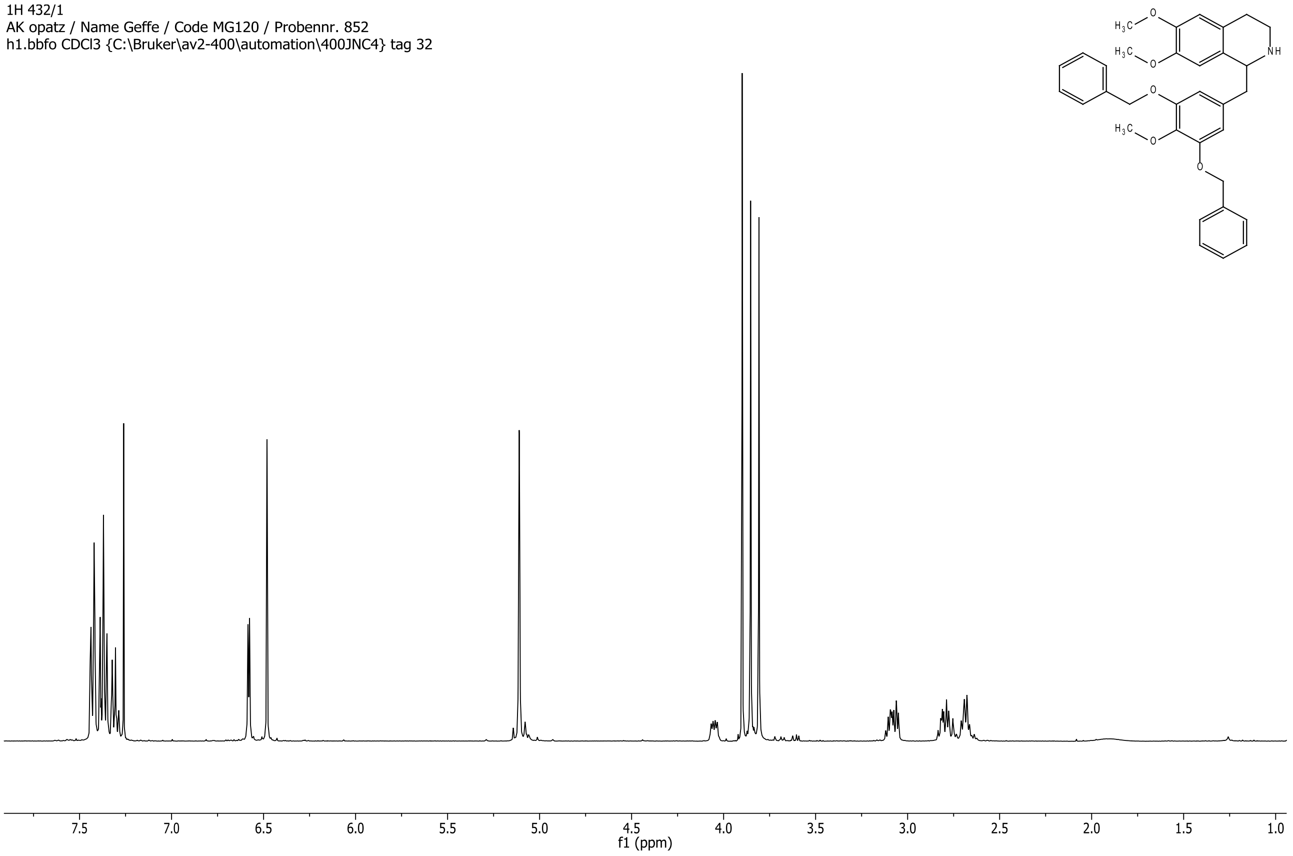

152/1
AK opatz / Name Geffe / Code MG120 / Probennr. N37
c13_30min.bbfo CDCl3 {C:\Bruker\av2-400\automation\400JNC4} nacht 52

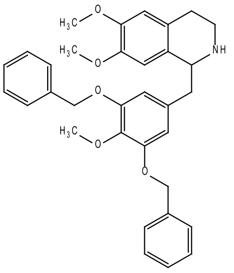

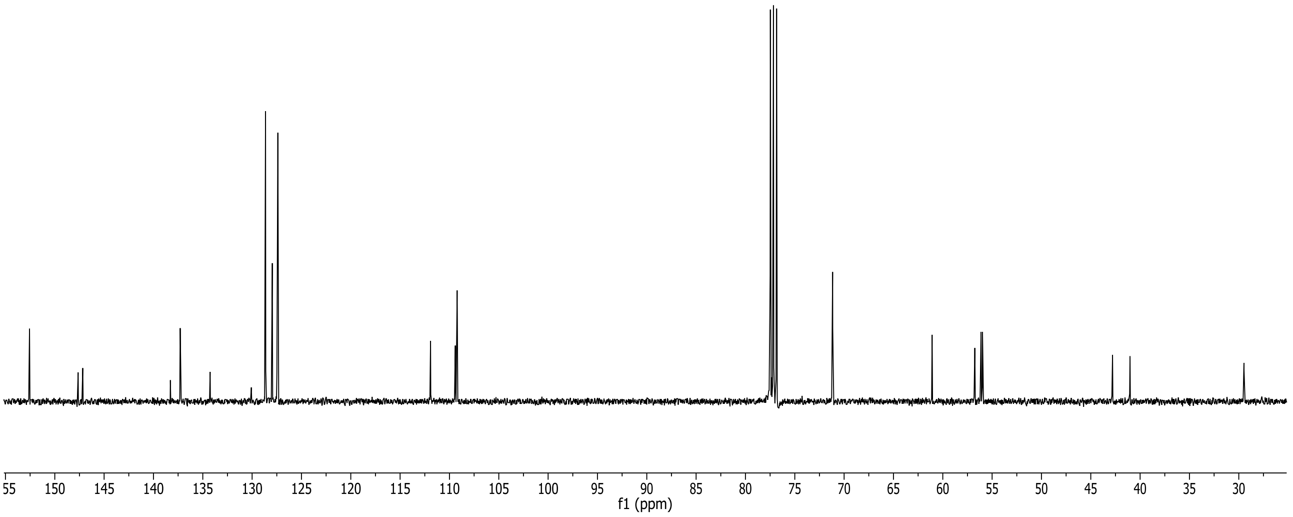

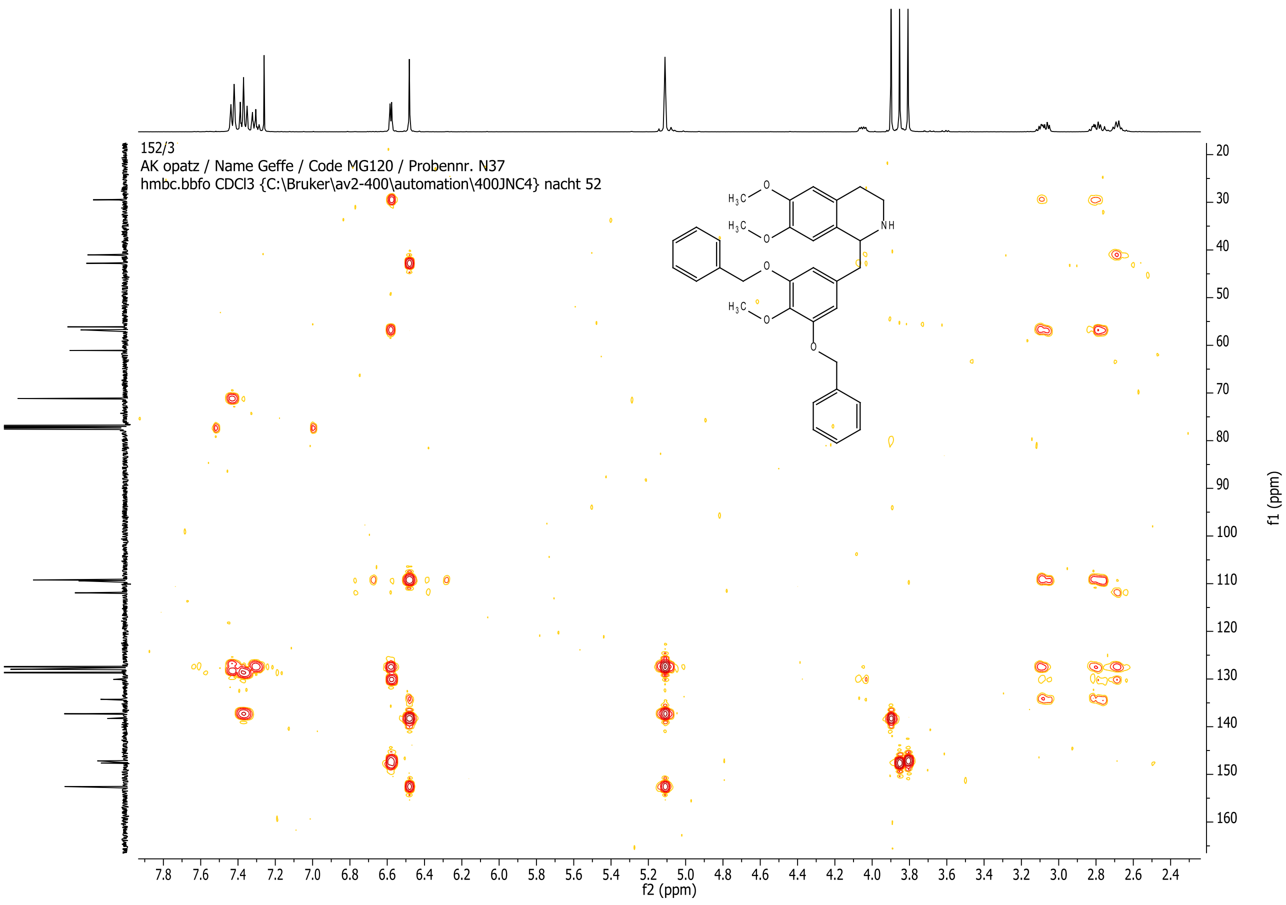
152/3
AK opatz / Name Geffe / Code MG120 / Probennr. N37
hmbc.bbfo CDCl3 {C:\Bruker\av2-400\automation\400JNC4} nacht 52
f2 (ppm)
f1 (ppm)

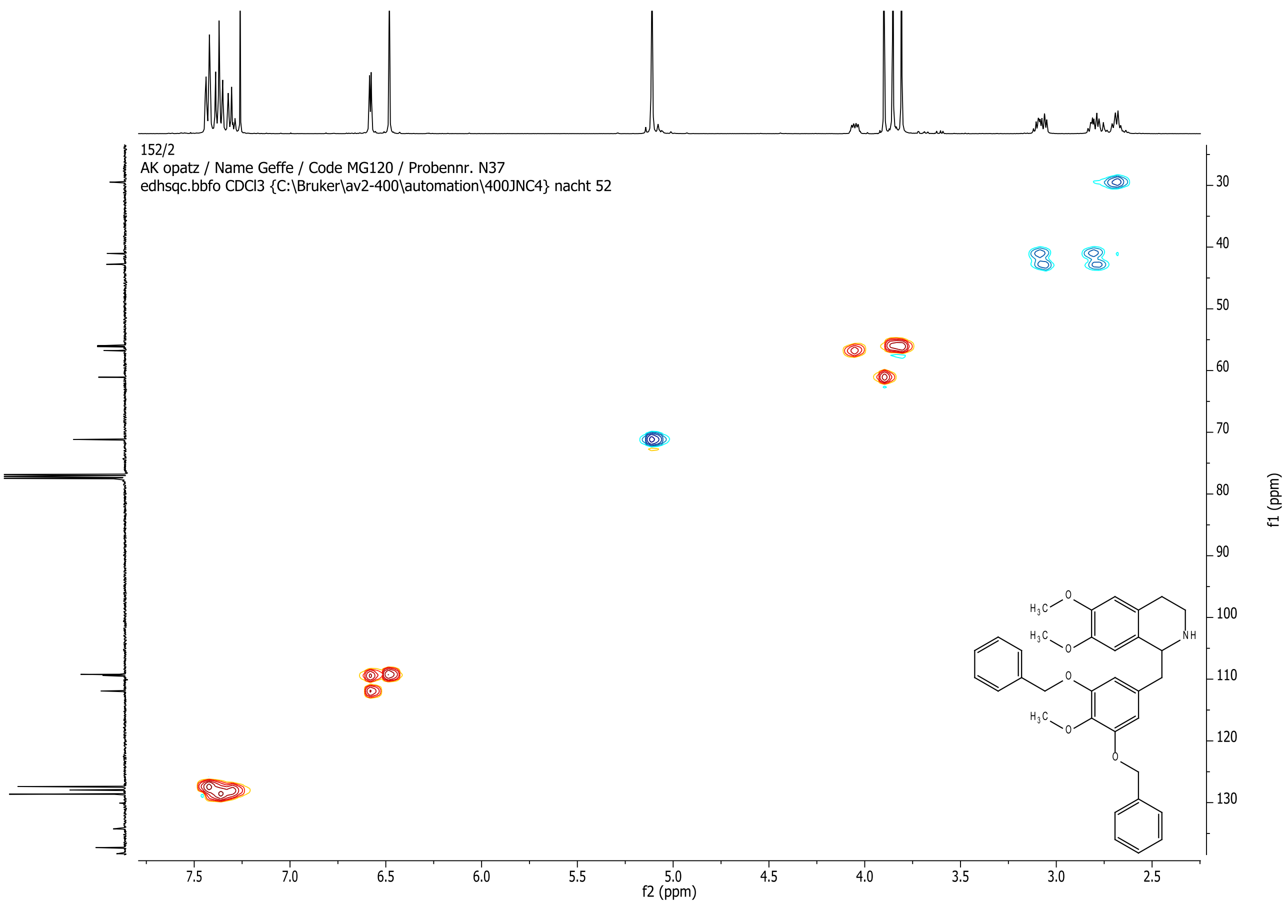
152/2
AK opatz / Name Geffe / Code MG120 / Probennr. N37
edhsqc.bbfo CDCl3 {C:\Bruker\av2-400\automation\400JNC4} nacht 52
f2 (ppm)
f1 (ppm)

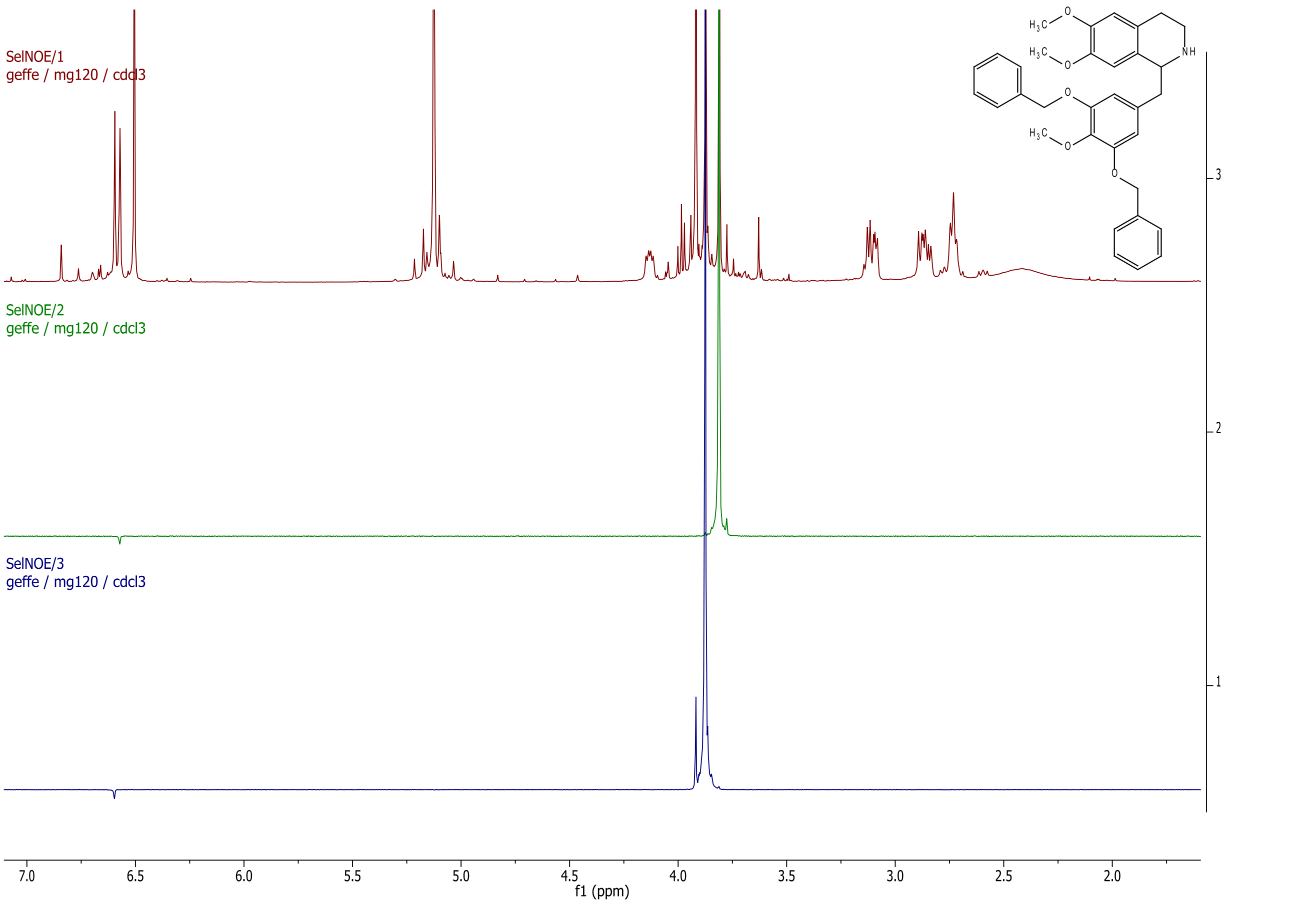
SelNOE/1
geffe / mg120 / cdcl3
SelNOE/2
geffe / mg120 / cdcl3
SelNOE/3
geffe / mg120 / cdcl3
f1 (ppm)

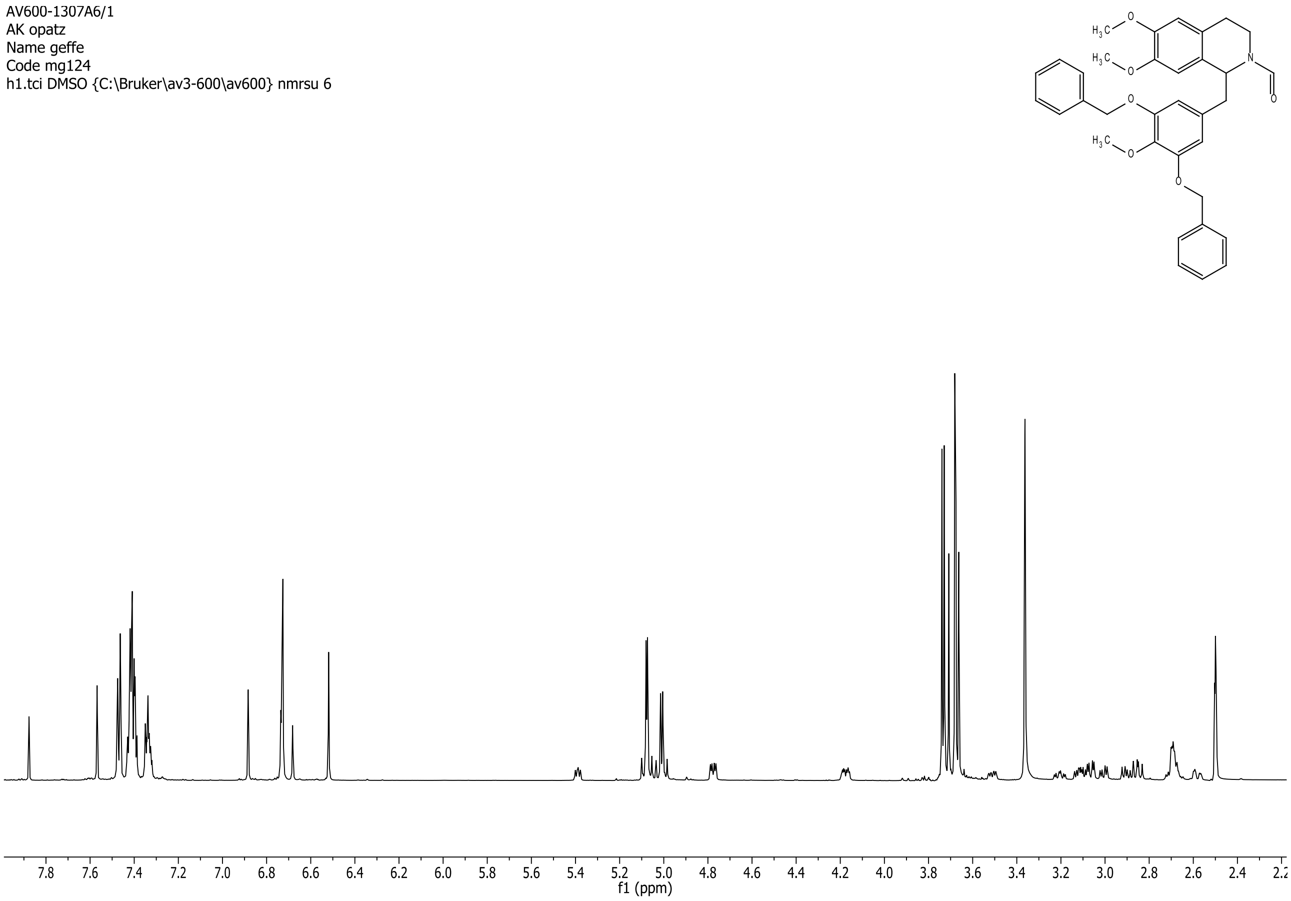
AV600-1307A6/1
AK opatz
Name geffe
Code mg124
h1.tci DMSO {C:\Bruker\av3-600\av600} nmrsu 6
f1 (ppm)

AV600-1307A6/2
AK opatz
Name geffe
Code mg124
c13.tci DMSO {C:\Bruker\av3-600\av600} nmrsu 6

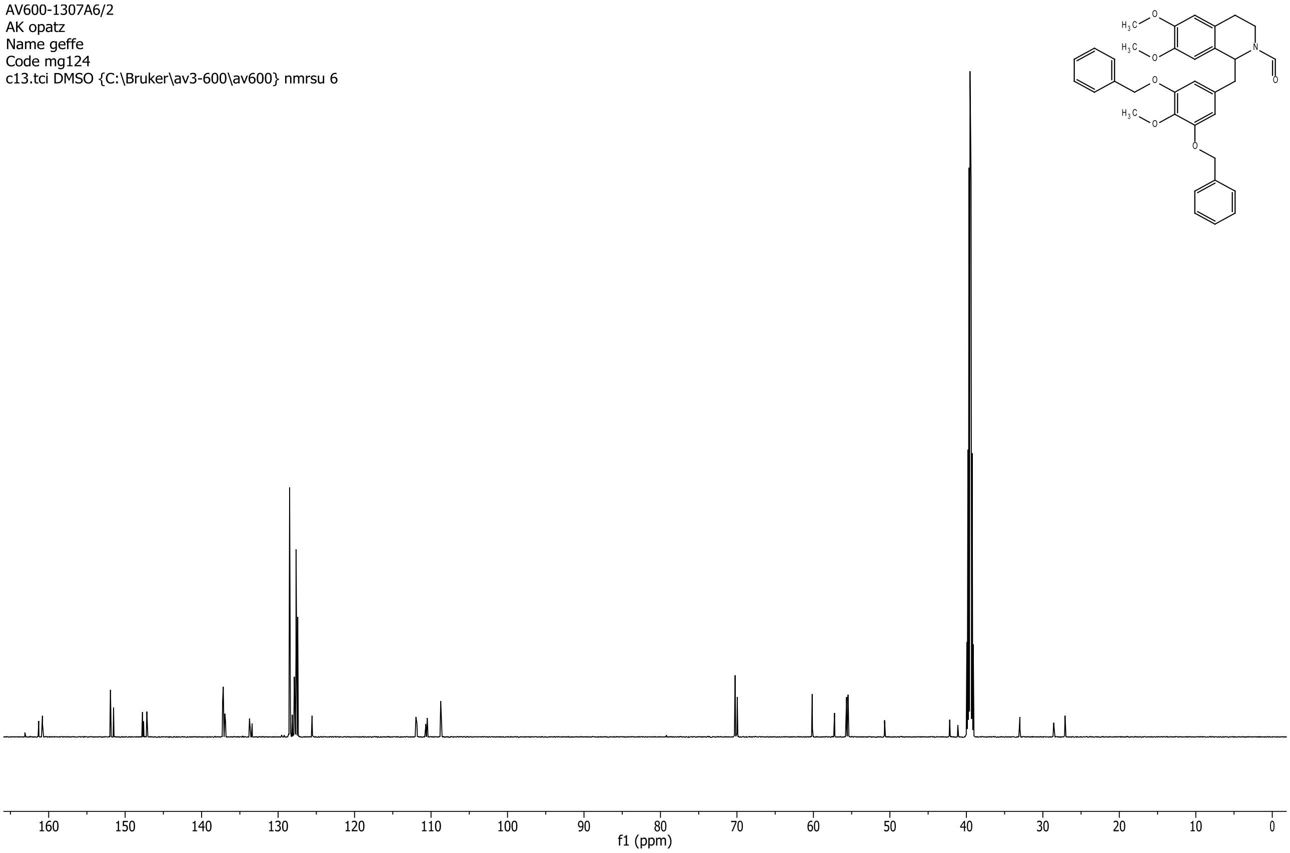

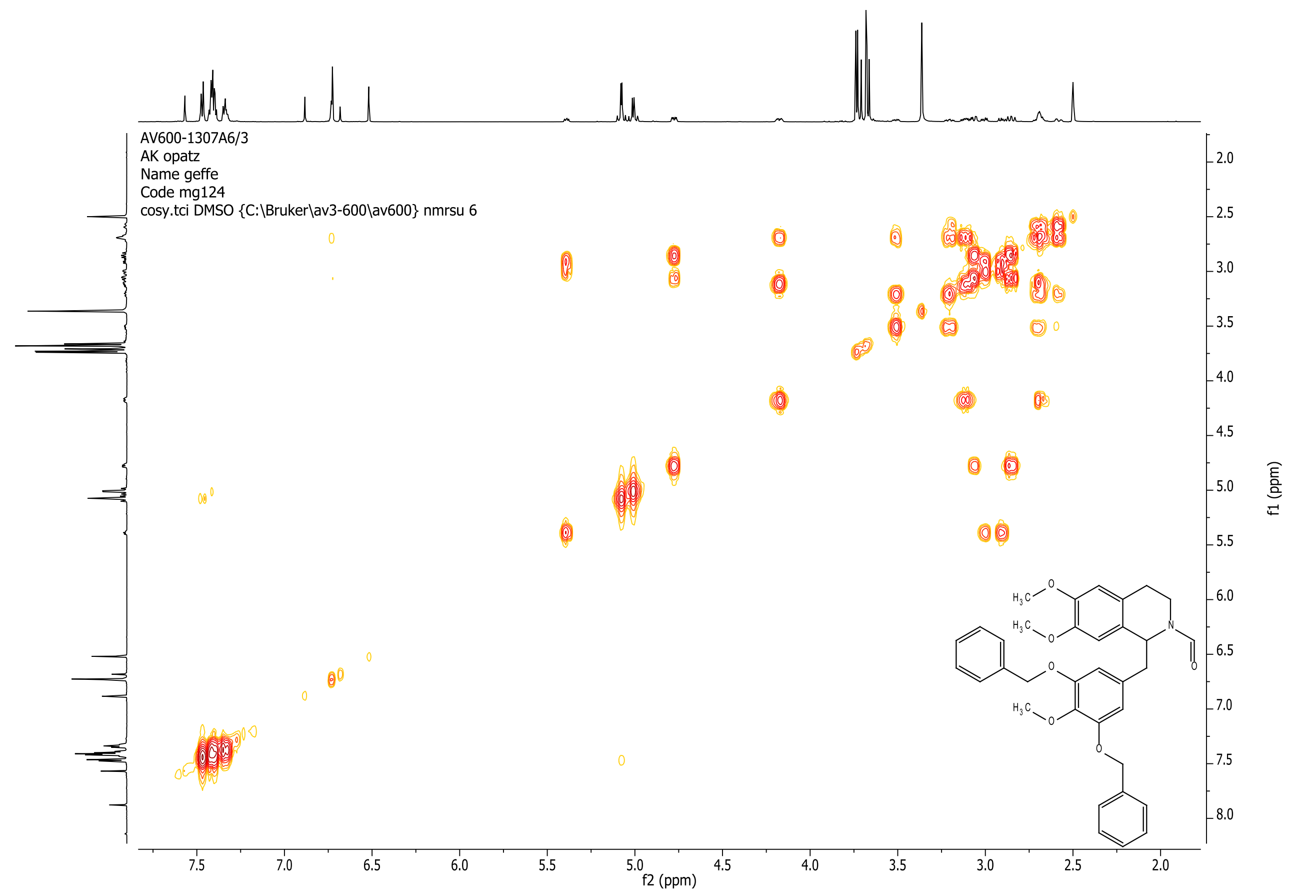
AV600-1307A6/3
AK opatz
Name geffe
Code mg124
cosy.tci DMSO {C:\Bruker\av3-600\av600} nmrsu 6
f2 (ppm)
f1 (ppm)
H3C
O
N
O

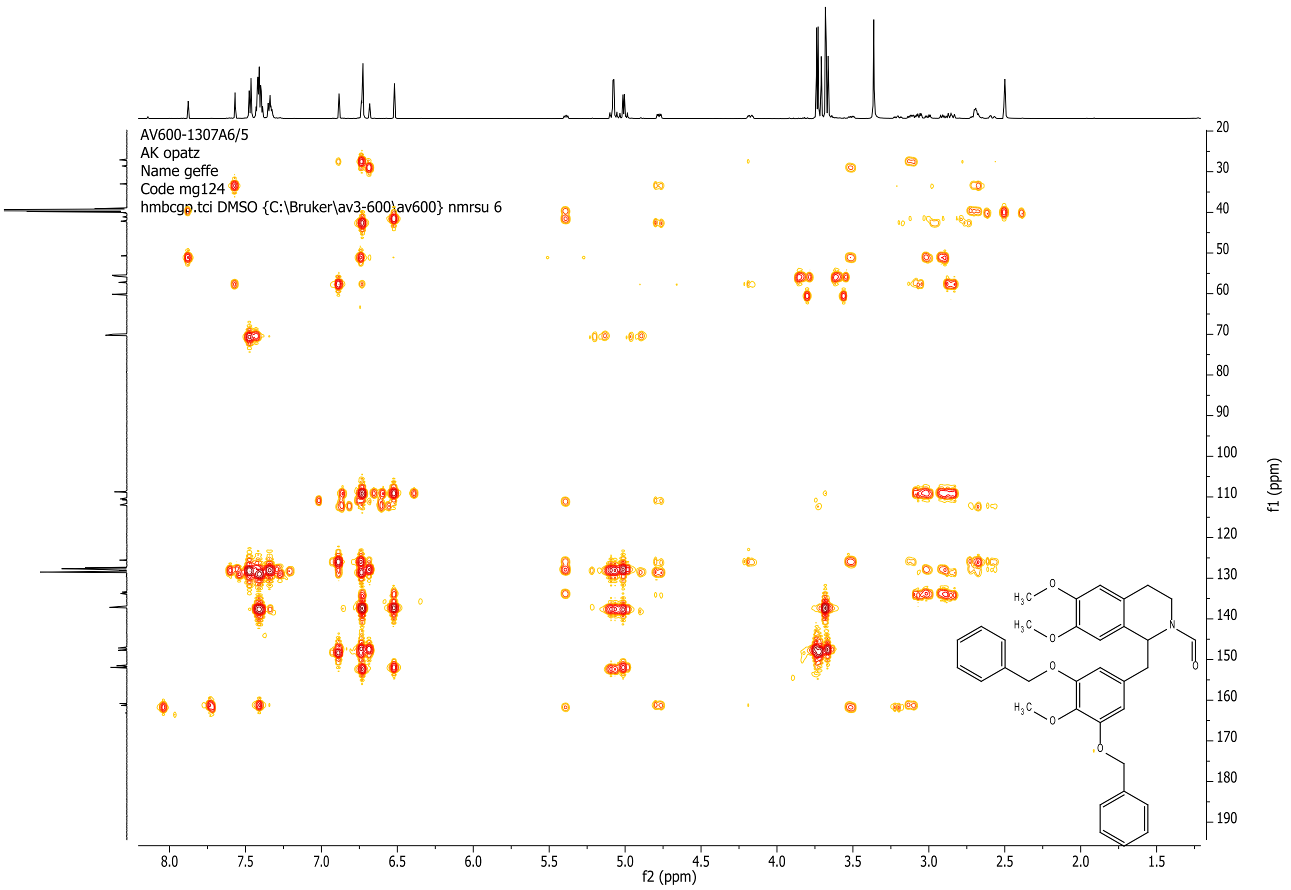
AV600-1307A6/5
AK opatz
Name geffe
Code mg124
hmbcgp.tci DMSO {C:\Bruker\av3-600\av600} nmrsu 6
f2 (ppm)
f1 (ppm)
H3C
O
N
O

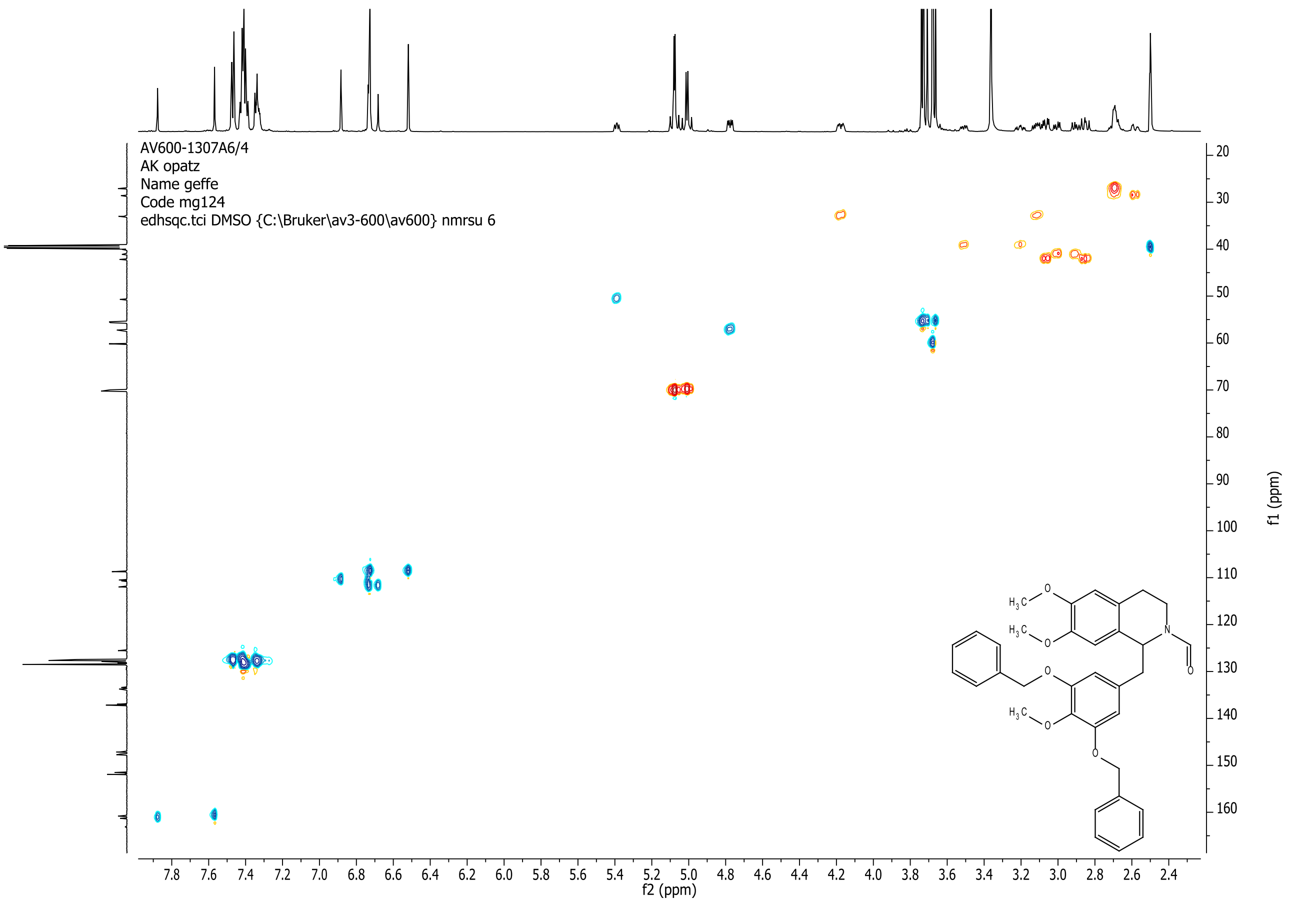

AV600-1307A6/4
AK opatz
Name geffe
Code mg124
edhsqc.tci DMSO {C:\Bruker\av3-600\av600} nmrsu 6
f2 (ppm)
f1 (ppm)

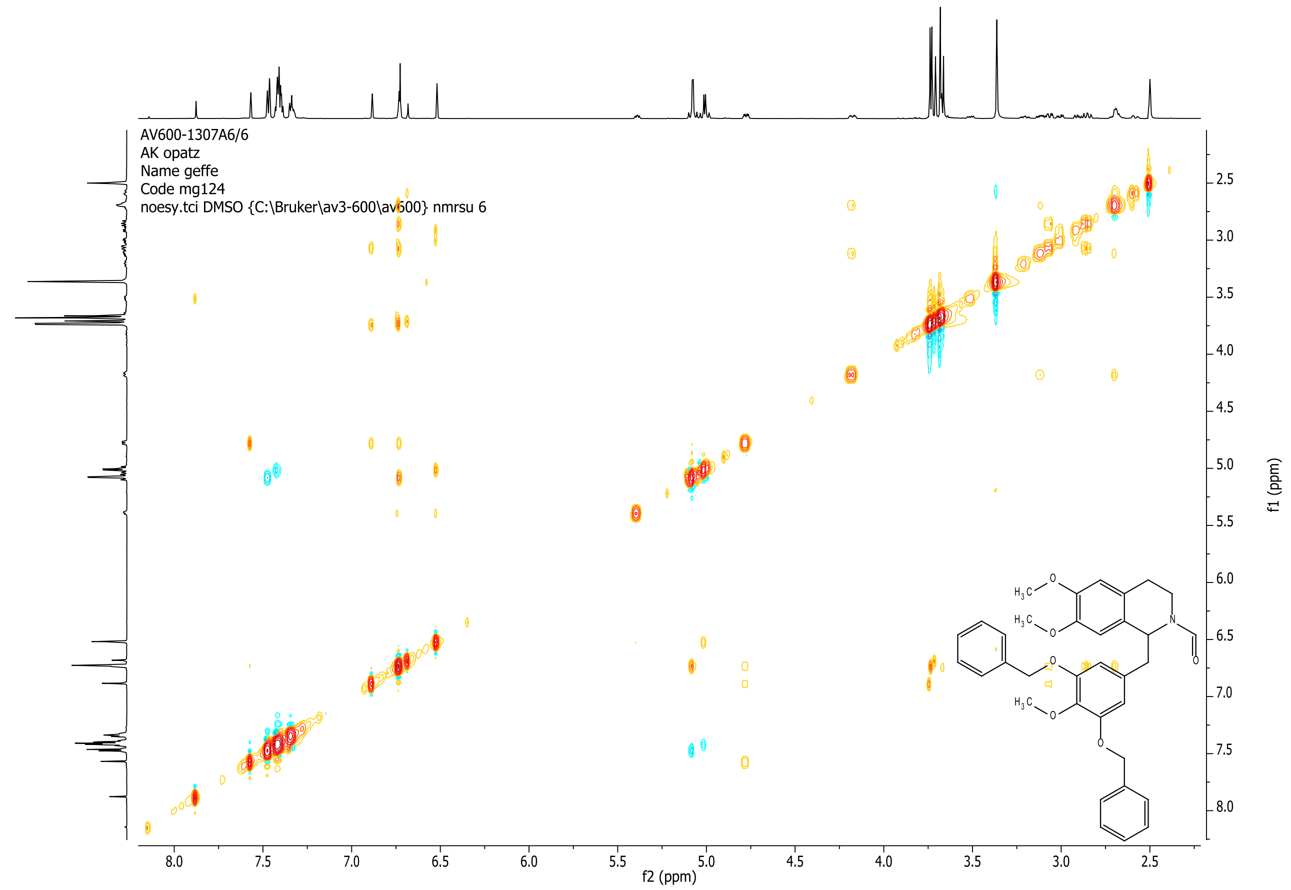

AV600-1307A6/6
AK opatz
Name geffe
Code mg124
noesy.tci DMSO {C:\Bruker\av3-600\av600} nmrsu 6
f2 (ppm)
f1 (ppm)

6. Results of DFT calculations

Final calculation for *E*-**4** in hexane:

! BP RIJCOSX def2-SVP def2-SVP/J VDW10 TightOpt tightSCF COSMO(Hexane) Numfreq

%maxcore 2000

%pal nprocs 8

end

C	0.469254	-2.355421	0.532285
C	0.856291	-2.232380	-0.814461
C	-0.159910	-2.148205	-1.948956
N	-1.514232	-2.462292	-1.487546
C	-1.932933	-2.030576	-0.152523
C	-0.980143	-2.580967	0.906641
C	1.460425	-2.285296	1.540840
C	2.814310	-2.099599	1.233232
C	3.211936	-2.013857	-0.137992
C	2.227815	-2.080079	-1.131796
O	3.818618	-1.958571	2.146736
O	4.546261	-1.855306	-0.358672
C	4.978006	-1.622005	-1.692435
C	3.475716	-1.962683	3.526470

C	-0.111505	-0.761468	-2.685585
C	-0.540351	0.388744	-1.806291
C	0.303405	0.822602	-0.760282
C	-0.160227	1.754590	0.181353
C	-1.461856	2.313818	0.058507
C	-2.292799	1.897327	-1.007339
C	-1.829220	0.931398	-1.923255
O	-3.551439	2.401511	-1.211589
C	-4.487011	2.383185	-0.100317
O	-1.919728	3.223416	0.986021
C	-1.358325	4.535707	0.866801
O	0.566426	2.198680	1.255058
C	1.805980	1.556181	1.553515
C	2.953495	1.934874	0.632752
C	4.123543	1.148537	0.626565
C	5.205155	1.495107	-0.199621
C	5.127674	2.624322	-1.034499
C	3.959891	3.407871	-1.034212
C	2.879558	3.066180	-0.202373
C	-2.395953	-3.120139	-2.303265
O	-3.563503	-3.392084	-2.032059
H	0.106640	-2.914589	-2.710386
H	-1.971676	-0.923145	-0.100350

H	-2.963093	-2.399621	-0.011287
H	-1.208970	-2.107287	1.884157
H	-1.158379	-3.672644	1.034233
H	1.142163	-2.362899	2.590140
H	2.522124	-1.989220	-2.186491
H	6.074478	-1.487839	-1.644435
H	4.747785	-2.483630	-2.360111
H	4.519951	-0.697832	-2.110811
H	4.419588	-1.810470	4.081480
H	2.767263	-1.140045	3.776450
H	3.024808	-2.931651	3.837231
H	0.920313	-0.604932	-3.063974
H	-0.773369	-0.832096	-3.572744
H	1.315130	0.408766	-0.677320
H	-2.525069	0.593122	-2.704611
H	-4.210404	3.152336	0.647700
H	-5.451886	2.664447	-0.566725
H	-1.857422	5.165890	1.627579
H	-0.264156	4.525835	1.062293
H	-1.549213	4.962616	-0.143483
H	2.039399	1.875975	2.591194
H	1.676161	0.448017	1.568896
H	4.192441	0.248320	1.255289

H	6.111571	0.869925	-0.192641
H	5.975137	2.892183	-1.684780
H	3.887818	4.292495	-1.686222
H	1.960847	3.672143	-0.207113
H	-1.919031	-3.403778	-3.285553
C	-4.584237	1.020692	0.548317
C	-4.742424	-1.539311	1.729183
C	-5.276780	-0.025571	-0.097644
C	-3.937916	0.759126	1.773860
C	-4.022271	-0.513736	2.365240
C	-5.360415	-1.297671	0.488218
H	-5.754010	0.163604	-1.072370
H	-3.362694	1.565262	2.255759
H	-3.523384	-0.704917	3.327891
H	-5.887583	-2.109976	-0.033651
H	-4.807226	-2.537010	2.190653

GIBBS FREE ENTHALPY

The Gibbs free enthalpy is G = H - T*S

Total enthalpy ... -1820.92944494 Eh

Total entropy correction ... -0.09617951 Eh -60.35 kcal/mol

Final Gibbs free enthalpy ... -1821.02562445 Eh

Final calculation for *Z*-**4** in hexane:

! BP RIJCOSX def2-SVP def2-SVP/J VDW10 TightOpt tightSCF COSMO(Hexane) Numfreq

%maxcore 2000

%pal nprocs 8

end

C	0.567939	-2.266850	0.791155
C	0.827188	-2.179643	-0.588820
C	-0.283038	-2.130436	-1.630321
N	-1.604327	-2.353464	-1.032344
C	-1.866553	-1.878546	0.325503
C	-0.844921	-2.463388	1.299596
C	1.647874	-2.183523	1.703206
C	2.969422	-2.023691	1.267116
C	3.238602	-1.978324	-0.136896
C	2.166061	-2.055043	-1.033767
O	4.056616	-1.874436	2.079593
O	4.548222	-1.844173	-0.484038

C	4.857672	-1.653215	-1.858469
C	3.843434	-1.840540	3.484436
C	-0.280024	-0.802266	-2.465721
C	-0.622461	0.398206	-1.617699
C	0.314069	0.880856	-0.677478
C	-0.059124	1.849050	0.269096
C	-1.374091	2.390771	0.256156
C	-2.296831	1.928983	-0.711454
C	-1.922253	0.932292	-1.632243
O	-3.568749	2.452249	-0.786519
C	-4.407115	2.230562	0.366327
O	-1.758468	3.333167	1.181721
C	-1.225517	4.643427	0.956406
O	0.767650	2.336570	1.246817
C	2.011022	1.674562	1.482086
C	3.098534	1.989461	0.469469
C	4.258053	1.188601	0.430653
C	5.287387	1.476670	-0.480165
C	5.166900	2.561302	-1.367561
C	4.008978	3.358664	-1.334974
C	2.981327	3.075881	-0.418421
C	-2.588354	-2.908779	-1.803037
O	-2.456360	-3.260399	-2.975473

H	-0.139487	-2.953958	-2.362566
H	-1.828184	-0.768804	0.354896
H	-2.900384	-2.168119	0.594004
H	-0.968806	-1.992082	2.297153
H	-1.042813	-3.550925	1.434024
H	1.427540	-2.231705	2.779354
H	2.362048	-1.995174	-2.112980
H	5.955295	-1.532725	-1.915025
H	4.557968	-2.529788	-2.477215
H	4.373860	-0.734493	-2.259213
H	4.836060	-1.686307	3.946089
H	3.169794	-1.003180	3.778732
H	3.413191	-2.795842	3.860538
H	0.715097	-0.686182	-2.943060
H	-1.020156	-0.934400	-3.279996
H	1.330938	0.470374	-0.673475
H	-2.680877	0.562897	-2.336636
H	-3.933736	2.648365	1.279270
H	-5.323672	2.820154	0.155934
H	-1.653509	5.300728	1.737466
H	-0.116901	4.649126	1.042481
H	-1.520963	5.028217	-0.045400
H	2.325690	2.026796	2.487195

H	1.857415	0.571157	1.550482
H	4.358002	0.320767	1.099891
H	6.185633	0.840127	-0.498307
H	5.973027	2.783310	-2.084490
H	3.903395	4.208039	-2.028184
H	2.069739	3.691879	-0.396390
H	-3.553175	-3.012099	-1.233212
C	-4.763405	0.769750	0.562676
C	-5.543763	-1.922322	0.938320
C	-5.224993	-0.001685	-0.525377
C	-4.668936	0.168012	1.832365
C	-5.064452	-1.168438	2.023340
C	-5.614224	-1.336795	-0.340283
H	-5.280010	0.461286	-1.522984
H	-4.286175	0.755739	2.682144
H	-4.989918	-1.624971	3.022836
H	-5.980148	-1.924242	-1.196526
H	-5.852040	-2.968904	1.084461

Calculation for TS1 in hexane:

! BP RIJCOSX def2-SVP def2-SVP/J VDW10 Opt tightSCF SlowConv NumFreq COSMO(Hexane)

! OptTS

%geom Calc_Hess true

Hybrid_Hess

[0 1 2 3 65]

end

end

%maxcore 2000

%pal nprocs 8

end

C	-0.005862	0.003719	-0.013306
N	1.466598	0.003198	-0.054797
C	2.085281	1.289001	0.013318
O	2.785611	1.647131	0.935569
C	0.259339	-2.494024	-0.521110
C	-0.532411	-1.424466	-0.068840
C	2.093381	-0.854519	-1.081998
C	1.736249	-2.310664	-0.796715
C	-0.334120	-3.768465	-0.683761
C	-1.688107	-3.991962	-0.404518
C	-2.485631	-2.912517	0.088417
C	-1.893865	-1.653072	0.245187
O	-2.346711	-5.174542	-0.590922
O	-3.787066	-3.212044	0.358599
C	-4.654456	-2.145235	0.717801

C	-1.618457	-6.266121	-1.137269
C	-0.732739	0.916163	-1.077982
C	-0.491777	0.516761	-2.515286
C	-1.071482	-0.667684	-3.021729
C	-0.736119	-1.130396	-4.304377
C	0.154281	-0.386660	-5.125827
C	0.718662	0.808144	-4.623559
C	0.390003	1.248635	-3.326360
O	1.584118	1.584927	-5.352336
C	2.751387	0.941630	-5.933107
O	0.500238	-0.847532	-6.377371
C	-0.519094	-0.710779	-7.374167
O	-1.217539	-2.283796	-4.865523
C	-2.018828	-3.153903	-4.065569
C	-3.432027	-2.656994	-3.813084
C	-4.205834	-3.259511	-2.800920
C	-5.518690	-2.824034	-2.558505
C	-6.069785	-1.775830	-3.317102
C	-5.299250	-1.170816	-4.325644
C	-3.988814	-1.612164	-4.575441
H	-0.284969	0.428644	0.977738
H	1.801052	-0.583738	-2.121807
H	3.190031	-0.717540	-1.013930

H	2.054134	-2.938693	-1.655591
H	2.310554	-2.661461	0.090231
H	0.293394	-4.590708	-1.054751
H	-2.504182	-0.811799	0.601707
H	-5.655680	-2.592683	0.857699
H	-4.341981	-1.658796	1.669360
H	-4.711522	-1.378207	-0.087621
H	-2.333601	-7.105299	-1.222865
H	-1.214596	-6.027460	-2.147386
H	-0.775116	-6.571886	-0.477904
H	-1.818270	0.880706	-0.847640
H	-0.422250	1.968744	-0.917568
H	-1.777308	-1.229896	-2.399509
H	0.876722	2.166963	-2.966814
H	2.451757	0.315385	-6.796784
H	3.365368	1.789360	-6.295789
H	-0.080861	-1.062182	-8.327989
H	-1.407091	-1.333177	-7.131411
H	-0.830214	0.351411	-7.484024
H	-2.051627	-4.103977	-4.639055
H	-1.510294	-3.369629	-3.096770
H	-3.780112	-4.062971	-2.179693
H	-6.107650	-3.304145	-1.762235

H	-7.095741	-1.428271	-3.120334
H	-5.719759	-0.345060	-4.920795
H	-3.381740	-1.131592	-5.357306
H	1.915639	1.969311	-0.875629
C	3.516819	0.118268	-4.920861
C	4.902576	-1.405550	-2.983860
C	4.388310	0.743915	-4.004297
C	3.325174	-1.277187	-4.836349
C	4.014994	-2.034947	-3.873726
C	5.083280	-0.011345	-3.046087
H	4.529311	1.835874	-4.053077
H	2.625545	-1.764073	-5.533742
H	3.860378	-3.124572	-3.821046
H	5.769075	0.488392	-2.343922
H	5.446904	-1.999342	-2.232752

GIBBS FREE ENTHALPY

The Gibbs free enthalpy is G = H - T*S

Total enthalpy ... -1820.88791636 Eh

Total entropy correction ... -0.09748231 Eh -61.17 kcal/mol

Final Gibbs free enthalpy ... -1820.98539867 Eh

Calculation for TS2 in hexane:

```
! BP RIJCOSX def2-SVP def2-SVP/J VDW10 Opt tightSCF SlowConv NumFreq COSMO(Hexane)

! OptTS

%geom Calc_Hess true

Hybrid_Hess

[0 1 2 3 65]

end

end

%maxcore 2000

%pal nprocs 8

end


C -0.004666 0.011430 -0.012190

N 1.465669 0.016645 -0.097090

C 2.087599 1.301090 -0.044371

O 2.040754 2.155167 -0.908949

C 0.265893 -2.514316 -0.333427

C -0.514388 -1.421166 0.080302
```

C	2.079901	-0.909559	-1.069094
C	1.730600	-2.344855	-0.679104
C	-0.325730	-3.799115	-0.385178
C	-1.664345	-4.009308	-0.030205
C	-2.444401	-2.904930	0.433911
C	-1.856739	-1.634397	0.475272
O	-2.324272	-5.203608	-0.112792
O	-3.727042	-3.193444	0.793130
C	-4.585076	-2.110145	1.123381
C	-1.622769	-6.318653	-0.646571
C	-0.766832	0.803299	-1.141127
C	-0.587363	0.249586	-2.536361
C	-1.240189	-0.944922	-2.912958
C	-0.978744	-1.529616	-4.162537
C	-0.097400	-0.898097	-5.080441
C	0.552290	0.298253	-4.699115
C	0.304820	0.860624	-3.432466
O	1.432846	0.958070	-5.521622
C	2.527560	0.197771	-6.097216
O	0.164420	-1.476397	-6.304510
C	-0.893562	-1.361485	-7.261671
O	-1.525251	-2.708115	-4.603094
C	-2.284573	-3.494762	-3.686898

C	-3.678365	-2.962668	-3.401493
C	-4.395328	-3.458857	-2.294350
C	-5.693053	-2.994177	-2.025517
C	-6.284142	-2.021703	-2.852028
C	-5.568888	-1.521380	-3.954224
C	-4.274395	-1.992790	-4.230267
H	-0.264052	0.529842	0.940840
H	1.773677	-0.706176	-2.118173
H	3.178345	-0.765444	-1.032126
H	2.014831	-3.024224	-1.509524
H	2.338488	-2.645688	0.204405
H	0.288921	-4.641776	-0.731313
H	-2.456536	-0.772322	0.798809
H	-5.572667	-2.553475	1.348313
H	-4.227445	-1.555911	2.020617
H	-4.693150	-1.401852	0.271027
H	-2.339070	-7.161365	-0.655655
H	-1.273336	-6.125875	-1.686269
H	-0.745063	-6.594438	-0.019676
H	-1.844007	0.801181	-0.868565
H	-0.431448	1.857406	-1.108015
H	-1.942315	-1.419880	-2.216649
H	0.876143	1.750847	-3.136706

H	2.151936	-0.471892	-6.896527
H	3.177764	0.972361	-6.549915
H	-0.520434	-1.805087	-8.204869
H	-1.798401	-1.916809	-6.932210
H	-1.158992	-0.295981	-7.438989
H	-2.357353	-4.491599	-4.171313
H	-1.724104	-3.629279	-2.732586
H	-3.934733	-4.199354	-1.621801
H	-6.239552	-3.391421	-1.156604
H	-7.298169	-1.651732	-2.635687
H	-6.020826	-0.755511	-4.604132
H	-3.709480	-1.595546	-5.086889
H	2.670731	1.483549	0.903970
C	3.286796	-0.596055	-5.056465
C	4.655957	-2.053228	-3.057472
C	4.239765	0.038014	-4.231798
C	3.008330	-1.964013	-4.850313
C	3.689570	-2.688372	-3.856665
C	4.927214	-0.685447	-3.244248
H	4.445882	1.110989	-4.372402
H	2.245916	-2.454465	-5.475298
H	3.465469	-3.756473	-3.707602
H	5.676395	-0.179761	-2.614997

H 5.191227 -2.621367 -2.280423

GIBBS FREE ENTHALPY

The Gibbs free enthalpy is G = H - T*S

Total enthalpy ... -1820.88950686 Eh

Total entropy correction ... -0.09715150 Eh -60.96 kcal/mol

Final Gibbs free enthalpy ... -1820.98665836 Eh

Final calculation for *E*-**4** in gas phase:

! BP RIJCOSX def2-SVP def2-SVP/J VDW10 TightOpt tightSCF NumFreq

%maxcore 2000

%pal nprocs 8

end

C 0.494837 -2.356924 0.549165

C 0.864888 -2.228501 -0.801647

C -0.165477 -2.149253 -1.924494

N -1.515853 -2.448115 -1.444309

C	-1.911592	-2.014653	-0.103710
C	-0.950275	-2.578815	0.939923
C	1.498238	-2.291087	1.545557
C	2.847891	-2.101845	1.223685
C	3.228344	-2.004227	-0.151250
C	2.232268	-2.069778	-1.133030
O	3.863313	-1.966348	2.126201
O	4.558725	-1.835889	-0.386828
C	4.972163	-1.589935	-1.721501
C	3.540744	-2.001604	3.507202
C	-0.117222	-0.770265	-2.676323
C	-0.545344	0.386089	-1.805324
C	0.297582	0.824420	-0.760879
C	-0.167874	1.757389	0.178534
C	-1.470889	2.313421	0.054910
C	-2.298154	1.897456	-1.014304
C	-1.833462	0.928044	-1.925695
O	-3.552878	2.405363	-1.226910
C	-4.494836	2.387554	-0.124434
O	-1.936555	3.219965	0.981384
C	-1.363585	4.526994	0.886012
O	0.560461	2.205237	1.250366
C	1.795043	1.558835	1.553749

C	2.948333	1.927982	0.635617
C	4.119148	1.143315	0.645606
C	5.204869	1.481443	-0.178158
C	5.130424	2.599554	-1.027478
C	3.961705	3.380812	-1.043738
C	2.877636	3.048506	-0.213721
C	-2.427118	-3.077892	-2.254569
O	-3.595076	-3.322031	-1.971104
H	0.087956	-2.926762	-2.680053
H	-1.940101	-0.906795	-0.046903
H	-2.942714	-2.375060	0.049977
H	-1.164253	-2.111879	1.924301
H	-1.133327	-3.670572	1.060130
H	1.191918	-2.375531	2.597961
H	2.514028	-1.972362	-2.190516
H	6.068361	-1.448588	-1.685686
H	4.741220	-2.448287	-2.394067
H	4.504233	-0.665209	-2.128614
H	4.491554	-1.857268	4.052854
H	2.832127	-1.188126	3.787831
H	3.098016	-2.979085	3.805043
H	0.912963	-0.615411	-3.060376
H	-0.782229	-0.848775	-3.560658

H	1.310308	0.413451	-0.677096
H	-2.528745	0.589625	-2.707290
H	-4.223415	3.153824	0.628809
H	-5.455673	2.672822	-0.597643
H	-1.876692	5.154928	1.639633
H	-0.274554	4.508861	1.107742
H	-1.527389	4.964260	-0.125043
H	2.027544	1.881771	2.591019
H	1.661342	0.450828	1.573078
H	4.186854	0.250283	1.284619
H	6.111700	0.857267	-0.156883
H	5.981219	2.861059	-1.675935
H	3.891801	4.256578	-1.707763
H	1.957310	3.651517	-0.231119
H	-1.967758	-3.366275	-3.245352
C	-4.602073	1.023839	0.520088
C	-4.787571	-1.534451	1.696341
C	-5.249106	-0.031538	-0.156940
C	-4.009289	0.771744	1.773580
C	-4.109376	-0.500533	2.363506
C	-5.345303	-1.303715	0.425268
H	-5.676899	0.150118	-1.155513
H	-3.461416	1.584054	2.276788

H -3.654987 -0.684922 3.349307

H -5.827545 -2.124890 -0.124600

H -4.863828 -2.532002 2.156444

GIBBS FREE ENTHALPY

The Gibbs free enthalpy is G = H - T*S

Total enthalpy ... -1820.92167468 Eh

Total entropy correction ... -0.09380057 Eh -58.86 kcal/mol

Final Gibbs free enthalpy ... -1821.01547526 Eh

Final calculation for *Z*-**4** in gas phase:

! BP RIJCOSX def2-SVP def2-SVP/J VDW10 TightOpt tightSCF NumFreq

%maxcore 2000

%pal nprocs 8

end

C 0.567591 -2.261386 0.787477

C 0.828372 -2.167533 -0.591619

C	-0.280385	-2.124804	-1.634890
N	-1.601321	-2.349966	-1.038823
C	-1.866489	-1.878810	0.318486
C	-0.845400	-2.465576	1.292404
C	1.645747	-2.176996	1.701307
C	2.967310	-2.009122	1.269032
C	3.238590	-1.955198	-0.133937
C	2.167504	-2.033520	-1.032262
O	4.052843	-1.859646	2.084512
O	4.547757	-1.812890	-0.478487
C	4.857785	-1.616334	-1.849422
C	3.840148	-1.854212	3.486509
C	-0.280970	-0.799764	-2.475422
C	-0.627753	0.401240	-1.630550
C	0.309055	0.891526	-0.695229
C	-0.065268	1.860317	0.249837
C	-1.382713	2.395641	0.240606
C	-2.307178	1.924399	-0.721308
C	-1.930370	0.927583	-1.640818
O	-3.582891	2.437895	-0.792268
C	-4.410733	2.221264	0.366308
O	-1.773078	3.336508	1.164886
C	-1.225862	4.642060	0.962179

O	0.765486	2.354104	1.221905
C	1.999778	1.682520	1.468567
C	3.099683	1.983174	0.464562
C	4.261903	1.185827	0.455194
C	5.301951	1.461774	-0.446621
C	5.189202	2.529198	-1.355066
C	4.028170	3.321877	-1.352486
C	2.990200	3.052520	-0.444228
C	-2.587139	-2.891766	-1.822431
O	-2.451692	-3.226380	-2.996434
H	-0.134247	-2.950618	-2.364535
H	-1.829513	-0.768828	0.353608
H	-2.900613	-2.170404	0.584649
H	-0.972772	-1.999824	2.292391
H	-1.039555	-3.554696	1.420281
H	1.423255	-2.232221	2.776745
H	2.364786	-1.967930	-2.110870
H	5.955106	-1.490812	-1.904157
H	4.563879	-2.492397	-2.472658
H	4.371994	-0.697804	-2.249195
H	4.831726	-1.702713	3.951941
H	3.160967	-1.027436	3.800002
H	3.416018	-2.819319	3.846065

H	0.713741	-0.680022	-2.953158
H	-1.020314	-0.939952	-3.288946
H	1.328389	0.487195	-0.694134
H	-2.689419	0.552922	-2.341815
H	-3.932190	2.643581	1.274813
H	-5.330162	2.808315	0.160009
H	-1.667853	5.296590	1.737987
H	-0.119611	4.640211	1.074492
H	-1.495201	5.037893	-0.043222
H	2.310587	2.035334	2.475089
H	1.836546	0.580381	1.539308
H	4.356365	0.328944	1.139386
H	6.202363	0.828120	-0.440411
H	6.004035	2.741872	-2.064907
H	3.928168	4.157406	-2.063092
H	2.075544	3.664066	-0.444621
H	-3.554236	-2.997617	-1.254798
C	-4.765490	0.760936	0.571537
C	-5.543893	-1.929247	0.961450
C	-5.231915	-0.014926	-0.510942
C	-4.664382	0.163979	1.842622
C	-5.058794	-1.171458	2.040735
C	-5.620557	-1.348893	-0.318818

H -5.289993 0.445016 -1.509711

H -4.276686 0.755500 2.687466

H -4.979931 -1.624086 3.041667

H -5.990741 -1.939569 -1.170936

H -5.852500 -2.974871 1.113480

GIBBS FREE ENTHALPY

The Gibbs free enthalpy is G = H - T*S

Total enthalpy ... -1820.92143411 Eh

Total entropy correction ... -0.09626242 Eh -60.41 kcal/mol

Final Gibbs free enthalpy ... -1821.01769653 Eh

Calculation for TS1 in gas phase:

! BP RIJCOSX def2-SVP def2-SVP/J VDW10 Opt tightSCF SlowConv NumFreq

! OptTS

%geom Calc_Hess true

Hybrid_Hess

[0 1 2 3 65]

end

end

%maxcore 2000

%pal nprocs 8

end

C	-0.006596	0.007922	-0.022393
N	1.462246	0.010078	-0.079310
C	2.086470	1.293884	0.001555
O	2.781561	1.646074	0.927672
C	0.257663	-2.490713	-0.525020
C	-0.533897	-1.420304	-0.074912
C	2.087229	-0.852588	-1.099610
C	1.734322	-2.307286	-0.800706
C	-0.336469	-3.764945	-0.684926
C	-1.690034	-3.988486	-0.404580
C	-2.487064	-2.908589	0.086875
C	-1.894569	-1.649240	0.240209
O	-2.349256	-5.171486	-0.589379
O	-3.788359	-3.207166	0.358536
C	-4.652556	-2.141525	0.718689
C	-1.620322	-6.264983	-1.122390
C	-0.742778	0.921274	-1.081156

C	-0.501758	0.524710	-2.518789
C	-1.081556	-0.658675	-3.026799
C	-0.742041	-1.122361	-4.307705
C	0.153871	-0.381177	-5.125752
C	0.719601	0.812372	-4.621152
C	0.384624	1.254065	-3.326158
O	1.590762	1.587567	-5.343230
C	2.755661	0.945214	-5.923482
O	0.510562	-0.842687	-6.374315
C	-0.507725	-0.743357	-7.373642
O	-1.224291	-2.275707	-4.869459
C	-2.023730	-3.147118	-4.071226
C	-3.436596	-2.652424	-3.810886
C	-4.204979	-3.257850	-2.796636
C	-5.517760	-2.825595	-2.549350
C	-6.073440	-1.777423	-3.303905
C	-5.307930	-1.169294	-4.313909
C	-3.998199	-1.607915	-4.569686
H	-0.277647	0.430536	0.972445
H	1.793306	-0.593621	-2.142319
H	3.183744	-0.712588	-1.034982
H	2.054686	-2.943526	-1.652844
H	2.307353	-2.645911	0.091721

H	0.291634	-4.587889	-1.053618
H	-2.504357	-0.807255	0.595948
H	-5.654022	-2.588056	0.861495
H	-4.338825	-1.654505	1.670232
H	-4.712729	-1.373774	-0.086386
H	-2.334880	-7.105277	-1.205137
H	-1.209995	-6.036460	-2.133008
H	-0.779518	-6.567494	-0.457287
H	-1.828088	0.883552	-0.849517
H	-0.432961	1.974019	-0.919388
H	-1.790386	-1.219672	-2.406958
H	0.871633	2.171922	-2.966186
H	2.457615	0.320499	-6.789137
H	3.370704	1.793270	-6.284303
H	-0.055679	-1.088942	-8.323362
H	-1.378750	-1.389034	-7.130247
H	-0.848663	0.308941	-7.494049
H	-2.059817	-4.093785	-4.650769
H	-1.512803	-3.369312	-3.104847
H	-3.776811	-4.061938	-2.177992
H	-6.102483	-3.309722	-1.752453
H	-7.099518	-1.432472	-3.103383
H	-5.732167	-0.343039	-4.905546

H -3.393501 -1.124927 -5.351776

H 1.921451 1.978468 -0.886777

C 3.522284 0.118344 -4.914433

C 4.914577 -1.414110 -2.989601

C 4.398649 0.739162 -3.999620

C 3.328187 -1.276742 -4.833370

C 4.021400 -2.038554 -3.876821

C 5.097010 -0.020254 -3.047659

H 4.540487 1.831110 -4.045378

H 2.623675 -1.759140 -5.529045

H 3.865660 -3.128179 -3.827643

H 5.787174 0.475946 -2.347372

H 5.462644 -2.011492 -2.244113

GIBBS FREE ENTHALPY

The Gibbs free enthalpy is G = H - T*S

Total enthalpy ... -1820.87927160 Eh

Total entropy correction ... -0.09753911 Eh -61.21 kcal/mol

Final Gibbs free enthalpy ... -1820.97681070 Eh

Calculation for TS2 in gas phase:

```
! BP RIJCOSX def2-SVP def2-SVP/J VDW10 Opt tightSCF SlowConv NumFreq

! OptTS

%geom Calc_Hess true

Hybrid_Hess

[0 1 2 3 65]

end

end

%maxcore 2000

%pal nprocs 8

end
```

```
C -0.005853 0.014183 -0.017951
N 1.462795 0.018588 -0.106477
C 2.087716 1.300798 -0.041040
O 2.048820 2.162491 -0.896718
C 0.264262 -2.511845 -0.337530
C -0.518039 -1.417998 0.069621
C 2.077693 -0.909510 -1.074131
C 1.730534 -2.342944 -0.675700
C -0.327272 -3.796414 -0.389034
```

C	-1.667090	-4.006887	-0.039782
C	-2.450182	-2.901654	0.416058
C	-1.862335	-1.631263	0.456554
O	-2.325564	-5.202446	-0.121626
O	-3.734468	-3.188869	0.769031
C	-4.592775	-2.106202	1.089520
C	-1.616529	-6.320168	-0.630678
C	-0.766337	0.814610	-1.142144
C	-0.585958	0.265801	-2.539021
C	-1.244750	-0.923515	-2.920440
C	-0.983165	-1.506838	-4.170182
C	-0.095557	-0.878495	-5.084031
C	0.560967	0.312744	-4.697295
C	0.312883	0.873606	-3.429985
O	1.448575	0.968961	-5.513529
C	2.536261	0.205680	-6.091783
O	0.172461	-1.455050	-6.307804
C	-0.888145	-1.370412	-7.262208
O	-1.537482	-2.681507	-4.613605
C	-2.282172	-3.475134	-3.693797
C	-3.679261	-2.958359	-3.394576
C	-4.393711	-3.490563	-2.302838
C	-5.693718	-3.040916	-2.021433

C	-6.289356	-2.047642	-2.818866
C	-5.576224	-1.511012	-3.904730
C	-4.279718	-1.967556	-4.194381
H	-0.262616	0.526862	0.939585
H	1.771121	-0.712533	-2.124515
H	3.176067	-0.763497	-1.038899
H	2.021629	-3.028245	-1.498986
H	2.332842	-2.633471	0.215049
H	0.290233	-4.639429	-0.729284
H	-2.463982	-0.768456	0.774618
H	-5.581620	-2.548483	1.311767
H	-4.240902	-1.547095	1.986778
H	-4.698862	-1.401440	0.233507
H	-2.329715	-7.165860	-0.635527
H	-1.255159	-6.143136	-1.669784
H	-0.744128	-6.585855	0.008963
H	-1.844239	0.815273	-0.871831
H	-0.426539	1.867222	-1.104124
H	-1.953048	-1.395200	-2.228267
H	0.887612	1.760896	-3.131885
H	2.158855	-0.457570	-6.895899
H	3.192648	0.978500	-6.539145
H	-0.503753	-1.805551	-8.205076

H	-1.778016	-1.948924	-6.931841
H	-1.181693	-0.312396	-7.442439
H	-2.351053	-4.472240	-4.179035
H	-1.713177	-3.606646	-2.743749
H	-3.929336	-4.246254	-1.650100
H	-6.237490	-3.466974	-1.164615
H	-7.305580	-1.689787	-2.592734
H	-6.031628	-0.727892	-4.531031
H	-3.715436	-1.542635	-5.037812
H	2.665311	1.471920	0.914273
C	3.290487	-0.599470	-5.055509
C	4.650371	-2.075853	-3.064865
C	4.248532	0.023270	-4.228724
C	3.001303	-1.965909	-4.855205
C	3.678084	-2.699375	-3.865530
C	4.931732	-0.709672	-3.245662
H	4.461183	1.095575	-4.363914
H	2.233794	-2.446828	-5.481368
H	3.446444	-3.766566	-3.721477
H	5.686258	-0.212680	-2.615881
H	5.183220	-2.652020	-2.292060

GIBBS FREE ENTHALPY

The Gibbs free enthalpy is G = H - T*S

Total enthalpy ... -1820.88151974 Eh

Total entropy correction ... -0.09729213 Eh -61.05 kcal/mol

Final Gibbs free enthalpy ... -1820.97881188 Eh

Calculation for GS_{DMF} in gas phase:

```
! BP RIJCOSX def2-SVP def2-SVP/J VDW10 TightOpt tightSCF NumFreq
%maxcore 1000



  C       0.136976      0.012695      1.148585
  H      -0.603801      0.718124      1.626621
  N      -0.232927     -1.299828      1.339160
  O       1.131188      0.394070      0.545299
  C       0.586702     -2.371386      0.799978
  H       1.453863     -1.913503      0.288089
  H       0.011373     -2.986195      0.072681
  H       0.949811     -3.041382      1.610109
  C      -1.434190     -1.660010      2.064355
  H      -1.199484     -2.285889      2.954557
  H      -2.140406     -2.233606      1.421983
  H      -1.949504     -0.742569      2.412449



-------------------
GIBBS FREE ENTHALPY
-------------------

The Gibbs free enthalpy is G = H - T*S

Total enthalpy                  ...   -248.23586708 Eh
Total entropy correction        ...     -0.03496376 Eh    -21.94 kcal/mol
-----------------------------------------------------------------------
Final Gibbs free enthalpy       ...   -248.27083084 Eh
```

Calculation for $TS1_{DMF}$ in gas phase:

```
! BP RIJCOSX def2-SVP def2-SVP/J VDW10 Opt tightSCF Slowconv NumFreq
! OptTS
%geom Calc_Hess true
end
%maxcore 1000



  C       0.008838      0.003469      0.024370
  H       1.132329     -0.002101      0.229378
  N      -0.576919      1.320711      0.024778
  O      -0.599811     -1.019240     -0.169752
  C      -0.416543      1.934250      1.345940
  H      -0.913651      1.311648      2.116460
  H      -0.908939      2.927640      1.345254
  H       0.654743      2.085202      1.648065
  C       0.022604      2.131376     -1.038171
  H       1.126649      2.297544     -0.912760
  H      -0.467774      3.125326     -1.055091
  H      -0.149395      1.652993     -2.023486


-------------------
GIBBS FREE ENTHALPY
-------------------

The Gibbs free enthalpy is G = H - T*S

Total enthalpy                    ...   -248.19912857 Eh
Total entropy correction          ...     -0.03372834 Eh    -21.16 kcal/mol
-----------------------------------------------------------------------
Final Gibbs free enthalpy         ...   -248.23285691 Eh
```

Calculation for $TS2_{DMF}$ in gas phase:

```
! BP RIJCOSX def2-SVP def2-SVP/J VDW10 Opt tightSCF Slowconv NumFreq
! OptTS
%geom Calc_Hess true
end
%maxcore 1000



  C      -0.008278     -0.001163      0.022904
  H       1.101627      0.007543      0.225479
  N      -0.593822      1.322401     -0.007597
  O      -0.616386     -1.034706     -0.146713
  C      -1.146446      1.620594     -1.334046
  H      -0.360115      1.498905     -2.107641
  H      -1.479524      2.678410     -1.356270
  H      -2.012673      0.969735     -1.612149
  C      -1.584727      1.482597      1.062658
  H      -2.479924      0.822607      0.943674
  H      -1.925654      2.537835      1.084444
  H      -1.120233      1.259496      2.045524


-------------------
```

```
GIBBS FREE ENTHALPY
-------------------

The Gibbs free enthalpy is G = H - T*S

Total enthalpy                    ...   -248.19819470 Eh
Total entropy correction          ...     -0.03377372 Eh    -21.19 kcal/mol
-----------------------------------------------------------------------
Final Gibbs free enthalpy         ...   -248.23196842 Eh
```

Calculation for GS_{DMF} in hexane:

```
! BP RIJCOSX def2-SVP def2-SVP/J VDW10 TightOpt tightSCF NumFreq COSMO(Hexane)
%maxcore 1000


  C       0.134247      0.011780      1.149900
  H      -0.605208      0.716556      1.627313
  N      -0.231452     -1.297516      1.338320
  O       1.130895      0.397913      0.545627
  C       0.587064     -2.371821      0.799873
  H       1.455933     -1.919578      0.286529
  H       0.008506     -2.985659      0.074969
  H       0.947081     -3.040352      1.612000
  C      -1.434182     -1.659158      2.064301
  H      -1.195920     -2.286030      2.952082
  H      -2.137239     -2.232687      1.419467
  H      -1.950123     -0.742928      2.413486


-------------------
GIBBS FREE ENTHALPY
-------------------

The Gibbs free enthalpy is G = H - T*S

Total enthalpy                    ...   -248.23919662 Eh
Total entropy correction          ...     -0.03494177 Eh    -21.93 kcal/mol
-----------------------------------------------------------------------
Final Gibbs free enthalpy         ...   -248.27413839 Eh
```

Calculation for $TS1_{DMF}$ in hexane:

```
! BP RIJCOSX def2-SVP def2-SVP/J VDW10 Opt tightSCF Slowconv NumFreq COSMO(Hexane)
! OptTS
%geom Calc_Hess true
end
%maxcore 1000



  C       0.008885      0.006053      0.024605
  H       1.130385     -0.000698      0.229030
  N      -0.581467      1.319183      0.023778
  O      -0.600795     -1.018553     -0.169701
  C      -0.416634      1.934151      1.346807
  H      -0.909330      1.310807      2.119414
  H      -0.909958      2.926864      1.345816
  H       0.655545      2.085455      1.642002
  C       0.022863      2.131443     -1.039028
  H       1.125301      2.296758     -0.906883
```

```
  H      -0.468374      3.124770     -1.056009
  H      -0.144289      1.652588     -2.024848
```

```
-------------------
GIBBS FREE ENTHALPY
-------------------

The Gibbs free enthalpy is G = H - T*S

Total enthalpy                    ...   -248.20202857 Eh
Total entropy correction          ...     -0.03371222 Eh    -21.15 kcal/mol
-----------------------------------------------------------------------
Final Gibbs free enthalpy         ...   -248.23574080 Eh
```

Calculation for TS2$_{DMF}$ in hexane:

```
! BP RIJCOSX def2-SVP def2-SVP/J VDW10 Opt tightSCF Slowconv NumFreq COSMO(Hexane)
! OptTS
%geom Calc_Hess true
end
%maxcore 1000
```

```
  C      -0.007097      0.001598      0.023266
  H       1.101764      0.004965      0.225354
  N      -0.590811      1.324768     -0.006897
  O      -0.619230     -1.031329     -0.147101
  C      -1.145925      1.620710     -1.334795
  H      -0.360794      1.497259     -2.109123
  H      -1.480434      2.677846     -1.357177
  H      -2.012451      0.968845     -1.608495
  C      -1.584527      1.482603      1.063571
  H      -2.478354      0.821986      0.940333
  H      -1.926951      2.537149      1.084815
  H      -1.121345      1.257855      2.046516
```

```
-------------------
GIBBS FREE ENTHALPY
-------------------

The Gibbs free enthalpy is G = H - T*S

Total enthalpy                    ...   -248.20063675 Eh
Total entropy correction          ...     -0.03374628 Eh    -21.18 kcal/mol
-----------------------------------------------------------------------
Final Gibbs free enthalpy         ...   -248.23438303 Eh
```

Calculation for GS$_{DMF}$ in ethanol:

```
! BP RIJCOSX def2-SVP def2-SVP/J VDW10 TightOpt tightSCF NumFreq COSMO(Ethanol)
%maxcore 1000
```

```
  C       0.129324      0.009916      1.152799
  H      -0.607682      0.713933      1.628720
  N      -0.228794     -1.292841      1.336437
  O       1.130833      0.405868      0.545959
  C       0.587305     -2.372603      0.799550
```

```
  H       1.459925     -1.930962      0.284415
  H       0.002403     -2.983953      0.078934
  H       0.939792     -3.038952      1.615594
  C      -1.434132     -1.657886      2.064398
  H      -1.187767     -2.285487      2.947872
  H      -2.129763     -2.232696      1.414849
  H      -1.951843     -0.743815      2.414341
```

```
-------------------
GIBBS FREE ENTHALPY
-------------------

The Gibbs free enthalpy is G = H - T*S

Total enthalpy                    ...   -248.24570009 Eh
Total entropy correction          ...     -0.03488447 Eh    -21.89 kcal/mol
-----------------------------------------------------------------------
Final Gibbs free enthalpy         ...   -248.28058455 Eh
```

Calculation for TS1$_{DMF}$ in ethanol:

```
! BP RIJCOSX def2-SVP def2-SVP/J VDW10 Opt tightSCF Slowconv NumFreq COSMO(Ethanol)
! OptTS
%geom Calc_Hess true
end
%maxcore 1000
```

```
  C       0.009732      0.010895      0.025177
  H       1.127480      0.002345      0.228789
  N      -0.590075      1.315658      0.021886
  O      -0.602180     -1.017524     -0.169905
  C      -0.416838      1.933878      1.348319
  H      -0.902464      1.310201      2.124954
  H      -0.910913      2.925838      1.345922
  H       0.656639      2.085055      1.631245
  C       0.023226      2.131396     -1.040536
  H       1.122577      2.294555     -0.896737
  H      -0.469103      3.123858     -1.056510
  H      -0.135953      1.652665     -2.027621
```

```
-------------------
GIBBS FREE ENTHALPY
-------------------

The Gibbs free enthalpy is G = H - T*S

Total enthalpy                    ...   -248.20742042 Eh
Total entropy correction          ...     -0.03365155 Eh    -21.12 kcal/mol
-----------------------------------------------------------------------
Final Gibbs free enthalpy         ...   -248.24107196 Eh
```

Calculation for TS2$_{DMF}$ in ethanol:

```
! BP RIJCOSX def2-SVP def2-SVP/J VDW10 Opt tightSCF Slowconv NumFreq COSMO(Ethanol)
! OptTS
%geom Calc_Hess true
```

```
end
%maxcore 1000
```

```
  C      -0.004913      0.006279      0.023892
  H       1.102172      0.001116      0.225139
  N      -0.586402      1.327894     -0.005869
  O      -0.623413     -1.026355     -0.147665
  C      -1.145048      1.620787     -1.336207
  H      -0.361748      1.494596     -2.111649
  H      -1.481716      2.676872     -1.358548
  H      -2.012468      0.967931     -1.603143
  C      -1.584264      1.482491      1.065243
  H      -2.476399      0.821458      0.935388
  H      -1.928858      2.535991      1.085379
  H      -1.123097      1.255195      2.048307
```

```
-------------------
GIBBS FREE ENTHALPY
-------------------

The Gibbs free enthalpy is G = H - T*S

Total enthalpy                    ...   -248.20488768 Eh
Total entropy correction          ...     -0.03370613 Eh    -21.15 kcal/mol
-----------------------------------------------------------------------
Final Gibbs free enthalpy         ...   -248.23859381 Eh
```

Calculation for GS_{DMF} in DMSO:

```
! BP RIJCOSX def2-SVP def2-SVP/J VDW10 TightOpt tightSCF NumFreq COSMO(DMSO)
%maxcore 1000
```

```
  C       0.129047      0.009871      1.152973
  H      -0.607981      0.713638      1.628852
  N      -0.228568     -1.292526      1.336309
  O       1.130837      0.406431      0.545916
  C       0.587316     -2.372632      0.799550
  H       1.460218     -1.931757      0.284286
  H       0.002030     -2.983862      0.079217
  H       0.939298     -3.038899      1.615812
  C      -1.434042     -1.657770      2.064357
  H      -1.187313     -2.285469      2.947572
  H      -2.129322     -2.232613      1.414578
  H      -1.951918     -0.743891      2.414444
```

```
-------------------
GIBBS FREE ENTHALPY
-------------------
```

```
The Gibbs free enthalpy is G = H - T*S

Total enthalpy                    ...   -248.24609951 Eh
Total entropy correction          ...     -0.03488440 Eh    -21.89 kcal/mol
-----------------------------------------------------------------------
Final Gibbs free enthalpy         ...   -248.28098391 Eh
```

Calculation for $TS1_{DMF}$ in DMSO:

```
! BP RIJCOSX def2-SVP def2-SVP/J VDW10 Opt tightSCF Slowconv NumFreq COSMO(DMSO)
! OptTS
%geom Calc_Hess true
end
%maxcore 1000



  C       0.009785      0.011169      0.025211
  H       1.127329      0.002557      0.228731
  N      -0.590619      1.315433      0.021762
  O      -0.602296     -1.017479     -0.169864
  C      -0.416837      1.933849      1.348390
  H      -0.902080      1.310187      2.125265
  H      -0.910901      2.925790      1.345901
  H       0.656709      2.084974      1.630587
  C       0.023244      2.131396     -1.040605
  H       1.122383      2.294492     -0.896072
  H      -0.469188      3.123785     -1.056554
  H      -0.135398      1.652666     -2.027768


-------------------
GIBBS FREE ENTHALPY
-------------------

The Gibbs free enthalpy is G = H - T*S

Total enthalpy                    ...   -248.20775769 Eh
Total entropy correction          ...     -0.03366877 Eh    -21.13 kcal/mol
-----------------------------------------------------------------------
Final Gibbs free enthalpy         ...   -248.24142646 Eh
```

Calculation for $TS2_{DMF}$ in DMSO:

```
! BP RIJCOSX def2-SVP def2-SVP/J VDW10 Opt tightSCF Slowconv NumFreq COSMO(DMSO)
! OptTS
%geom Calc_Hess true
end
%maxcore 1000



  C      -0.004763      0.006556      0.023934
  H       1.102232      0.001021      0.225120
  N      -0.586232      1.328002     -0.005826
  O      -0.623497     -1.026159     -0.147661
  C      -1.145018      1.620778     -1.336300
  H      -0.361823      1.494342     -2.111797
  H      -1.481681      2.676848     -1.358654
  H      -2.012577      0.967980     -1.602877
  C      -1.584279      1.482468      1.065342
  H      -2.476410      0.821524      0.935115
```

```
  H      -1.928886      2.535949      1.085474
  H      -1.123220      1.254945      2.048397


-------------------
GIBBS FREE ENTHALPY
-------------------

The Gibbs free enthalpy is G = H - T*S

Total enthalpy                    ...   -248.20512402 Eh
Total entropy correction          ...     -0.03370632 Eh    -21.15 kcal/mol
-----------------------------------------------------------------------
Final Gibbs free enthalpy         ...   -248.23883034 Eh
```
